# Spatio-Temporal Gap Analysis of OBIS-SEAMAP Project Data: Assessment and Way Forward

**DOI:** 10.1371/journal.pone.0012990

**Published:** 2010-09-24

**Authors:** Connie Y. Kot, Ei Fujioka, Lucie J. Hazen, Benjamin D. Best, Andrew J. Read, Patrick N. Halpin

**Affiliations:** 1 Marine Geospatial Ecology Lab, Nicholas School of the Environment, Duke University, Beaufort, North Carolina, United States of America; 2 Nicholas School of the Environment, Duke University, Beaufort, North Carolina, United States of America; Institut Pluridisciplinaire Hubert Curien, France

## Abstract

The OBIS-SEAMAP project has acquired and served high-quality marine mammal, seabird, and sea turtle data to the public since its inception in 2002. As data accumulated, spatial and temporal biases resulted and a comprehensive gap analysis was needed in order to assess coverage to direct data acquisition for the OBIS-SEAMAP project and for taxa researchers should true gaps in knowledge exist. All datasets published on OBIS-SEAMAP up to February 2009 were summarized spatially and temporally. Seabirds comprised the greatest number of records, compared to the other two taxa, and most records were from shipboard surveys, compared to the other three platforms. Many of the point observations and polyline tracklines were located in northern and central Atlantic and the northeastern and central-eastern Pacific. The Southern Hemisphere generally had the lowest representation of data, with the least number of records in the southern Atlantic and western Pacific regions. Temporally, records of observations for all taxa were the lowest in fall although the number of animals sighted was lowest in the winter. Oceanographic coverage of observations varied by platform for each taxa, which showed that using two or more platforms represented habitat ranges better than using only one alone. Accessible and published datasets not already incorporated do exist within spatial and temporal gaps identified. Other related open-source data portals also contain data that fill gaps, emphasizing the importance of dedicated data exchange. Temporal and spatial gaps were mostly a result of data acquisition effort, development of regional partnerships and collaborations, and ease of field data collection. Future directions should include fostering partnerships with researchers in the Southern Hemisphere while targeting datasets containing species with limited representation. These results can facilitate prioritizing datasets needed to be represented and for planning research for true gaps in space and time.

## Introduction

The need for the conservation of marine mammals, seabirds, and sea turtles, is increasing due to the on-going and long-term negative effects of direct harvests/kills, indirect fisheries catch, and habitat alteration and degradation. A detailed understanding of the spatial and temporal patterns of species distribution and diversity is critical for quantifying populations, the significance of adverse events, and the potential for mitigation. Resources may be limited for researchers to gather enough information over long periods of time, across large regions, and on multiple species to fully assess conservation requirements. Therefore, a world data commons where multiple datasets are available can facilitate this fundamental need.

Established by the Census of Marine Life program [1] in 2002, Duke University is leading the OBIS-SEAMAP project (Ocean Biogeographic Information System – Spatial Ecological Analysis of Megavertebrate Populations project, herein called SEAMAP) involving a consortium of organizations and individuals who share a vision to make marine biogeographic data freely available to the public [2]. SEAMAP is one of the participating network data nodes of OBIS (http://www.iobis.org), which in turn, is a member and data provider of the Global Biodiversity Information Facility (GBIF; http://www.gbif.org) [3]. Data are aggregated by specific marine taxa (SEAMAP), up to all marine biogeographic data (OBIS), and finally to global (including terrestrial) biogeographic data (GBIF). Compared to GBIF and OBIS, SEAMAP promotes the storage and publication of many more types of data (i.e., effort, animal behavior, etc.) while providing additional features and tools for both data providers and potential users interested in marine megavertebrates [4].

The SEAMAP project's objective is to compile any existing geo-referenced data for marine mammals, seabirds, and sea turtles that can be used to better understand the spatial and temporal patterns of species distribution and diversity in the global ocean, such as at-sea surveys (from a shipboard or aerial platform), land-based counts (from shore), satellite telemetry data (from tagged animals), and stranding data (from shore). These high quality data are standardized with a minimum set of fields including the taxon name, latitude/longitude position, and date/time for observations, begin/end dates and locations for effort data, and individual identification codes for satellite telemetry tag data [4]. Besides direct contributions, data can be delivered to SEAMAP from other data portals in an automated fashion. For instance, many of the sea turtle and telemetry datasets were fed into SEAMAP from the Satellite Tracking and Analysis Tool (STAT) available at SeaTurtle.org [5]. This tool enables direct consumption of the real-time ARGOS (Advanced Research and Global Observation Satellite) data. STAT users can freely sign up for their satellite tag data to be processed online with an option to upload and publish the data onto SEAMAP with a simple tick of a box.

Data collected for the SEAMAP project are publicly available on a web-based system (http://seamap.env.duke.edu/) that is intended for educators, students, managers, and researchers and provides advanced mapping tools, a variety of supplemental products (i.e., species profiles and photos), and tabular data summaries. The SEAMAP website also provides metadata for all datasets, including information on the data providers and collectors, description of techniques used to gather the data, survey effort details (when available), and methods to process the data for analysis. The interactive website features sophisticated querying and mapping capabilities for displaying specific data from multiple datasets across space and time. In addition, remotely sensed oceanographic data such as bathymetry and sea surface temperature can be viewed alongside observations. These environmental data are associated with observations synchronous in time and space, and available for download.

Currently, the SEAMAP database hosts over 2.3 million records with over 280 datasets from individuals, government agencies, non-profit organizations, and academic institutions. Ongoing dataset contributions from each of the three marine megafauna communities, both solicited and unsolicited, consistently increase numbers of observations and species holdings while expanding the temporal and geographic range of high quality data. However, it is apparent that temporal and geographic biases and gaps exist within the data holdings. A comprehensive gap analysis provides information that can facilitate prioritization in targeting new SEAMAP datasets to fill these gaps. In turn, results from the analysis provide direct feedback to management agencies and the research community for planning future surveys when a true gap in knowledge exists.

While the term “gap analysis” is used here to broadly refer to analyzing missing data, gap analysis has been referred to in the past as the approach of identifying conservation gaps by overlaying species distributions predicted from the environment with existing protected areas per the U.S. NBII Gap Analysis Program (http://gapanalysis.nbii.gov) [6]. This program's more specific gap analysis application with SEAMAP data would certainly add great conservation value, but is beyond the scope of this paper. Here, we more simply describe the gaps in the SEAMAP data holdings, bound by taxa, space, time, and environment.

## Results

As of February 12, 2009, SEAMAP has published over 2.2 million records from 240 datasets. About 20% of these include records with spatial errors (missing latitude or longitude coordinates or on land), missing observation dates, and observations that were not marine mammals, seabirds or sea turtles. The remaining published observations (1,839,510 records with 9,862,073 individual marine mammals, seabirds, and sea turtles observed) from 234 different datasets had all of the required data for the gap analysis. Points that fell on land were excluded, except for records from four shore-based surveys (988 records). The majority of records consisted of seabird observations from 67 datasets (1,607,041 records; 7,534,777 individuals; 230 different taxa). Combined observations of marine mammals from 181 datasets (154,485 records; 2,246,463 individuals; 116 different taxa) and sea turtles from 104 datasets (77,984 records; 80,833 individuals; 8 different taxa) made up less than half of the data and generally overlapped spatially with seabird distribution, although sea turtle observations were much lower in number in northern Europe. Since a large proportion of observation data (over 87% of records, and over 76% of animals) consisted of seabirds, temporal and spatial distribution results were broken down by taxa rather than lumping all three taxa together.

Overall, most datasets and records were gathered from shipboard surveys (n = 115; 1,478,671 records), followed by aerial surveys (n = 66; 251,107 records), tag data (n = 49; 100,527 records), and shore-based observations (n = 4; 9,205 records). The majority of marine mammals and seabird datasets and records came from shipboard observations (63% and 86% of records, respectively), but sea turtle data mostly came from tags (86% of records). When comparing the number of individuals (number of records * group size) from aerial, shipboard, and shore surveys, the relationship between the number of records and animals obtained varied by platform and taxa. Shore surveys gave the greatest group sizes for marine mammals (mean = 28) and seabirds (mean = 1,161). After shore surveys, shipboard surveys had the next highest group size for marine mammals (mean = 16) though only slightly higher than data gathered from aerial surveys (mean = 14). For seabirds, aerial survey datasets had a slightly higher mean group size over the shipboard datasets (mean = 6 and 4, respectively). Sea turtle mean group sizes did not differ among platforms (mean = 1).

As of the February 2009 cut-off, three shore surveys for marine mammals included data from whale strandings, seal haulouts, and sea otter sightings while the one shore-based seabird dataset included data from seabird colonies. Although SEAMAP currently displays two shore-based datasets on sea turtles (i.e., sea turtle nesting sites), no shore-based sea turtle data was included in the gap analysis because data were unavailable for public download at the time of this analysis.

Trackline data published on SEAMAP was associated with the majority (>78%) of datasets examined in this analysis. Since only a few survey trackline datasets flagged records as either “on” or “off” effort, it was assumed that all data were “on” or qualified as a period or track length where observations were actively being sought and recorded when a dataset did not have flags. A total of 184 observation datasets had associated trackline data (1,338,771 records within Food and Agriculture Organization [FAO] global statistical fishing areas [7]), mainly from datasets collected by shipboard surveys (n = 85) and aerial surveys (n = 54), followed by satellite tag data (n = 45). Globally, 3,956,372 km of tracklines are represented on SEAMAP. These tracklines, or polylines, represent the shortest distance connecting points reported as positions within a survey track or within a satellite tag path. However, the total length of effort calculated per region may be an underestimate since survey effort and a tagged animal's path are unlikely to be a straight line between recorded locations.

### Temporal distribution

**Figure 1:**
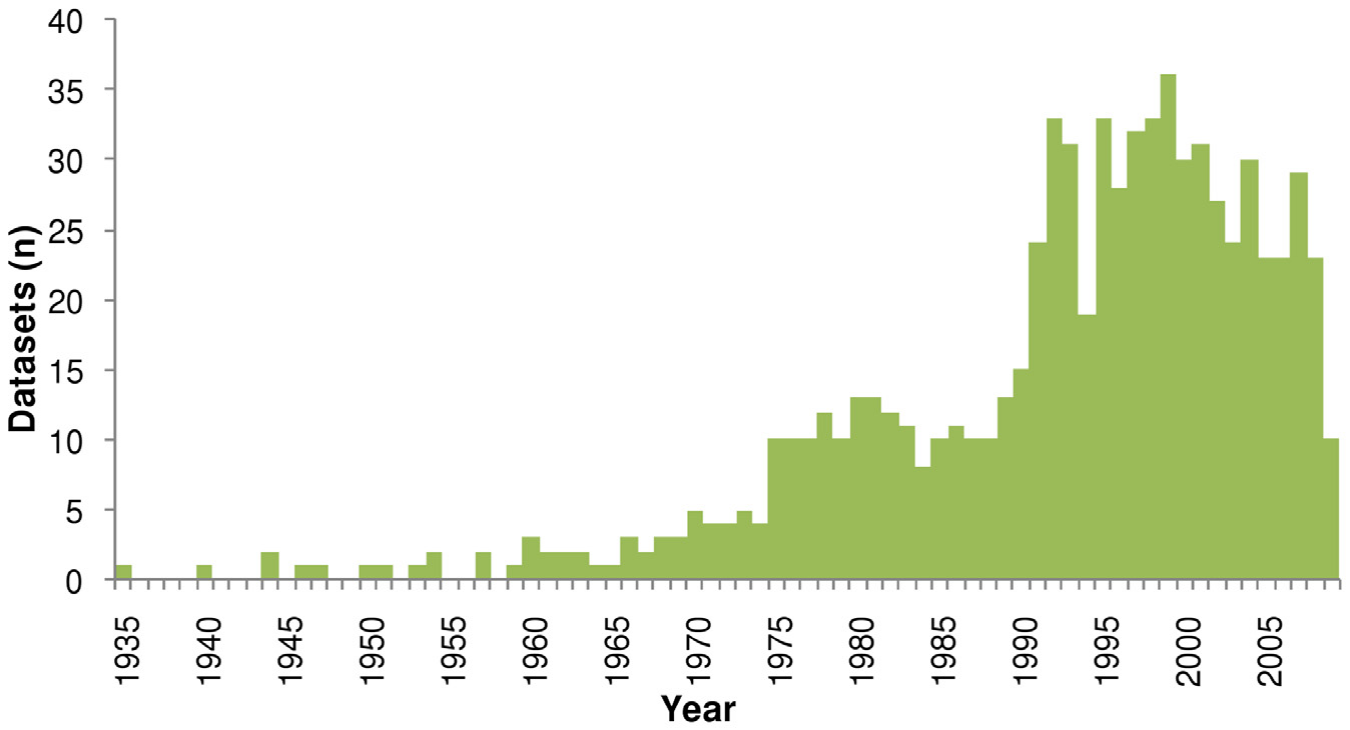
The number of datasets published on SEAMAP containing data records for each year of observations.

**Figure 2:**
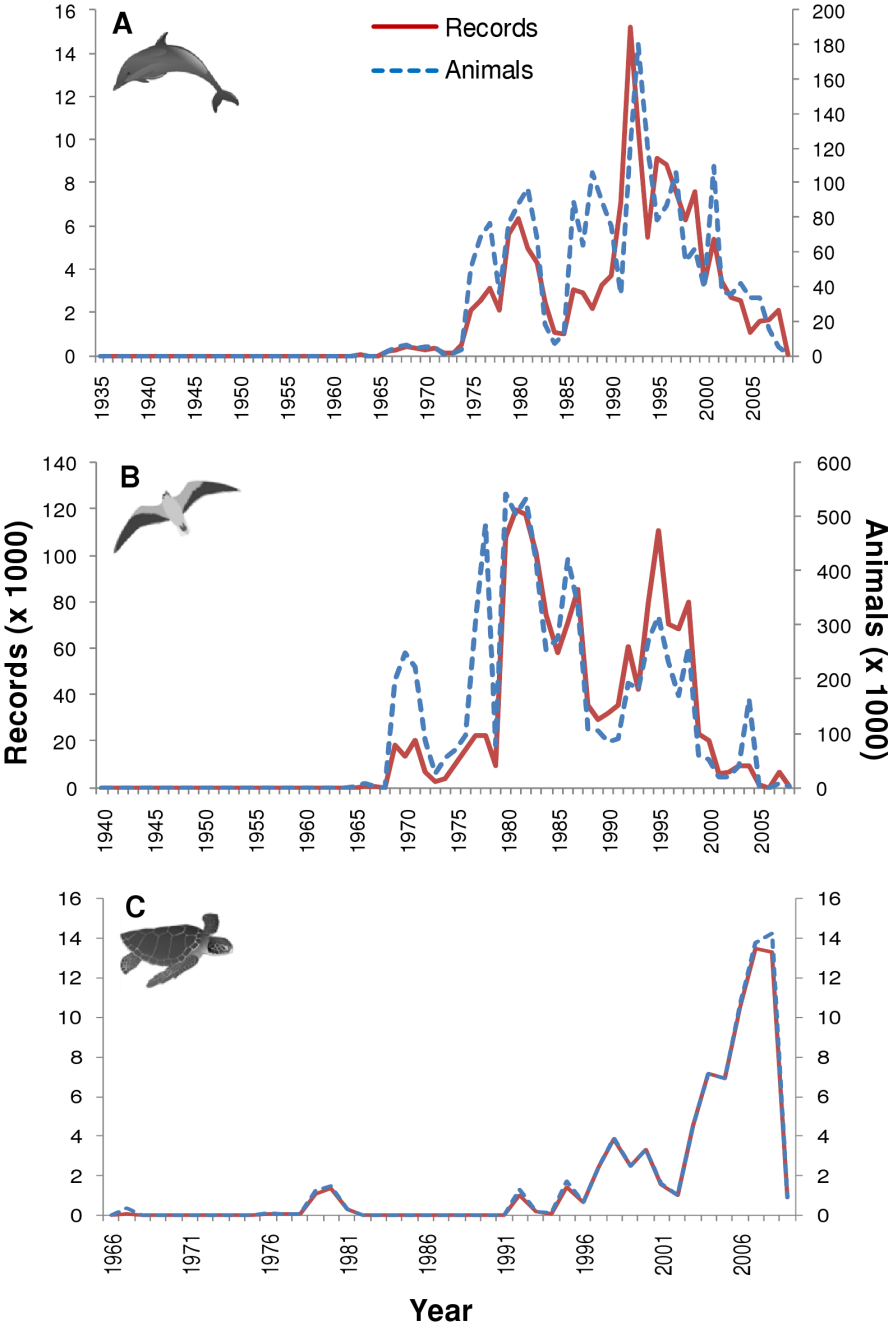
The number of records and animals published on SEAMAP each year. Number of records (solid red line) and animals (dashed blue line) for: (A) marine mammals; (B) seabirds; and (C) sea turtles. (Marine mammal, seabird, and sea turtle icon credit: Tracey Saxby, IAN Image Library, ian.umces.edu/imagelibrary/).

For the 234 datasets published on SEAMAP and included in this analysis, most contained data observed during the late 1990s, with the maximum number of datasets contributing to any one year occurring in 1999 (n = 36; Figure 1). Marine mammal data published on SEAMAP ranged from 1935–2009, sea turtle data ranged from 1966–2009, and seabird data ranged from 1940–2008 (Figure 2). For marine mammals, the peak number of records was in 1992 (n = 15,219) and the peak in observed animals was in 1993 (n = 179,720; Figure 2a). For seabirds, SEAMAP had the highest number of records occurring in 1981 (n = 119,573) and the highest number of seabirds observed in 1980 (n = 541,156; Figure 2b). Finally, sea turtle records and individuals were extremely low for most years within the time range with a drastic peak in records during 2007 (n = 13,447) and animals in 2008 (n = 13,273; Figure 2c). This recent jump in the number of records and observations was due to five datasets, two of which were from satellite tags contributing the majority of the data in 2007 and 2008 (12,375 and 12,149 records respectively).

**Figure 3:**
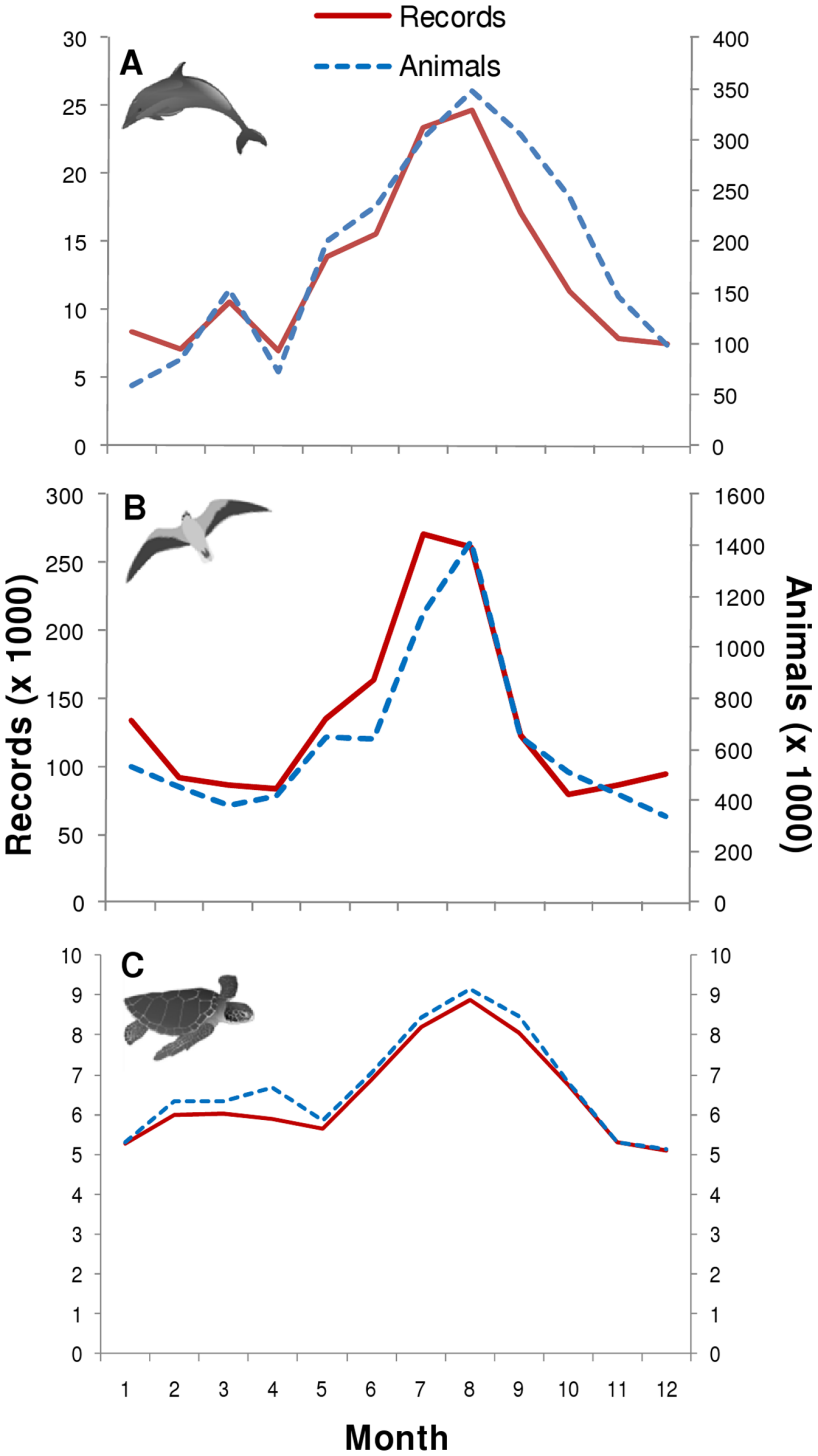
The number of records and animals published on SEAMAP each month. Number of records (solid red line) and animals (dashed blue line) for: (A) marine mammals; (B) seabirds; and (C) sea turtles. (Marine mammal, seabird, and sea turtle icon credit: Tracey Saxby, IAN Image Library, ian.umces.edu/imagelibrary/).

Recorded observations and number of individuals were highest in the summer for all three major taxa (Figure 3). This also coincided with the length of tracklines published on SEAMAP per season with summer having the greatest coverage, followed by fall, spring, and winter. When observations were broken down by taxa, the number of records and animals were next highest for marine mammals and sea turtles in the fall, followed by spring and then winter (Figures 3a and 3c). For seabirds, however, high records were found in winter, followed by spring and then fall while the number for animals observed was higher in the fall, followed by spring and then winter (Figure 3b). The higher number of seabird records in the winter was mostly due to the increased number of aerial surveys compared to fall while the higher number of seabirds observed in the fall was mostly due to more shipboard surveys than in the winter. July and August were the peak summer months while December and January had some of the lowest numbers. However, April had the least number of records for marine mammals and seabirds.

### Spatial distribution

**Figure 4:**
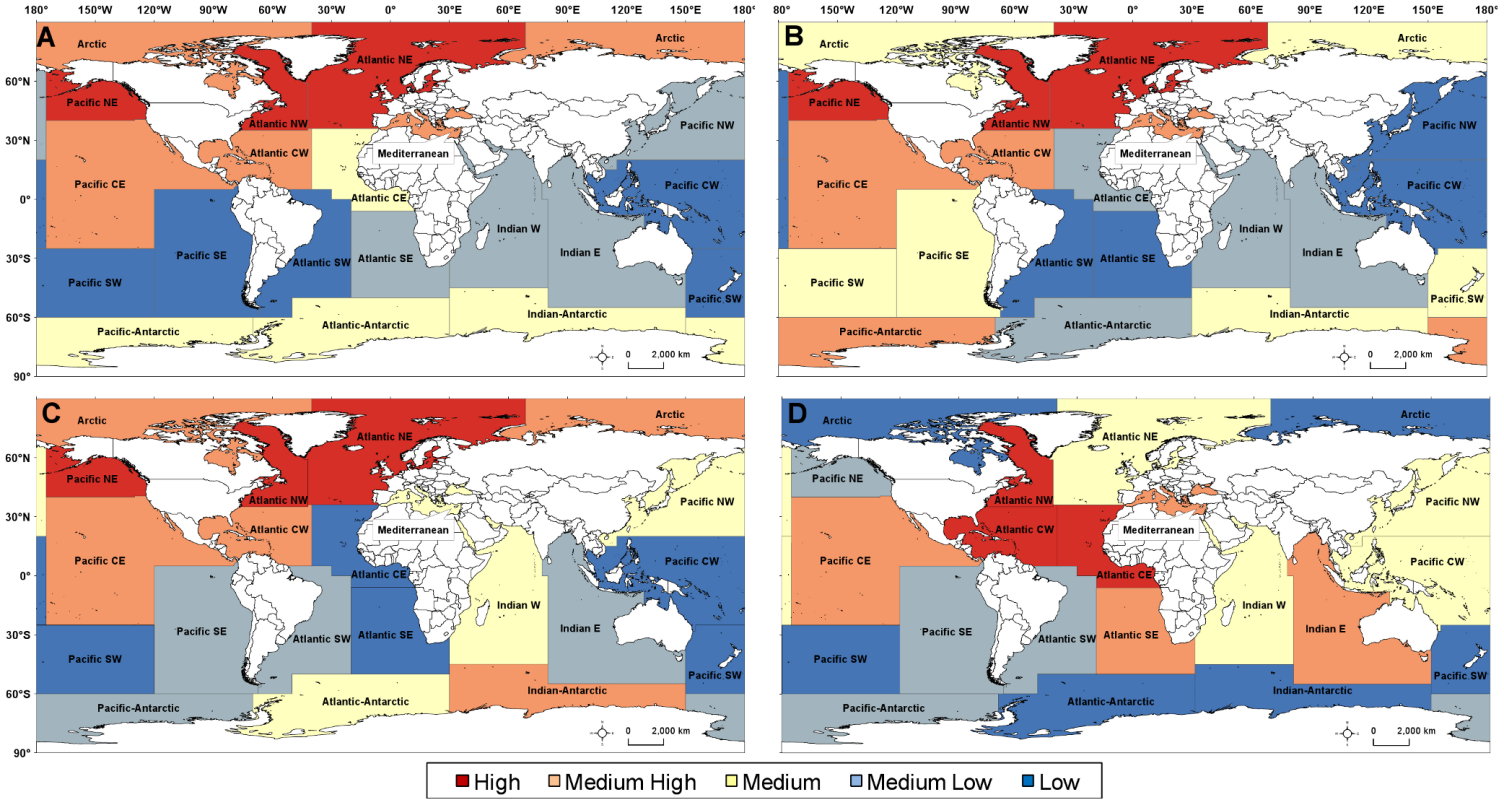
Relative abundance of records per square km published on SEAMAP in FAO statistical fishing areas. Abundance for: (A) all marine mammals, seabirds, and sea turtles; (B) marine mammals; (C) seabirds; and (D) sea turtles. Each color represents one quintile (hot to cool colors: red, orange, yellow, light blue, and dark blue), containing the number of records that fell into the >79^th^ (high), 79-60^th^ (medium-high), 59-40^th^ (medium), 39-20^th^ (medium-low), and <20^th^ (low) percentiles, respectively. See Table 1 for FAO global statistical fishing area codes and regions.

Aggregated SEAMAP data revealed large gaps in the Southern Hemisphere, especially in the Pacific and Atlantic Oceans, while there was a high concentration of records in the Pacific northeast (NE), and northern Atlantic (Figure 4a; Table 1). The highest number of datasets were found to contribute data in the Pacific NE (n = 72), followed closely by the Atlantic central-west (CW; n = 69); regions with the least number of datasets (n = 3 for each region) were the Arctic, Atlantic southwest (SW), Atlantic-Antarctic, and Pacific-Antarctic. Marine mammal records were present in every region, but were particularly low in the Pacific CW and northwest (NW) as well as the Atlantic SW and southeast (SE; Figure 4b). High concentrations of marine mammal records and animals were in the Pacific central east (CE), northern Atlantic, and Mediterranean. Seabird records and number of animals were low in most of the southern regions, with major gaps in the Atlantic CE and SE, Pacific SW and SE, with no records found in the Pacific CW (Figure 4c). Compared to the other taxa, a higher proportion of seabird records were found in the Pacific NE, Arctic, and Indian-Antarctic. Sea turtles, however, had high numbers of records from the Atlantic CW and CE regions, but gaps in the southern Pacific, Arctic and Antarctic regions (Figure 4d). Absence of sea turtle observations near the poles is expected as this ectothermic taxa requires warmer habitats.

**Table 1:** Regional summary of all marine mammal, seabird, and sea turtle observations data published on SEAMAP.

Region (FAO Code)	Records (n)	Animals (n)	Records per million km^2^
Arctic (18)	8,364	78,263	1,145.75
Atlantic CE (34)	9,906	15,409	707.57
Atlantic CW (31)	58,193	371,641	3,958.71
Atlantic NE (27)	1,146,130	3,585,443	67,818.34
Atlantic NW (21)	250,257	2,844,695	48,126.35
Atlantic SE (47)	2,025	2,601	108.87
Atlantic SW (41)	1,454	8,100	82.61
Atlantic-Antarctic (48)	6,679	54,928	543.01
Indian E (57)	5,488	10,353	184.16
Indian W (51)	4,922	42,663	162.98
Indian-Antarctic (58)	14,435	115,103	1,145.63
Mediterranean (37)	5,142	170,739	1,714.00
Pacific CE (77)	137,924	1,611,324	2,820.53
Pacific CW (71)	2,599	5,479	78.28
Pacific NE (67)	173,318	793,335	23,109.07
Pacific NW (61)	3,258	3,939	158.93
Pacific SE (87)	2,972	3,309	99.07
Pacific SW (81)	3,198	134,807	112.61
Pacific-Antarctic (88)	3,246	9,942	312.12

**Figure 5:**
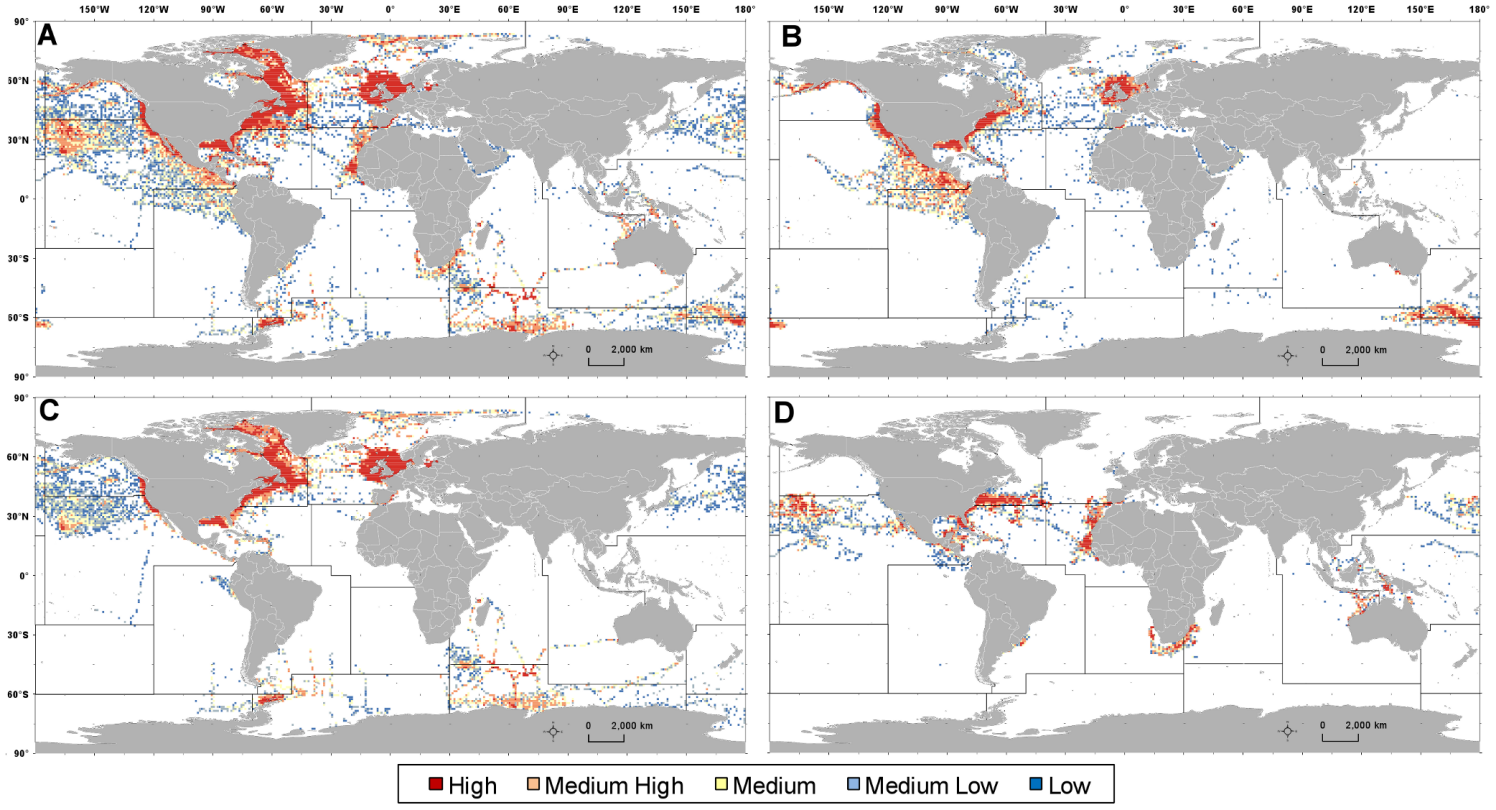
Relative abundance of records per square degree published on SEAMAP in 1 degree square cells. Abundance for: (A) all marine mammals, seabirds, and sea turtles; (B) marine mammals; (C) seabirds; and (D) sea turtles. Each color represents one quintile (hot to cool colors: red, orange, yellow, light blue, and dark blue), containing the number of records that fell into the >79^th^ (high), 79-60^th^ (medium-high), 59-40^th^ (medium), 39-20^th^ (medium-low), and <20th (low) percentiles, respectively.

Within FAO regions, high densities of records for all taxa were shown to be particularly biased towards the coastal areas with decreasing densities moving further offshore (Figure 5). Coastal biases were particularly apparent for marine mammals and seabirds around the United States (US) and United Kingdom (Figures 5b and 5c). Less clustered areas (low record densities) further offshore were a result of satellite tag datasets that tracked individual animal movements across large regions such as seabirds in the Pacific NE and CE (Figure 5c) and sea turtles in the Pacific CW (Figures 5d).

**Figure 6:**
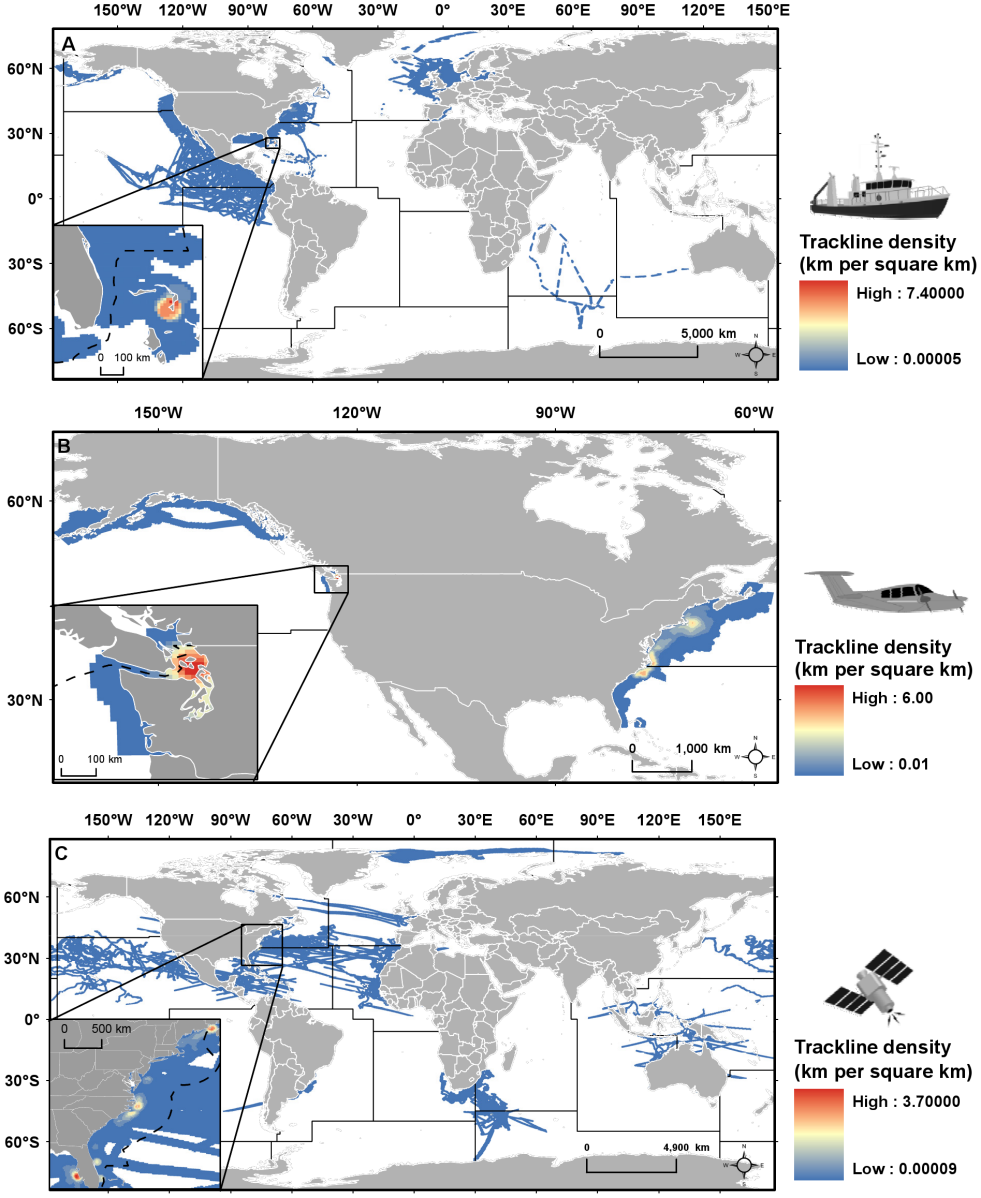
Relative density of tracklines published on SEAMAP for global FAO regions. Trackline data collected by: (A) shipboard surveys; (B) aerial surveys; and (C) satellite telemetry tag. Insets highlight the highest density per platform; FAO region borders are solid lines and US EEZ borders are dashed lines. (Ship, plane, and satellite icon credit: Tracey Saxby, IAN Image Library, ian.umces.edu/imagelibrary/).

The relative distribution of tracklines among and within regions sensibly coincides with observational records (Figures 5 and 6). Most of the published tracklines were concentrated in the north and central Atlantic regions (highest km of tracklines was found in the Atlantic NW region). SEAMAP did not have any published datasets with tracklines in the Pacific-Antarctic (Table 2). The area covered in most regions was minimal and the presence of effort within an area was less than 50% for all regions (Table 2), with most tracklines concentrated near the coast. The amount of areal coverage calculated per region is likely an overestimate since the buffer around the trackline was larger than what is typical (survey/animal swaths are usually <100 km of the ship, plane, satellite telemetry tag path).

**Table 2:** Regional summary of tag, shipboard survey, and aerial survey trackline data published on SEAMAP.

Region (FAO Code)	Shipboard survey (km)	Aerial survey (km)	Tag (km)	Total (km)	Trackline length (km) per million km^2^	Trackline coverage (% area)
Arctic (18)	0.00	0.00	2,166.32	2,166.32	296.76	1.50
Atlantic CE (34)	143.55	0.00	187,415.37	187,558.93	13,397.07	33.02
Atlantic CW (31)	173,706.37	125,772.93	447,505.59	746,984.89	50,815.30	28.14
Atlantic NE (27)	508,823.86	0.00	105,519.58	614,343.44	36,351.68	30.55
Atlantic NW (21)	265,028.59	516,315.97	246,527.56	1,027,872.11	197,667.71	35.17
Atlantic SE (47)	0.00	0.00	43,382.43	43,382.43	2,332.39	6.80
Atlantic SW (41)	0.00	0.00	6,972.68	6,972.68	396.17	2.03
Atlantic-Antarctic (48)	0.00	0.00	395.23	395.23	32.13	0.14
Indian E (57)	1,762.12	0.00	60,051.27	61,813.39	2,074.27	6.35
Indian W (51)	7,678.14	0.00	81,864.49	89,542.63	2,964.99	8.45
Indian-Antarctic (58)	4,679.41	0.00	71,189.58	75,868.99	6,021.35	15.72
Mediterranean (37)	59,091.06	0.00	1,168.29	60,259.36	20,086.45	5.91
Pacific CE (77)	372,602.97	0.00	215,460.17	588,063.14	12,025.83	36.28
Pacific CW (71)	0.00	0.00	72,908.16	72,908.16	2,196.03	11.63
Pacific NE (67)	36,447.53	130,635.27	6,547.17	173,629.97	23,150.66	14.32
Pacific NW (61)	521.65	0.00	48,504.91	49,026.57	2,391.54	12.67
Pacific SE (87)	150,428.73	0.00	3,622.74	154,051.47	5,135.05	19.04
Pacific SW (81)	0.00	0.00	1,532.75	1,532.75	53.97	0.35
Pacific-Antarctic (88)	0.00	0.00	0.00	0.00	0.00	0.00

When broken down by platform, densities of shipboard survey tracks were the highest at around 7.4 km per square km, found near Great Abaco Island, Bahamas (Figure 6a) where high numbers of sea turtles (loggerhead *Caretta caretta* being the most abundant species) and cetaceans (bottlenose dolphin *Tursiops truncatus* being the most abundant species) were observed. Trackline density for aerial surveys was highest around 7 km per square km, found in Puget Sound, Washington, US (Figure 6b), where high numbers of seabirds (northern fulmar *Fulmarus glacialis* and common murre *Uria aalge* being the most abundant species) were observed. Tracklines for satellite tag data were the least dense out of the three platforms, with a maximum of around 3.7 km per square km found near St. Petersburg, Florida, US (Figure 6c) where high numbers of sea turtles (loggerheads being the most abundant species) were tagged and tracked. Other hotspots for satellite tag trackline density appeared near Grand Manan Island, New Brunswick, Canada where many harbor porpoises (*Phocoena phocoena*) were tracked and near Pamlico Sound, North Carolina, US where loggerhead sea turtles were the most abundant species tracked (Figure 6c).

### Oceanographic coverage

It was not possible to associate environmental variables with many of the dates and locations for observations because the availability of such variables was restricted to more recent years (Table 3) and limited by cloud cover. About 92% of observations were associated with bathymetry because temporal restrictions did not apply, though data were lacking in coastal areas. Sea surface temperature (SST) data were available for over 20 years and could be associated with about 57% of observations in time and space. For sea surface height (SSH) and chlorophyll (CHL) data, only about a quarter or less of the observations were matched in time and space mostly due to the temporal restrictions of the environmental data. Besides pre-dating the availability of satellite coverage and cloud cover, environmental data may also be missing due to calculation error associated with coastal cells (and equatorial cells for SSH) or satellite instrument malfunctions. For SST and CHL, the trends of using weekly/8 day, monthly, and yearly values for each taxa were the same (i.e., yearly, monthly, and weekly SST values were highest for sea turtles and lowest for seabirds) and, similarly, weekly values of SSH followed the same relationship with taxa. Therefore, only monthly values were presented here to show seasonal averages, modes, and ranges (see Tables 4 and 5).

**Table 3:** Environmental variables used to assess marine mammal, seabird, and sea turtle observations published on SEAMAP.

Variable	Source	Availability	Cell size	Averaging	Available data
Bathymetry (Depth)	S2004 (compilation of GEBCO [52] and Smith/Sandwell [53])	All Years	1 arc-minute	None	n = 92%
Sea Surface Temperature (SST)	AVHRR v5 Sea Surface Temperature [54]	1985–2006	0.04 degree	Yearly, Monthly, Weekly	Year: 57% Month: 57% Week: 57%
Sea Surface Height (SSH)	Aviso Maps of Absolute Dynamic Topography (MADT; [55])	October 14, 1992–May 23, 2007	0.25 degree	Weekly	Month: 26% Week: 26%
Chlorophyll (CHL)	SeaWiFS Sea Surface Chlorophyll [56]	September 4, 1997–June 1, 2006	0.08 degree	Yearly, Monthly, 8 days	Year: 13% Month: 8% 8 day: 6%

**Table 4:** Average value of environmental variables sampled by taxa and by platform.

		Depth (m)	Monthly SST (°C)	Monthly SSH (cm)	Monthly CHL (mg/m^3^)
Taxa					
	Marine mammals	764	14.1	129.8	2.04
	Seabirds	311	10.0	122.6	1.30
	Sea turtles	2,550	21.4	179.9	0.29
Platform					
	Shipboard	329	11.7	116.0	1.24
	Aerial	321	7.3	154.7	2.53
	Tag	2,448	15.3	179.7	0.73
	Shore	17	6.9	115.8	5.99

**Table 5:** The mode and range (in parenthesis) of environmental variables sampled by observations of marine mammals, seabirds, and sea turtles, separated by platform.

Taxa	Platform	Depth (m)	Monthly SST (degrees C)	Monthly SSH (cm)	Monthly CHL (mg/m^3^)
Marine mammals					
	Shipboard	1 (1–8,059)	25.4 (−2.9–35.18)	162.2 (−16.5–246.7)	1.8 (0.03–37.15)
	Aerial	12 (1–4,575)	13.4 (−2.9–29.5)	149.4 (96.5–199.9)	2.3 (0.09–21.38)
	Tag	1 (1–5,139)	6.4 (−2.9–20.8)	127.0 (80.8–139.4)	2.3 (0.36–15.14)
	Shore	1 (1–149)	5.8 (−2.9–18.2)	118.5 (104.2–122.5)	4.5 (1.78–44.16)
Seabirds					
	Shipboard	53 (1–9,294)	−2.9 (−2.9–31.2)	99.6 (119.3–261.8)	1.5 (0.03–25.41)
	Aerial	1 (1–4,473)	−2.9 (−2.9–28.7)	157.0 (139.8–182.7)	2.6 (0.35–30.20)
	Tag	15 (1–7,761)	23.3 (−2.9–27.3)	224.6 (119.3–261.8)	0.3 (0.03–14.62)
Sea turtles					
	Shipboard	1 (1–5,731)	30.7 (3.4–31.7)	184.8 (91.0–212.2)	0.2 (0.06–5.37)
	Aerial	1 (1–4,596)	24.2 (−2.6–29.5)	127.0 (98.8–166.8)	0.4 (0.14–7.59)
	Tag	5,614 (2–6,518)	17.8 (−1.7–31.0)	193.6 (107.9–314.8)	0.1 (0.02–8.41)

The distribution of bathymetry, SST, CHL, and SSH by taxa and by platform all deviated significantly from normal (Shapiro-Wilk test, *p*<0.001). In addition, most environmental layers by taxa and by platform significantly deviated from unimodality (*p*<0.001). Exceptions included bathymetry sampled by sea turtle sightings gathered by shipboard survey and by satellite tag, yearly SSH sampled by sea turtle sightings gathered by satellite tag, and SSH sampled by seabird sightings gathered by shipboard survey (*p*>0.001).

**Figure 7:**
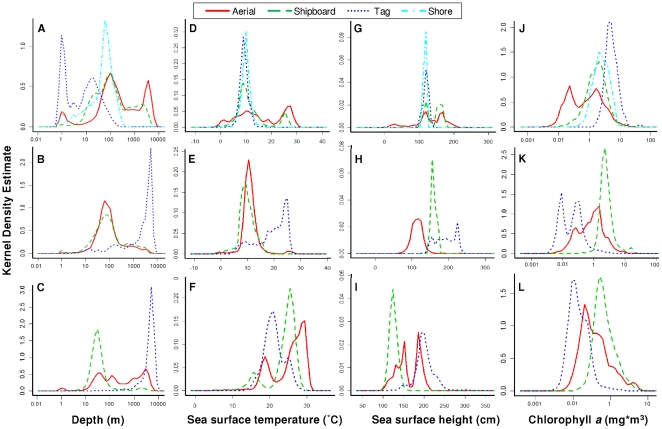
Kernel density estimates of environmental variables. Depth (A–C), sea surface temperature (D–F), sea surface height (G–I), and chlorophyll *a* (J–L) density estimates for marine mammals (A, D, G, J), seabirds (B, E, H, K), and sea turtles (C, F, I, L). Observations collected by aerial surveys (solid red line), shipboard surveys (dark green long dashed line), tag (dark blue short dashed line), and shore (light blue dashed and dotted line, only for marine mammals) were used to sample environmental variables. Note that depth and chlorophyll *a* graphs are on a log scale.

Depth values associated with observations ranged from 1 to 9,294 m, but were heavily skewed towards shallow waters (<350 m; median = 82 m; mode = 53 m). Average depths varied significantly with taxa and platform (Kruskal-Wallis test, *p*<0.01; Table 4). Sea turtles were observed over the deepest waters, followed by marine mammals, and then seabirds; datasets gathered by tag were over deeper waters than ships, followed by aerial surveys and shore surveys (Table 4). For marine mammals, the number of records for all four platforms peaked in shallow waters; the most frequent bathymetry for observations collected by ship, tag, and shore was at 1 m while data gathered by aerial surveys were mostly at 12 m (Table 5). Kernel density distributions for marine mammal depths differed greatly among platforms (Figure 7a). For seabirds, all platforms had high numbers of observations from shallow waters, while satellite tag data also had an abundance of data over depths around 5,500 m (Figure 7b). Sea turtles were also mostly observed in shallow waters, although the satellite tags allowed for a greater number of observations over deep water, with most data over waters around 5,600 m deep (Figure 7c, Table 5). Not including the shore-based datasets, data gathered by aerial surveys had the smallest range compared to other platforms, with a range of only about 4,600 m (Table 5).

Monthly SST values associated with observations ranged from −2.92 to 35.17 degrees Celsius (°C), with most observations at −2.9°C although many temperatures were around the median (11.10°C). Like bathymetry, average SST (weekly, monthly, and yearly) varied significantly with taxa and platform (Kruskal-Wallis test, *p*<0.01; Table 4). Sea turtles were observed in higher SSTs, followed by marine mammals, and then seabirds; datasets gathered by tag were in warmer waters than shipboard surveys, followed by aerial surveys, and then shore surveys (Table 4). For marine mammals, most shipboard observations were in the warmest waters, followed by planes, tags and then shore observations (Table 5). Kernel density distributions for marine mammal SSTs varied greatly among the different platforms (Figure 7d). For seabirds, aerial and shipboard surveys had high numbers of observations around 7 and 10°C, respectively, while satellite tag data included an abundance of data recorded in warmer waters (Figure 7e, Table 5). Sea turtles were primarily observed in warm waters, with modes between 17–31°C for each platform (Figure 7f, Table 5).

Monthly SSH values associated with observations ranged from −16.5 to 314.8 cm (median = 119.1 cm; mode = 157 cm). SSH (daily and monthly) varied significantly with taxa and platform (Kruskal-Wallis test, *p*<0.01). Sea turtles were observed in higher SSHs, followed by marine mammals, and then seabirds; datasets gathered by tag were higher in SSH than aerial surveys, followed by shipboard, and then shore-based surveys (Table 4). For marine mammals, the number of records for all four platforms peaked in waters around 120 cm, with shipboard and aerial observations peaking again near 160 cm (Figure 7g; Table 5). For seabirds, all platforms peaked at different SSHs, with shipboard observations in lower waters than aerial observations, followed by satellite tags (Figure 7h, Table 5). For sea turtles, all platforms also peaked at different SSHs, with shipboard survey data having peaks slightly overlapping with both aerial and tag data (Figure 7i). Compared with other environmental variables, SSH modes seem to be most divided among platforms per taxa.

Monthly CHL values associated with observations ranged from 0.01 to 44.15 mg/m^3^ (median = 0.86 mg/m^3^; mode = 1.50 mg/m^3^). CHL (weekly and monthly) varied significantly with taxa and platform (Kruskal-Wallis test, *p*<0.01). Marine mammals were observed in highest average CHLs, followed by seabirds, and then sea turtles; datasets gathered from shore were higher in average CHL than aerial surveys, followed by shipboard surveys, and then tag data (Table 4). For all taxa and all platforms, the number of records peaked in waters of low CHL concentration (Figures 7j–l, Table 5). Shore surveys yielded the widest range of data for CHL, while sea turtles observed by shipboard surveys had the smallest range along with relatively low CHL concentrations (Table 5).

### Identifying potential datasets

A total of 636 references were identified with many high quality datasets within all regions, including regions in which the SEAMAP project currently holds little or no data (Text S1). There were no regions that showed an absence of seabird, marine mammal, or sea turtle observations collected from either dedicated surveys or satellite telemetry. Initially, the number of potential datasets identified grew exponentially with time spent on searching with the use of literature, networking, and websites due to the snowball sampling effect [8]; the collaborative nature of many studies and information in the bibliography and acknowledgments led to information on other organizations with similar datasets. The first round of data requests, primarily via e-mail, to acquire many of the identified datasets showed significant potential. As the search and review of literature progresses, there usually comes a point at which the rate of information gained with respect to effort decreases [9]. For the scope of this gap analysis, the timeframe allocated to searching for datasets had not yet reached this point of diminishing returns. However, it was apparent that older datasets (early 1980s and before) did have diminishing returns in that the effort to find current contacts, appropriate permissions, and acquire data in readily available formats increased with the age of the dataset.

Quantitative results of the quintile subtraction analysis showed that the largest gaps in SEAMAP data holdings compared to what was missing in the literature were in the Atlantic SW for all taxa combined as well as for marine mammals in particular (Table 6). In addition, the amount of marine mammal data was low for the Pacific NW and seabird data holdings showed the largest difference between references and records within the Atlantic Antarctic and Pacific Antarctic. In contrast, SEAMAP had relatively good coverage (small difference between the relative number of references and published records) within the Atlantic NE, where there were high numbers of records for all taxa combined, and the Indian Antarctic, where there was a medium amount of records for all taxa combined (Figure 4; Table 6). The amount of sea turtle data on SEAMAP was also greater in relation to the number of literature references in the Atlantic NW, where the number of records was relatively high. It is important to note that these results only give an estimate of regional distributions of data within the literature for datasets not already published on SEAMAP and the literature search was not comprehensive.

**Table 6:** Regional results from the quintile subtraction analysis for all taxa; higher numbers (in bold) represent larger data gaps in SEAMAP relative to the literature search.

Region (FAO Code)	All taxa	Marine mammal	Seabird	Sea turtle
Arctic (18)	0	1	1	0
Atlantic CE (34)	−1	1	0	−4
Atlantic CW (31)	0	0	0	0
Atlantic NE (27)	−1	−1	−2	1
Atlantic NW (21)	0	0	−2	0
Atlantic SE (47)	0	1	0	0
Atlantic SW (41)	**2**	**2**	1	1
Atlantic-Antarctic (48)	−1	0	**2**	0
Indian E (57)	−1	0	−1	−1
Indian W (51)	0	1	−1	1
Indian-Antarctic (58)	−2	−2	−1	0
Mediterranean (37)	1	1	−2	1
Pacific CE (77)	−1	−1	0	−1
Pacific CW (71)	0	0	0	1
Pacific NE (67)	0	0	0	1
Pacific NW (61)	1	**3**	−1	−2
Pacific SE (87)	−1	−2	0	0
Pacific SW (81)	0	−2	1	1
Pacific-Antarctic (88)	−1	−2	**2**	−1

The majority of datasets published on SEAMAP were contributed by government groups (n = 150), followed by non-government groups (n = 50) and universities (n = 34). When breaking the number of records down by taxa, government groups provide the largest number for seabirds and marine mammals while non-government groups provide the most data for sea turtles. For the four platforms, government groups provide the most data by shipboard and aerial surveys, non-government groups give the most satellite tag data, and universities provide the most shore-based data. The number of datasets were mostly from US-based organizations (n = 170). When broken down by platform, however, more shipboard and satellite tag data (records and number of animals sighted) were from non-US contributors. US-based organizations provided more records and animal observations for marine mammals and sea turtles but not for seabirds.

Results from the preliminary literature search showed that there were numerous potential contacts within the government, non-government, and university groups that have collected data suitable for SEAMAP, including data to fill many temporal and spatial gaps (Text S1). When targeting specific groups and searching within these sectors, internet searches and conferences proceedings were more effective than primary literature databases as websites and conference presentations were more current and often disseminated information through gray literature. Limits for online searches include the existence of a website, the presence of keywords to be retrieved by search engines, and enough available information on research projects and key contacts to be considered. The most common limitation for obtaining the data presented at recent conferences was that the data were still being worked on for peer review publication or other projects and researchers were reluctant to make data available to the public beforehand.

### Comparing portal coverage with example species

As of January 25, 2010, the US Fish and Wildlife Service listed 28 marine mammals, 19 seabirds, and 6 sea turtles as endangered or threatened based on the level of protection needed to reduce the danger of the population's extinction [10]. Of the 53 animals listed, the SEAMAP project currently has no records for 9 marine mammals (Table 7) and no records for 12 seabirds (Table 8), but does have at least one record of each of the sea turtle species listed (Table 9). With the exception of the bowhead whale (*Balaena mysticetus*), the listed marine mammals and seabirds not included in SEAMAP are known to be endemic to relatively small geographic ranges. Other listed animals with low representation (<10 records) in SEAMAP, and a more wide habitat range, include the endangered North Pacific right whale (*Eubalaena japonica*) [11] and endangered beluga (*Delphinapterus leucas*) [12]. Preliminary searches using literature databases showed that data from recent research (within the last 25 years) were published for the 15 endangered and threatened marine mammals with little to no representation on SEAMAP.

**Table 7:** The number of marine mammal records and animals published on SEAMAP that are protected under the US Endangered Species Act, based on the US Fish and Wildlife listings (E = endangered, T = threatened, E/T = populations are endangered and threatened; [10]).

Status	Species	Common Name	Records (n)	Animals (n)
T	*Arctocephalus townsendi*	Guadalupe fur seal	1	1
E	*Balaena mysticetus*	Bowhead whale	0	0
E	*Balaenoptera borealis*	Sei whale	159	446
E	*B. musculus*	Blue whale	8,539	18,116
*E	*B. m. brevicauda*	Pygmy blue whale	1	1
E	*B. physalus*	Fin whale	6,319	14,294
E	*Delphinapterus leucas*	Beluga	7	37
E	*Dugong dugon*	Dugong	3	4
T	*Enhydra lutris*	Sea otter	2,042	13,667
*T	*E. l. nereis*	Southern sea otter	14	17
E	*Eschrichtius robustus*	Gray whale	1,608	3,688
E	*Eubalaena australis*	Southern right whale	13	42
E	*E. glacialis*	Northern right whale	757	1,633
E	*E. japonica*	North Pacific right whale	1	2
E/T	*Eumetopias jubatus*	Steller's sea lion	257	543
E	*Lipotes vexillifer*	Chinese river dolphin	0	0
E	*Lontra felina*	Marine otter	0	0
E	*L. provocax*	Southern river otter	34	54
E	*Megaptera novaeangliae*	Humpback whale	12,040	24,045
E	*Monachus monachus*	Mediterranean monk seal	0	0
E	*M. schauinslandi*	Hawaiian monk seal	0	0
E	*Orcinus orca*	Killer whale	614	2,826
E	*Phoca hispida saimensis*	Saimaa seal	0	0
E	*Phocoena sinus*	Vaquita	26	43
E	*Physeter macrocephalus*	Sperm whale	2,075	7,188
E	*Platanista minor*	Indus river dolphin	0	0
E	*Trichechus inunguis*	Amazon manatee	0	0
E	*T. manatus*	Caribbean manatee	9	11
T	*T. senegalensis*	West African manatee	0	0
T	*Ursus maritimus*	Polar bear	25	33

*SEAMAP holds records for these subspecies.

**Table 8:** The number of seabird records and animals published on SEAMAP that are protected under the US Endangered Species Act, based on the US Fish and Wildlife listings (E = endangered, T = threatened, E/T = populations are endangered and threatened; [10]).

Status	Species	Common Name	Records (n)	Animals (n)
T	*Brachyramphus marmoratus*	Marbled murrelet	1,677	3,236
E	*Fregata andrewsi*	Andrew's frigatebird	0	0
E	*Larus audouinii*	Audouin's gull	117	513
E	*L. relictus*	Relict gull	0	0
E	*Phoebastria albatrus*	Short-tailed albatross	1,447	2,393
E	*Pterodroma aterrima*	Mascarene black petrel	0	0
E	*P. axillaris*	Chatham Island petrel	0	0
E	*P. cahow*	Bermuda petrel	0	0
E	*P. macgillivrayi*	Fiji petrel	0	0
E	*P. magentae*	Magenta petrel	0	0
T	*P. phaeopygia*	Galapagos petrel	2	4
E	*P. p. sandwichensis*	Hawaiian dark-rumped petrel	0	0
T	*Puffinus auricularis*	Townsend's shearwater	1	13
T	*P. auricularis newelli*	Newell's shearwater	0	0
T	*P. heinrothi*	Heinroth's shearwater	0	0
E	*Spheniscus mendiculus*	Galapagos penguin	0	0
E	*Sterna antillarum*	Least tern	48	453
E	*S. a. browni*	California least tern	0	0
E/T	*S. dougallii dougallii*	Roseate tern	44	165

**Table 9:** The number of sea turtle records and animals published on SEAMAP that are protected under the US Endangered Species Act, based on the US Fish and Wildlife listings (E = endangered, T = threatened, E/T = populations are endangered and threatened; [10]).

Status	Species	Common Name	Records (n)	Animals (n)
T	*Caretta caretta*	Loggerhead	59,443	61,197
E/T	*Chelonia mydas*	Green	6,911	6,911
E	*Dermochelys coriacea*	Leatherback	4,127	4,173
E	*Eretmochelys imbricata*	Hawksbill	2,713	2,713
E	*Lepidochelys kempii*	Kemp's ridley	332	349
E/T	*Lepidochelys olivacea*	Olive ridley	2,344	2,425

For each of the eight key marine species chosen to compare among open-access databases, SEAMAP contributed varied amounts of data compared to what was available through GBIF and OBIS (Table 10). Although SEAMAP is a data node which contributes to OBIS, which in turn contributes to GBIF, this process is only periodically maintained and therefore not all SEAMAP data can currently be found on OBIS and GBIF. The most recent time when OBIS obtained data from SEAMAP was on November 16, 2009, one day prior to the data download for the gap analysis. As a result of the current data flow hierarchy, SEAMAP and OBIS were most similar in data holdings while GBIF and SEAMAP had a greater difference in numbers of records for each marine species examined. SEAMAP contributed the most data for species such as the bottlenose dolphin, great shearwater (*Puffinus gravis*), and loggerhead sea turtle, but was not a significant contributor for more rare species such as the sperm whale (*Physeter macrocephalus*/*P. catodon*).

**Table 10:** The number of records downloaded per database with latitude/longitude data and percent of the total comprised of SEAMAP records for eight key species; SEAMAP records on OBIS were identified by the information in the “Datapub” and “DataRight” columns, SEAMAP records on GBIF were identified by information in the “Res_name” and “Institutio” columns.

Species		Common Name	SEAMAP	OBIS	SEAMAP on OBIS	GBIF	SEAMAP on GBIF
Marine mammal							
	*Orcinus orca*	Killer whale	643	1,531	52%	1,297	33%
	*Physeter macrocephalus; P. catodon*	Sperm whale	2,460	20,097	12%	19,444	8%
	*Tursiops truncatus*	Bottlenose dolphin	17,903	18,737	98%	14,813	83%
Seabird							
	*Diomedea exulans*	Wandering albatross	2,360	18,102	12%	5,789	15%
	*Puffinus gravis*	Great shearwater	16,613	16,460	99%	24,557	65%
	*Sterna paradisaea*	Arctic tern	2,683	5,215	55%	68,434	1%
Sea turtle							
	*Caretta caretta*	Loggerhead	73,191	65,497	96%	20,541	91%
	*Dermochelys coriacea*	Leatherback	4,141	8,867	47%	1,385	34%

**Figure 8:**
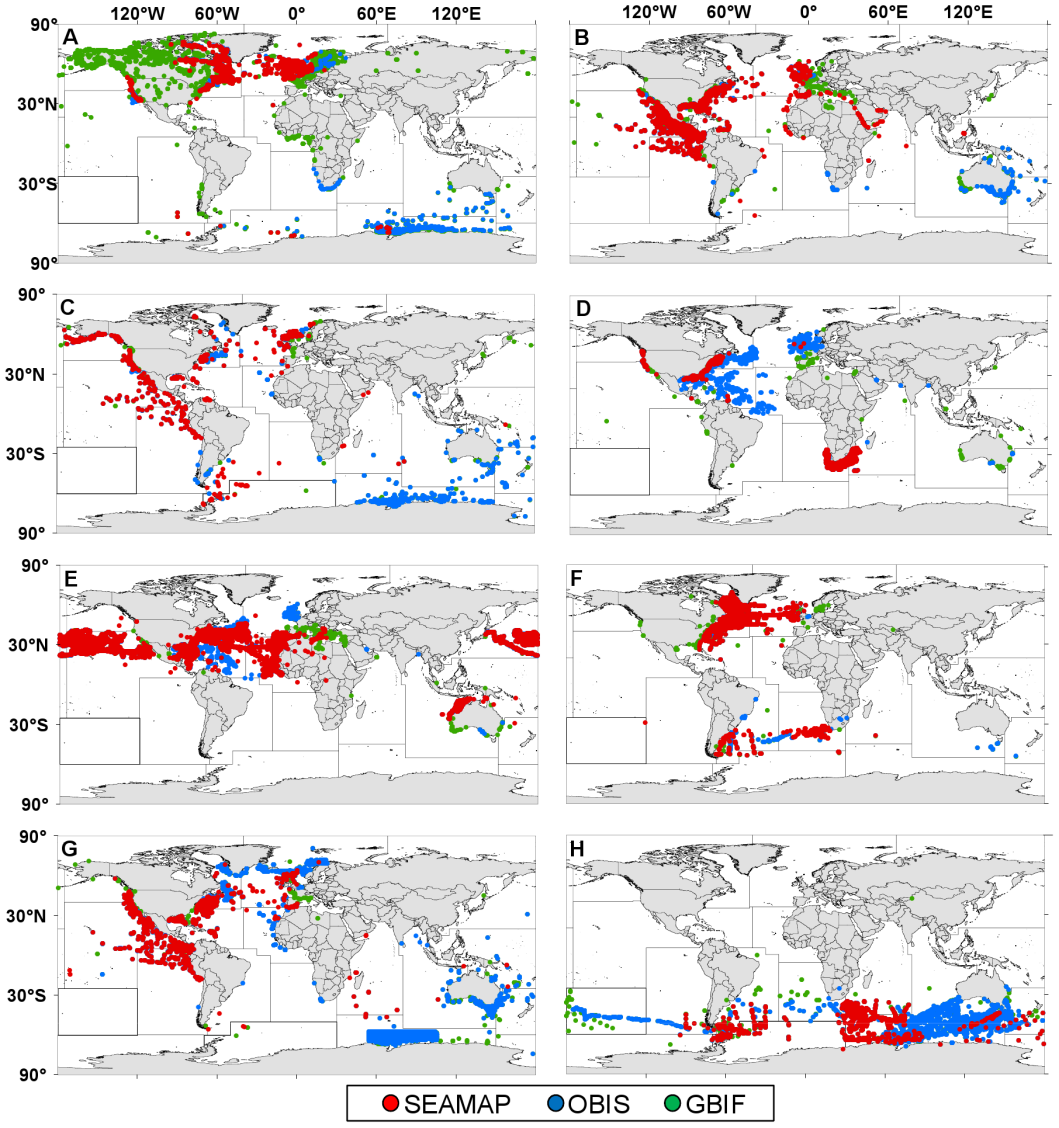
Geo-referenced records from SEAMAP, OBIS, and GBIF for eight species. SEAMAP (red points), OBIS (but not SEAMAP; blue points), and GBIF (but not OBIS or SEAMAP; green points) data for: (A) Arctic tern; (B) bottlenose dolphin; (C) killer whale; (D) leatherback sea turtle; (E) loggerhead sea turtle; (F) shearwater; (G) sperm whale; and (H) wandering albatross. Data were downloaded between November 17–18, 2009.

Most SEAMAP data for the eight key species were centralized in the mid-latitudes, having heavy representation around North America (Figures 8a–g), with the exception of the wandering albatross (*Diomedea exulans*) whose habitat range is in the southern oceans (Figure 8h). OBIS data not included on SEAMAP filled many geographic gaps for most species, especially contributing information in the Indian-Antarctic for Arctic tern (*Sterna paradisaea*), killer whale (*Orcinus orca*), sperm whale, and wandering albatross (Figures 8a, c, g, h, respectively). Around Australia, bottlenose dolphin, sperm whale, and wandering albatross data were lacking on SEAMAP, but were available through OBIS (Figures 8b, g, h, respectively). Data found exclusively on GBIF contributed some data in geographic gaps, most notably for Arctic tern records in the Pacific NE and SE, Atlantic NE and CE, and Arctic (Figure 8a). In addition, GBIF data filled data gaps in the Mediterranean for bottlenose dolphin, leatherback sea turtle *Dermochelys coriacea*, loggerhead sea turtle, and sperm whale (Figures 8b, d, e, g, respectively). However, SEAMAP generally had high data coverage for great shearwater, mostly lacking records found on GBIF near Europe and OBIS in the southwest Atlantic (Figure 8f).

## Discussion

When assessing current knowledge on species distributions with the use of multiple sources, it is important to take into account the effects of temporal and geographic sampling biases. Overall, SEAMAP dataset, record, and observation holdings per year have been increasing which likely is a result of increased effort for SEAMAP project data acquisition for more current data and not increased abundances of species in the field. Community awareness of the project and emphasis on data management for better archiving and/or dissemination has grown within recent years. On the other hand, geographic biases in research can be due to site accessibility, proximity to collecting agencies or highly human populated areas, or interest (either targeting areas with the high probability of species presence, or areas and species of high conservation concern) [13]. Although the SEAMAP project has not had a random approach to obtaining observations on marine mammals, seabirds, and sea turtles, this gap analysis is a first assessment to determine not only SEAMAP project gaps compared to the greater research community, but also to look into possible biases in research as a result of dataset holdings.

The majority of data contributed to the SEAMAP project has been seabird observations (with peak numbers of records in the 1980s) followed by high numbers of marine mammals (mainly occurring in the 1990s) and sea turtles (having increased observations after 2000). It is unknown as to why high numbers of records occurred when they did for each taxa or why seabird data made up the majority, especially since data acquisition for the SEAMAP project was sometimes on an ad hoc basis. A general increased amount of publications with time was also found for marine mammal diving and tracking studies, which may be attributed to increased computer access and improved technology for processing data [14]. One explanation for the differences in numbers of records among taxa may partly be influenced by inherent biases in detection based on taxa behavior and habitat (time spent in the air or on land versus under water). Seabirds usually occur in flocks and colonies, with higher numbers of individuals relative to pods or groups of marine mammals. Sea turtle observations may be least represented because they are generally solitary at sea and are also usually smaller with a lower profile than marine mammals, which makes detection more difficult. In addition, detectability for both marine mammals and sea turtles is low since they spend most of their time underwater. Therefore, other complementary methods may be more effective for informing habitat usage such as acoustic surveys (for marine mammals [15], [16]) and satellite telemetry [17]. Along with detectability differences, the number of observations collected over time may have been influenced by the number of active researchers within each taxa community, the amount of resources available for surveys (i.e., funding), and the general abundance of species within the areas surveyed.

Although data from shipboard and aerial surveys, satellite tags, and shore-based observations can be used to determine species ranges, abundances, and density estimates, there are distinct platform differences that need to be taken account. Shipboard surveys have the advantage of allowing for closer inspection of animals and additional biotic (i.e., prey abundance, acoustic monitoring, etc.) or abiotic (sea surface temperature, salinity, etc.) sampling. However, aerial surveys are less expensive and usually cover a larger extent in a shorter period of time compared to shipboard surveys. Avoidance or attraction to aerial or shipboard platforms may also influence density estimates, depending on certain species [18], [19]. Higher densities of common animals are generally found in aerial surveys due to greater visibility, but better detection of rare or small species occur with shipboard surveys [20], [21]. Greater detection may also be found with shore-based surveys for small, coastal marine mammals [22]. Furthermore, satellite telemetry tags are critical for capturing areas where animals traverse, filling in many of the spatial and temporal gaps left from surveys [23], [24]. For the data published on the SEAMAP website, most observations were from shipboard surveys and far fewer were from aerial surveys, tag, and shore. Shore-based surveys gave the most animals per record since locations of species were usually congregations (i.e., seal haul out sites and seabird colonies). Depending on the priorities for filling gaps within SEAMAP, the representation of certain species may be maximized by targeting aerial or shore surveys while more observations of less commonly sighted animals may be obtained through shipboard surveys.

While reviewing the full list of data already obtained for SEAMAP, it was recognized that there were significant temporal biases within a year, which may be a result of the combination of seasonal affects and regional source of the data. Datasets have primarily come from North America and northern Europe with large geographic gaps existing in the Southern Hemisphere and Indian Ocean. Since most data were located in the Northern Hemisphere, the summer (June–August) and fall (September–November) seasons had the highest number of observations and animals probably due to a combination of greater survey effort and animal activity. Summer weather conditions are usually more favorable for surveys. Summer is also the breeding season for most seabirds, where colonies flock onshore and are easily detected, with fall being a time for feeding and fledging [25]. Many marine mammals migrate towards the poles to feed during the summer [26] and therefore, an increased likelihood of detection occurs if surveys are planned in known migration or foraging areas. Sea turtle nesting season often occurs in the summer to fall when adult female sea turtles aggregate and come close to and onto the shore. The higher number of seabird observations, published on SEAMAP, found in winter (December–February) may be attributed to the greater number of observations in the Southern Hemisphere during breeding and birthing seasons (austral summer; n = 589,116) compared to fall (n = 188).

The high number of records and animals published on SEAMAP in the Northern Hemisphere, along with the relatively high density of effort in the eastern US coast, can be attributed to collaborators and cooperation from US government groups, which were the greatest contributor (i.e., National Oceanic and Atmospheric Administration). This bias was expected due to high US-based research efforts and because the SEAMAP project is led by Duke University, US, with more established partners in the US and Europe than other regions. In addition, the SEAMAP website is currently only available in English, which may serve as a barrier in some regions. Even if requests for data were written in the native language (non-English) of the recipient, some providers may not receive the full benefits of SEAMAP if the majority of targeted users cannot utilize an English-only website. A cost-benefit analysis of the website translation for SEAMAP is needed to determine if language is the greatest limitation for obtaining data in certain regions. Based on current SEAMAP data holdings, non-US groups are an important target community for filling gaps for specific taxa (i.e., seabirds) and regions.

The values and ranges of environmental variables associated with observations published on SEAMAP varied by taxa and can be influenced by platform. By combining available data across platforms, environmental ranges are more complete in relation to available habitat for species when compared to using one platform alone [27]. For example, satellite tag locations can cover many spatial/habitat gaps when combined with traditional survey data to acquire a better picture of the presence of animals in a region. Currently, habitat and density modeling techniques for mixing data from multiple platforms while incorporating abundance, residence time, and detectability have not yet been fully developed and present a worthwhile opportunity for future research.

The distribution of each environmental variable was only analyzed for large taxa groups (i.e., marine mammals, seabirds, and sea turtles), but the same analysis can be used for individual species within a taxa to determine if SEAMAP data holdings adequately cover the known range for that particular species. For example, ecological niche models developed for blue whales (*Balaenoptera musculus*) showed that occurrence from whaling data was highest around waters 1–4 km deep [28], but only 25% of marine mammal sightings on SEAMAP sightings were in waters >2 km deep. Other factors known to influence species occurrence that were not examined in this study (i.e., prey distributions, preferred benthic habitats) can also be used to predict areas of relatively high abundance. Although many records and sightings for blue whales are available on SEAMAP (Table 7), representation would be improved by focusing future dataset acquisition and/or research efforts towards filling the gap in knowledge within the species' ecological niche.

Tracklines representing survey effort from shipboard and aerial surveys, as well as satellite telemetry tag paths showed that SEAMAP coverage is specifically biased towards coastal areas, with particularly good coverage of the US Exclusive Economic Zone (EEZ). Coastal areas are more accessible for research and can be the primary habitat of many marine species. With the exception of shipboard surveys in the Caribbean, highest densities of tracklines published on SEAMAP were within US waters, probably as a result of more contributions from US partners and collaborators, many of which were gathering data under US government mandates to survey the US EEZ for marine animals. SEAMAP currently has few partners conducting research in regions with the largest gaps, such as the Arctic, southern Atlantic, Atlantic-Antarctic, Pacific CW and SW, and Pacific-Antarctic. Building relationships with organizations who gather data in these regions should be a priority, especially since data exist in these regions, based on the preliminary literature search. Additionally, obtaining data gathered further offshore (e.g., satellite tag data) should be a priority in filling spatial gaps.

The challenges for exhaustive literature searches and reviews typically include time constraints, language barriers, availability (access or subscription) to literature, and limitations of available electronic databases, catalogues, and search engines to detect all published and unpublished resources related to the study. The results of the preliminary literature search showed great potential for the research community to contribute data to fill many of the spatial and temporal gaps known within the SEAMAP data holdings, especially for the Atlantic SW and Pacific NW regions. Information gathered from direct contact with experts in the field definitely supplements literature searches [9], [29]. It was apparent that the dissemination of new information for marine mammal, seabird, and sea turtle distribution relied heavily on the taxa networks, underlining the importance of avenues outside of published literature (i.e., symposiums and conferences, e-mail listservs, project and database information posted on the web, etc.). Other sources that were extremely useful were published regional reviews of research on marine mammals, seabirds, or sea turtles (e.g., [30]–[32]). For most data requests, referencing published sources streamlined the process involved with identifying potential data for publication on the SEAMAP website. Alongside primary literature, internet searches for contributors (i.e., government agencies, non-governmental organizations, individuals or groups at universities) have been beneficial in identifying large datasets not yet included on SEAMAP. Since the rate of finding potential high quality datasets within the literature and online websites is still high at this point, continuing these data acquisition methods would be prudent in filling in the gaps.

When combining all available marine mammal, seabird, and sea turtle data from open-access online databases (SEAMAP, OBIS, and GBIF), it was revealed that SEAMAP ranged in its percent contribution and spatial coverage. OBIS is an aggregating portal and batch data flow from SEAMAP into OBIS, but currently there is no systematic method for integrating data from OBIS back into SEAMAP. This dichotomy seems to exist between OBIS and GBIF as well; although OBIS is a data contributor for GBIF, not all data on GBIF are available on OBIS. In addition, data published on SEAMAP are not simultaneously posted to OBIS, nor are OBIS data automatically synced with GBIF. While all three databases have the same objective for making data freely available, the SEAMAP project's unique features (e.g., visualization of survey effort, environmental data availability, and metadata details) make it particularly valuable for data providers to have their data published within SEAMAP and not only at OBIS or GBIF. However, consistent data accessibility through all three portals would be more beneficial for all users. In addition, a systematic mechanism using unique identifiers should be enforced at each node to ensure that data contributed to all three databases are not duplicated.

SEAMAP provides a convenient means for data dissemination and outreach to the public, researchers, and resource managers. Along with data users, data providers benefit from publishing datasets on SEAMAP because the results of their research, including published reports or articles, are highlighted to reach a broader audience and their data can be added to customized maps where it can be viewed in the context of oceanographic observations, other datasets, and in the context of global distribution maps. In addition, SEAMAP provides an organized, secure off-site archive, which can expedite future data requests and queries. Making these observations and analysis tools available to the global research community will facilitate improved ecological understanding of marine mammals, seabirds, and sea turtles as well as inform the management and conservation of these species. The digital archive increases interoperability while building a knowledge base that will enhance research possibilities and support evidence-based conservation [33], [34]. In the future, mechanisms for easy bidirectional data transport among SEAMAP, OBIS, and GBIF would improve data archiving and dissemination.

Baseline knowledge on marine species distributions will be fundamental in documenting any responses to consequences of environmental and human induced threats to marine mammal, seabird, and sea turtle populations. Many cases of marine species populations close to or already extinct go undocumented due to the lack of research and available information on the geographic ranges (breeding, adult, and vagrant), among other reasons [35]. Furthermore, data management becomes increasingly difficult as information accumulates and a cataloging system for the information needs to be established especially as scientific researchers and personnel turnover. Based on responses from major conservation agencies, the greatest limitation was lack of time and resources to collate data to publish on SEAMAP. Therefore, conservation projects should have objectives that include making data publicly available to reduce information loss. It is important to have the involvement of scientists in the process of publishing their data within a comprehensive and accessible inventory of marine species and their distributions in order to fully evaluate future threats to marine mammal, seabird, and sea turtle populations. This is particularly important for the 42 species already identified to be endangered or threatened [10]. The aggregation of effort from multiple research projects at a data center, such as SEAMAP, can broaden the data coverage in space and time for a more global perspective. Although thousands of records were available from SEAMAP for most of the endangered or threatened species identified, in addition to millions of records of more common species, there were many species for which SEAMAP lacks data. These and other rarely recorded species should be a priority for future data acquisition efforts.

The SEAMAP project continues to gain momentum, and this review revealed that temporal and spatial gaps were mostly a result of data acquisition effort (increase with time), development of regional partnerships and collaborations (Northern Hemisphere bias), ease of collecting more recent and available data (1990s to present), field data collection seasons (summer), and site accessibility (coastal habitats). Future directions should foster partnerships with researchers in the Southern Hemisphere that may involve data cataloging/formatting assistance, while targeting datasets containing species that are less abundant (endangered or threatened) and with limited representation within SEAMAP. These goals are feasible considering the literature search and feedback already received from taxa experts in the field. Results from the gap analysis can also provide direct feedback to the management agencies and marine mammal, seabird, and sea turtle communities on planning future research, should gaps in space and time remain after searching within available literature and databases.

## Materials and Methods

The gap analysis was conducted using all data contributed to and publicly available on SEAMAP from the project's inception until February 12, 2009. Although the SEAMAP project has not specifically restricted data acceptance to exact definitions of species within the broad categories of marine mammals, seabirds, and sea turtles, these taxa were more strictly defined for the purposes of the gap analysis. Marine mammals were defined as animals from the Class Mammalia and included all taxa under Orders Cetacea and Sirenia [26], [36]. Within Order Carnivora, sub-order Caniformia, all animals under Families Odobenidae, Otariidae and Phocidae were included along with sea otters (*Enhydra* spp., *Lutrograle* spp.) within the Family Mustelidae and the polar bear (Family Ursidae, *Ursus maritimus*). When sightings of Caniformia were recorded, it was assumed that these were sightings of an acceptable marine mammal species and it was included in the analysis. Seabirds were defined using the traditional classification, based on anatomy [25], [37], [38]. Generally, seabirds are from the Class Aves within the Order Ciconiiformes, including Families Alcidae, Diomedeidae, Fregatidae, Hydrobatidae, Laridae, Pelecanidae, Pelecanoididae, Phaethontidae, Phalacrocoracidae, Procellariidae, Spheniscidae, Stercorariidae, and Sulidae. When sightings of “Seabird” were recorded, it was not included since it was too general to assume that these were sightings using the traditional classification. Finally, sea turtles included all animals under Class Reptilia, Order Testudines, and under Families Cheloniidae and Dermochelyidae. When sightings of “Testudines” were recorded, it was assumed that these were sightings of an acceptable sea turtle species and it was included in the analysis. Observations that were publicly available but were not categorized as a marine mammal, seabird, or sea turtle (i.e., finfish, shorebirds, vessels, etc.) were excluded.

Datasets from all platforms (shipboard, aerial, shore, and tag) were included. Observations from aerial or shipboard surveys usually recorded the number of animals sighted. When this information was missing, it was assumed that the count was one individual. For satellite telemetry datasets, the number of animals per record was always one even though one individual tag may have multiple observations each with a different location, depending on how often data were recorded and published. Therefore, when calculating the abundance of animals sighted for one satellite tag, high numbers can result from multiple observations (total abundance = total number of observations at all locations * one individual), even though it essentially is a replicate of the same individual. Although this number is an inflation of actual animals sighted, this count method was used so that the spatial and temporal information associated with each transmitted data point would not be lost. In addition, observations from other dataset platforms (shipboard, aerial, and shore) were not guaranteed as unique individuals and replicate sightings are possible.

### Temporal distribution

The number of datasets containing data from any one year was summed to get an overall temporal distribution of data contributions. All observations, broken down by taxa, were categorized temporally into years, seasons, and months of observation to determine the year range of available data and to identify the time periods in which SEAMAP holds much or little data (number of records and number of individuals observed). In addition, tracklines published on SEAMAP were examined for seasonal coverage. Seasons were defined as: winter = December–February, spring = March–May, summer = June–August, and fall = September–November.

### Spatial distribution

All geo-referenced marine mammals, seabird, and sea turtle data were compiled and subsequently summarized by number of records and number of animals within the 19 major marine FAO global statistical fishing areas [7], [39] (Figure 4). Although these statistical areas are somewhat arbitrary, their boundaries were established by taking into account factors that are important for SEAMAP dataset holdings and acquisition, such as the natural oceanic regions and divisions, national boundaries, longitude and latitude grid system, and the distribution of aquatic fauna [39]. When features were summed per FAO region area, the known surface area was used [40]. Points were also summed within 1 degree cells to investigate biases within FAO regions (higher resolution). All spatial analyses were conducted using ArcGIS version 9.3 ArcInfo license [41].

Polyline tracklines were constructed with a series of unprojected straight lines connecting consecutive points along a survey track (when provided along with observation data) or satellite telemetry tag path and exported into ArcGIS [41]. If datasets delineated records as to when observers were “on-effort” or “off-effort” along a survey track, off-effort records/tracks were excluded. Recording intervals and survey methods (e.g., line-transect or opportunistic survey) varied among datasets. Final polyline shapefiles were published and available for download on the SEAMAP website.

The trackline density within each FAO region of all effort data published on SEAMAP was calculated after tracklines were projected using a customized Lambert Equal Area Azimuthal projection (mean center of all tracklines within the FAO region was used for the central meridian and latitude to minimize distortion). Although survey observation widths vary among platform types (i.e., aerial, shipboard, shore, and tag), the details of constructing accurate survey strip widths was not available, therefore a common method was applied to all datasets. The Line Density tool in ArcGIS Spatial Analyst [41] was used with a cell size of 12 km and 50 km search radius, resulting in a ∼100 km buffer around each trackline to help visualize general spatial patterns of trackline coverage. The summed length of tracklines per km^2^ was used to determine relative coverage within each FAO region, using areas calculated in ArcGIS. The percent area of trackline coverage within a region was calculated by dividing the number of cells with >0 km of trackline present by the total number of cells within a region.

### Oceanographic coverage

The date and location of each observation was used to sample environmental data from satellite imagery when available. Environmental data include bathymetry, sea surface temperature (SST), sea surface height (SSH), and chlorophyll *a* (Table 3). All environmental variables, except for bathymetry, were averaged over different time periods (i.e., yearly, monthly, weekly). The weekly SST average containing the observed date was applied to the day of each observation; the weekly average containing the first day of the month was used to determine monthly SSH; and the eight day average containing the observed date was applied to the day of each observation. The distribution of each environmental parameter was investigated by taxa and platform using the Kolmogorov-Smirnov-Lilliefors (KSL) test for normality [42]–[44] and Hartigan dip test for unimodality [45], [46]. Since sample sizes were large (n>5,000), the *p*-value was obtained using an interpolation of the dip statistic and sample size values obtained from tables by Hartigan and Hartigan (1985) and Martin Maechler's qDiptab function within the R statistical software package diptest version 0.25–2 [47]. Sample size and dip statistic was best fitted with the power function for the 0.999 tail (r^2^ = 0.9951, y = 0.4887x^−0.442^) and with the log function for the 0.01 tail (r^2^ = 0.9593, y = −0.043ln(x)+0.1327). Environmental variables were compared among taxa and platforms using the nonparametric Kruskal Wallis test [48]. Kernel density estimation plots were used to compare platforms for each taxa using the density.compare function within the R statistical software package sm version 2.2–3 [47], [49], [50].

### Identifying potential datasets

Potential high quality marine mammal, seabird, and sea turtle datasets, defined as dedicated surveys or satellite telemetry tag data with the minimum required variables, were identified by searching through literature (gray and peer-reviewed), websites, and after consultations with experts within the research network. The search targeted more timely datasets (data collected after 1983), with the priority towards regions that were known to have little to no data represented in SEAMAP (i.e., western Pacific, southern Atlantic, Indian Oceans). When data collected prior to 1983 and dedicated surveys for bycatch, strandings, and sites (i.e., colonies, caves) were found during the search, they were included in case these datasets were the only types available for the species/region/time period. Although review papers and references that presented summarized data or model outputs (derived products of raw individual observations, sightings, or satellite tag data) were included in the search to aid in further data acquisition efforts, these were weeded out of the gap analysis when identified to reduce duplication, with the exception of papers that included unpublished data observations. In other words, only the references for raw data were included in the quantified gap analysis.

All references found were categorized by taxa (i.e., marine mammal, seabird, or sea turtle) and by FAO region, based on the reported data published or presented. On rare occasions when it was difficult to obtain a copy of the reference, the title/abstract was used for noting taxa and FAO region of research. There may be cases where more data (i.e., other taxa/FAO regions observed) were collected during the study that was not presented and it is recognized that the summarized results can be an underestimate of the available data within these references. However, the small amount of data that were not published and not captured will not likely affect the overall results of the quantitative analysis.

This was a preliminary exercise to determine the presence or absence of datasets within regions that were currently represented in SEAMAP as data poor and to identify datasets, when present, that would be considered as high priority for acquisition. After all references were summarized by taxa for all regions, the sum of references for each taxa were divided into five equal quintiles. Regions were coded based on the quantity of references per square kilometer relative to the cumulative distribution (Low, 1: <20%; Medium-Low, 2: 20–40%, Medium, 3: 40–60%; Medium-High, 4: 60–80%, and High, 5: >80%). Regions were coded in the same manner based on the quantity of records relative to the cumulative distribution (Figure 4). The code for the quantity of records was subtracted from the code for the reference so that positive numbers represented a gap in SEAMAP holdings (relatively higher representation of references in the literature compared to relatively lower representation of records in SEAMAP). Likewise, negative numbers showed that SEAMAP holdings were relatively higher than references found during the preliminary literature search, suggesting that acquiring data in these references were of lower concern to fill gaps. This method was similar to one used previously to identify geographic gaps in sampling in relation to sample target distribution [51].

Data providers for datasets published on SEAMAP were also categorized as a government organization, non-government organization (i.e., non-profit research institutes, aquariums, etc.), or university. In addition, providers were categorized as either a US or non-US based organization. These categories were used to compare data contributions across different types of providers, by taxa and by platform, in order to help determine current biases and to direct future requests for datasets to fill known gaps.

### Comparing portal coverage with example species

In order to identify species gaps within SEAMAP, animals considered to be of high conservation priority were applied as examples for assessing marine mammal, seabird, and sea turtle species coverage. The number of records and animals observed and published on SEAMAP were summarized to determine the species with low or no representation. A search for any high quality datasets that included species with low SEAMAP data holdings was then conducted to see if the lack of data was due to a true information gap or a gap only in SEAMAP data holdings.

In addition, SEAMAP data holdings for key species from each taxa were used to compare data holdings among other open-access databases, such as the parent OBIS portal and the GBIF database that subsequently receives OBIS data. Key species were chosen within each taxa to cover a large global distribution when combined and included sperm whale, killer whale, bottlenose dolphin, great shearwater, Arctic tern, wandering albatross, leatherback sea turtle, and loggerhead sea turtle. Data were downloaded for each species from SEAMAP, GBIF, and OBIS websites between November 17–18, 2009. When possible, duplicate observations (based on the information provided within the record such as the data source and rights) were flagged and eliminated to determine the total available number of records and spatial coverage of species observations for each database. Since SEAMAP data can be part of OBIS and GBIF, SEAMAP data were flagged within OBIS and GBIF data to determine the percent contribution for each database. Although there may have been instances of overlap/duplication among data records that were not flagged (data points looked similar when mapped), insufficient information within the data records made it impossible to know for certain if it was a true duplicate and therefore records were assumed to be unique to be conservative.

## Supporting Information

Text S1 Bibliography from the preliminary literature search, with taxa and FAO region of data in bold. These references included data not published on OBIS-SEAMAP by February 2009.
(0.24 MB DOC)
