# Supplementary material for: Spatio-Temporal Gap Analysis of OBIS-SEAMAP Project Data: Assessment and Way Forward
Source: PLoS One. 2010 Sep 24;5(9):e12990. doi: 10.1371/journal.pone.0012990 (PMC2945317; doi:10.1371/journal.pone.0012990)
Supplement: Text S1 — Bibliography from the preliminary literature search, with taxa and FAO region of data in bold. These references included data not published on OBIS-SEAMAP by February 2009. (0.24 MB DOC) [file pone.0012990.s001.doc]

**Spatio-temporal gap analysis of OBIS-SEAMAP project data: Assessment and way forward**

Connie Y. Kot^1*^, Ei Fujioka^1^, Lucie J. Hazen^1^, Benjamin D. Best^1^, Andrew J. Read^2^, and Patrick N. Halpin^1^

^1^Marine Geospatial Ecology Lab, Nicholas School of the Environment, Duke University

^2^Nicholas School of the Environment, Duke University

*Corresponding author e-mail: connie.kot@duke.edu

**Text S1. Bibliography from the preliminary literature search, with taxa and FAO region of data in bold. These references included data not published on OBIS-SEAMAP by February 2009.**

1. Afsal VV, Yousuf KSSM, Anoop B, Anoop AK, Kannan P, et al. (2008) A note on cetacean distribution in the Indian EEZ and contiguous seas during 2003-07. J Cetacean Res Manag 10: 209-215. **marine mammal, Indian W, Indian E**

2. Aguayo A, Bernal R, Olavarría C, Vallejos V, Hucke R (1998) Observaciones de cetáceos realizadas entre Valparaíso e isla de Pascua, Chile, durante los inviernos de 1993, 1994 y 1995. Rev Biol Mar Oceanogr 33: 101–123. **marine mammal, Pacific SE**

3. Ainley DG, Dugger KM, Toniolo V, Gaffney I (2007) Cetacean occurrence patterns in the Amundsen and southern Bellingshausen Sea sector, Southern Ocean. Mar Mamm Sci 23: 287-305. **marine mammal, Pacific Antarctic**

4. Ainley DG, Spear LB, Tynan CT, Barth JA, Pierce SD, et al. (2005) Physical and biological variables affecting seabird distributions during the upwelling season of the northern California current. Deep Sea Res Part II 52: 123-143. **seabird, Pacific CE**

5. Airoldi S, Azzellino A, Nani B, Ballardini M, Bastoni C, et al. (1999) Whale watching in Italy: Results of the first three years of activity. Eur Res Cetacean 13: 153-156. **marine mammal, Mediterranean**

6. Aissi M, Celona A, Comparetto G, Mangano R, Wurtz M, et al. (2008) Large-scale seasonal distribution of fin whales (*Balaenoptera physalus*) in the central Mediterranean Sea. J Mar Biol Assoc U K 88: 1253-1261. **marine mammal, Mediterranean**

7. Alling A (1986) Records of odontocetes in the northern Indian Ocean (1981-1982) and off the coast of Sri Lanka (1982-1984). J Bombay Nat Hist 83: 376-394. **marine mammal, Indian W**

8. Alling A (1987) Records of odontocetes in the northern Indian Ocean and off Sri Lanka. J Bombay Nat Hist 83: 376-394. **marine mammal, Indian W**

9. Alling A (1988) A preliminary report of the incidental entrapment of odontocetes by Sri Lanka's coastal drift net fishery. J Bombay Nat Hist 85: 538-550. **marine mammal, Indian E**

10. Alling A, Dorsey EM, Gordon JCD (1991) Blue whales *Balaenoptera musculus* off the northeast coast of Sri Lanka: Distribution, feeding and individual identification. In: Leatherwood S, Donovan GP, editors. Cetaceans and cetacean research in the Indian Ocean Sanctuary, marine mammal technical report number 3. Nairobi, Kenya: United Nations Environment Program (UNEP). pp. 247-258. **marine mammal, Indian W**

11. Anderson RC (2005) Observations of cetaceans in the Maldives, 1990–2002. J Cetacean Res Manag 7: 119–135. **marine mammal, Indian W**

12. Andriolo A, Martins CCA, Engel MH, Pizzorno JL, Más-Rosa S, et al. (2006) The first aerial survey to estimate abundance of humpback whales (*Megaptera novaeangliae*) in the breeding ground off Brazil (breeding stock A). J Cetacean Res Manag 8: 307-311. **marine mammal, Atlantic SW**

13. Azzellino A, Gaspari S, Airoldi S, Nani B (2008) Habitat use and preferences of cetaceans along the continental slope and the adjacent pelagic waters in the western Ligurian Sea. Deep-Sea Res Part I: Oceanogr Res Pap 55: 296-323. **marine mammal, Mediterranean**

14. Baird IG, Mounsouphom B, Stacey PJ (1994) Preliminary surveys of Irrawaddy dolphins (*Orcaella brevirostris*) in Lao PDR and northeastern Cambodia. Rep Int Whaling Comm 44. **marine mammal, Pacific CW**

15. Baird RW, Langelier KM, Stacey PJ (1989) First records of false killer whales, *Pseudorca crassidens*, in Canada. Can Field-Nat 103: 368-371. **marine mammal, Pacific NE**

16. Balazs GH (1993) Homeward bound: Satellite tracking of Hawaiian green turtles from nesting beaches to foraging pastures. In: Schroeder BA, Witherington BE, editors. 13th annual symposium on sea turtle biology and conservation. Miami, FL: NOAA Technical Memorandum NMFS-SEFSC-341. pp. 205-208. **sea turtle, Pacific CE**

17. Balazs GH, Craig P, Winton BR, Miya RK (1994) Satellite telemetry of green turtles nesting at French Frigate Shoals, Hawaii, and Rose Atoll, American Samoa. In: Bjorndal K, Bolten A, Johnson D, editors. 14th annual symposium on sea turtle biology and conservation. Miami, FL: NOAA Technical Memorandum NMFS-SEFSC-351. pp. 184-187. **sea turtle, Pacific NE, Pacific SW**

18. Baldwin R (1996) Marine mammals of the United Arab Emirates. In: Vine PJ, editor. Natural Emirates: Wildlife and environment of the United Arab Emirates. London, England: Trident Press. pp. 199-212. **marine mammal, Indian W**

19. Baldwin R, Gallagher M, Van Waerebeek K (1998) A review of cetaceans from waters off the Arabian Peninsula. In: Fisher M, Ghazanfar SA, Spalton A, editors. The natural history of Oman: A festschrift for Michael Gallagher. Leiden, Netherlands: Backhuys Publishers. pp. 161–189. **marine mammal, Indian W**

20. Baldwin R, Salm R (1994) Whales and dolphins along the coast of Oman. Seeb, Oman: Robert Baldwin. **marine mammal, Indian W**

21. Ballance LT (2008) Understanding seabirds at sea: Why and how? Mar Ornithol 35: 127-135. **seabird, Pacific CE, Pacific SE**

22. Ballance LT, Anderson RC, Pitman RL, Stafford K, Shaan A, et al. (2001) Cetacean sightings around the Republic of the Maldives, April 1998. J Cetacean Res Manag 3: 213–218. **marine mammal, Indian W**

23. Ballance LT, Pitman RL (1998) Cetaceans of the western tropical Indian Ocean: Distribution, relative abundance, and comparisons with cetacean communities of two other tropical ecosystems. Mar Mamm Sci 14: 429-459. **marine mammal, Indian W**

24. Ballance LT, Pitman RL, Reilly SB, Force MP (1996) Report of a cetacean, seabird, marine turtle and flying fish survey of the western tropical Indian Ocean aboard the research vessel Malcolm Baldrige, March 21–July 26, 1995. Seattle, WA: NOAA Technical Memorandum NMFS-NWFSC 224. **marine mammal, seabird, sea turtle, Indian W, Indian E**

25. Balmer BC, Wells RS, Nowacek SM, Nowacek DP, Schwacke LH, et al. (2008) Seasonal abundance and distribution patterns of common bottlenose dolphins (*Tursiops truncatus*) near St. Joseph Bay, Florida, USA. J Cetacean Res Manag 10: 157-167. **marine mammal, Atlantic CW**

26. Bannister JL (1968) An aerial survey for sperm whales off the coast of Western Australia 1963-1965. Aust J Mar Freshw Res 19: 31-52. **marine mammal, Indian E**

27. Bannister JL (1979-2005) Annual reports on aerial surveys of southern right whales on the southern Australian coastline. Perth, Australia: Western Australian Museum, Perth. **marine mammal, Indian E, Pacific SW**

28. Bannister JL (1986) Notes on nineteenth century catches of southern right whales (*Eubalaena australis*) off the southern coasts of Western Australia. Rep Int Whaling Comm Special Issue 10: 255-259. **marine mammal, Indian E**

29. Bannister JL (1990) Southern right whales off Western Australia. Rep Int Whaling Comm Special Issue 12: 279-288. **marine mammal, Indian E**

30. Bannister JL (2001) Status of southern right whales (*Eubalaena australis*) off southern Australia. J Cetacean Res Manag Special Issue 2: 103-110. **marine mammal, Indian E, Pacific SW**

31. Bannister JL (2003) Southern right whale aerial survey and photo-identification, southern Australia 2002. Final report to Environment Australia. Perth, Australia: Western Australian Museum, Perth. **marine mammal, Indian E**

32. Bannister JL, Pastene LA, Burnell SR (1999) First records of movement of a southern right whale (*Eubalaena australis*) between warm water breeding grounds and the Antarctic Ocean, south of 60 degrees S. Mar Mamm Sci 15: 1337-1342. **marine mammal, Indian Antarctic, Pacific Antarctic**

33. Barber DG, Saczuk E, Richard PR (2001) Examination of beluga-habitat relationships through the use of telemetry and a geographic information system. Arctic 54: 305-316. **marine mammal, Arctic**

34. Barber-Meyer SM, Kooyman GL, Ponganis PJ (2007) Estimating the relative abundance of emperor penguins at inaccessible colonies using satellite imagery. Polar Biol 30: 1565-1570. **seabird, Atlantic Antarctic**

35. Barham EG (1982) Marine mammals in Monterey Bay, California, during the years 1950-1955. Calif Fish Game 68: 213-223. **marine mammal, Pacific CE**

36. Barlas ME (1999) The distribution and abundance of harbor seals (*Phoca vitulina concolor*) and gray seals (*Halichoerus grypus*) in southern New England, winter 1998–summer 1999. Boston, MA: Boston University. **marine mammal, Atlantic NW**

37. Barlow J (1988) Harbor porpoise, *Phocoena phocoena*, abundance estimation for California, Oregon, and Washington: I. Ship surveys. Fish Bull 86: 417-432. **marine mammal, Pacific CE, Pacific NE**

38. Barlow J (2006) Cetacean abundance in Hawaiian waters estimated from a summer/fall survey in 2002. Mar Mamm Sci 22: 446-464. **marine mammal, Pacific CE**

39. Barlow J, Fleischer L, Forney KA, Maravillachavez O (1993) An experimental aerial survey for vaquita (*Phocoena sinus*) in the northern Gulf of California, Mexico. Mar Mamm Sci 9: 89-94. **marine mammal, Pacific NE**

40. Barlow J, Forney KA (2007) Abundance and population density of cetaceans in the California current ecosystem. Fish Bull 105: 509-526. **marine mammal, Pacific NE**

41. Barlow J, Oliver CW, Jackson TD, Taylor BL (1988) Harbor porpoise, *Phocoena phocoena*, abundance estimation for California, Oregon, and Washington: II. Aerial surveys. Fish Bull 86: 433-444. **marine mammal, Pacific CE, Pacific NE**

42. Baumgartner MF (1997) The distribution of Risso's dolphin (*Grampus griseus*) with respect to the physiography of the northern Gulf of Mexico. Mar Mamm Sci 13: 614-638. **marine mammal, Atlantic CW**

43. Baumgartner MF, Cole TVN, Clapham PJ, Mate BR (2003) North Atlantic right whale habitat in the lower Bay of Fundy and on the SW Scotian shelf during 1999-2001. Mar Ecol Prog Ser 264: 137-154. **marine mammal, Atlantic NW**

44. Baumgartner MF, Mate BR (2003) Summertime foraging ecology of North Atlantic right whales. Mar Ecol Prog Ser 264: 123-135. **marine mammal, Atlantic NW**

45. Baumgartner MF, Mate BR (2005) Summer and fall habitat of North Atlantic right whales (*Eubalaena glacialis*) inferred from satellite telemetry. Can J Fish Aquat Sci 62: 527-543. **marine mammal, Atlantic NW**

46. Bearzi G, Agazzi S, Bonizzoni S, Costa M, Azzellino A (2008) Dolphins in a bottle: Abundance, residency patterns and conservation of bottlenose dolphins *Tursiops truncatus* in the semi-closed eutrophic Amvrakikos Gulf, Greece. Aquat Conserv: Mar Freshwat Ecosyst 18: 130-146. **marine mammal, Mediterranean**

47. Bearzi M, Saylan CA, Feenstra J (2009) Seabird observations during cetacean surveys in Santa Monica Bay, California. Bull South Calif Acad Sci 108: 63-69. **marine mammal, seabird, Pacific CE**

48. Beasley IL, Davidson PJA (2007) Conservation status of marine mammals in Cambodian waters, including seven new cetacean records of occurrence. Aquat Mamm 33: 368-379. **marine mammal, Pacific CW**

49. Belgrano J, Cesar G, Arcucci D, Krohling F, Miguel I (2007) Recently increasing in the number of southern right whales in Golfo San Jorge, Santa Cruz, Patagonia Argentina. 17th biennial conference on the Biology of Marine Mammals (SMM), 29 November - 3 December 2007. Capetown, South Africa. **marine mammal, Atlantic SW**

50. Belgrano J, Iñíguez M, Gibbons J, García C, Olavarría C (2008) South-west Atlantic right whales *Eubalaena australis* (Desmoulins, 1822) distribution nearby the Magellan Strait. Anales Instituto Patagonia (Chile) 36: 69-74. **marine mammal, Atlantic SW**

51. Bengtson JL, Ferm LM, Härkönen TJ, Stewart BS (1990) Abundance of Antarctic fur seals in the South Shetland Islands, Antarctica, during the 1986/87 austral summer. In: Kerry KR, Hempel G, editors. Antarctic ecosystems, ecological change and conservation. Berlin, Germany: Springer-Verlag. pp. 265-270. **marine mammal, Atlantic Antarctic**

52. Benson SR (2005) Leatherback turtle nesting demographics: Identified through migratory movements and aerial census in Papua New Guinea. In: Kinan I, editor. Western Pacific Sea Turtle Cooperative Research and Management Workshop. Honolulu, HI: Western Pacific Regional Fishery Management Council. pp. 43-46. **sea turtle, Pacific CW**

53. Benson SR, Forney KA, Harvey JT, Carretta JV, Dutton PH (2007) Abundance, distribution, and habitat of leatherback turtles (*Dermochelys coriacea*) off California, 1990-2003. Fish Bull 105: 337-347. **marine mammal, Pacific NE**

54. Benson SR, Kisokau KM, Ambio L, Rei V, Dutton PH, et al. (2007) Beach use, internesting movement, and migration of leatherback turtles, *Dermochelys coriacea*, nesting on the north coast of Papua New Guinea. Chelonian Conserv Biol 6. **sea turtle, Pacific CW**

55. Berrow S, Whooley P, Wall D (2006) ISCOPE – Irish scheme for cetacean observation and public education (Final Report 2003-2005). Kilrush, Ireland: Irish Whale and Dolphin Group. **marine mammal, Atlantic NE**

56. Best PB, Glass JP, Ryan PG, Dalebout ML (2009) Cetacean records from Tristan da Cunha, South Atlantic. J Mar Biol Assoc U K 89: 1023-1032. **marine mammal, Atlantic SE**

57. Best PB, Mate BR (2007) Sighting history and observations of southern right whales following satellite tagging off South Africa. J Cetacean Res Manag 9: 111-114. **marine mammal, Atlantic SE**

58. Best PB, Rademeyer RA, Burton C, Ljungblad D, Sekiguchi K, et al. (2003) The abundance of blue whales on the Madagascar Plateau, December 1996. J Cetacean Res Manag 5: 253-260. **marine mammal, Indian W**

59. BirdLife International (2004) Tracking ocean wanderers: The global distribution of albatrosses and petrels. Results from the Global Procellariiform Tracking Workshop, 1-5 September, 2003, Gordon's Bay, South Africa. Cambridge, UK: Birdlife International. **seabird, Pacific NE, Pacific CE, Pacific SW, Pacific SE, Pacific Antarctic, Atlantic SW, Atlantic SE, Atlantic Antarctic, Indian W, Indian Antarctic, Pacific NW**

60. Black N, Ternullo R (2007) Ecology of mammal-hunting ("transient") killer whales in Monterey Bay, CA: A 20-year study. 17th biennial conference on the Biology of Marine Mammals (SMM), 29 November - 3 December 2007. Capetown, South Africa. **marine mammal, Pacific CE, Pacific NE**

61. Bleakney JS (1965) Reports of marine turtles from New England and eastern Canada. Can Field-Nat 79: 120-128. **sea turtle, Atlantic NW**

62. Blokhin S (1996) Distribution, abundance and behavior of gray whales (*Eschrichtius robustus*) of American and Asian populations in regions of their summer location nearshore of the far east. Izv TINRO Tsentra 121: 36-53. **marine mammal, Pacific NW**

63. Blokhin SA (1981) The results of the cruise of the whaler Recordniy in the coastal waters of Australia in March 1979. Rep Int Whaling Comm 31: 723–727. **marine mammal, Indian E**

64. Blokhin SA, Burdin AM (2001) Distribution, abundance and some traits of behavior of the gray whale *Eschrichtius robustus* of the Korean stock at the northeastern coast of Sakhalin. Biologiya morya 27: 15-20. **marine mammal, Pacific NW**

65. Blokhin SA, Doroshenko NV, Yazvenko SB (2004) Distribution, abundance, and movement patterns of western gray whales (*Eschrichtius robustus*) off the coast of northeastern Sakhalin Island in June-December 2003 based on aerial survey data. Research program annual report. Vladivostok, Russia: All-Russian Research Institute of Fisheries and Oceanography (VNIRO), Exxon Neftegaz Limited (ENL) and Sakhalin Energy Investment Company, Ltd. **marine mammal, Pacific NW**

66. Bluhm BA, Coyle KO, Konar B, Highsmith R (2007) High gray whale relative abundances associated with an oceanographic front in the south-central Chukchi Sea. Deep Sea Res Part II 54: 14. **marine mammal, Arctic, Pacific CE**

67. Bodkin JL, Udevitz MS (1999) An aerial survey method to estimate sea otter abundance. In: Garner GW, Armstrup SC, Laake JL, Manly BFJ, McDonald LL et al., editors. Marine mammal survey and assessment methods. Rotterdam, Netherland: A. A. Balkema. pp. 13-26. **marine mammal, Pacific NE**

68. Boisseau O, Lacey C, Lewis T, Matthews JN, Moscrup A, et al. (2008) Sighting rates from cetacean surveys in the Mediterranean between 2003 and 2007. Eur Res Cetacean 21: 1. **marine mammal, Mediterranean**

69. Boisseau O, Matthews J, Gillespie D, Lacey C, Moscrop A, et al. (2007) A visual and acoustic survey for harbour porpoises off north-west Africa: Further evidence of a discrete population. Afr J Mar Sci 29: 403-410. **marine mammal, Atlantic CE**

70. Bolaños J, Villarroel-Marin A (2003) Three new records of cetacean species for Venezuelan waters. Caribb J Sci 39: 230-232. **marine mammal, Atlantic CW**

71. Bolaños-Jiménez J, Velasquez F (2007) Aquatic mammals of the Venezuelan Atlantic in the context of oil and gas exploration and development activities. 17th biennial conference on the Biology of Marine Mammals (SMM), 29 November - 3 December 2007. Capetown, South Africa. **marine mammal, Atlantic CW**

72. Bolt HE, Harvey PV, Mandleberg L, Foote AD (2009) Occurrence of killer whales in Scottish inshore waters: Temporal and spatial patterns relative to the distribution of declining harbour seal populations. Aquat Conserv: Mar Freshwat Ecosyst 19: 671-675. **marine mammal, Atlantic NE**

73. Born EW, Knutsen LO (1992) Satellite-linked radio tracking of Atlantic walruses (*Odobenus rosmarus rosmarus*) in northeastern Greenland, 1989-1991. Z Saugetierkd 57: 275-287. **marine mammal, Atlantic NE**

74. Born EW, Knutsen LO (1997) Haul-out and diving activity of male Atlantic walruses (*Odobenus rosmarus rosmarus*) in NE Greenland. J Zool 243: 381-396. **marine mammal, Atlantic NE**

75. Born EW, Rysgaard S, Ehlmé G, Sejr M, Acquarone M, et al. (2003) Underwater observations of foraging free-living Atlantic walruses (*Odobenus rosmarus rosmarus*) and estimates of their food consumption. Polar Biol 26: 348-357. **marine mammal, Atlantic NE**

76. Born EW, Teilmann J, Acquarone M, Riget FF (2004) Habitat use of ringed seals (*Phoca hispida*) in the North Water area (North Baffin Bay). Arctic 57: 129(114). **marine mammal, Atlantic NW**

77. Born EW, Teilmann J, Riget F (2002) Haul-out activity of ringed seals (*Phoca hispida*) determined from satellite telemetry. Mar Mamm Sci 18: 167-181. **marine mammal, Atlantic NW**

78. Borsa P, Hoarau G (2004) A pygmy blue whale (Cetacea: Balaenopteridae) in the inshore waters of New Caledonia. Pac Sci 58: 579-584. **marine mammal, Pacific CW**

79. Boveng P, London J, Montgomery R (2007) Strong seasonal dynamics of harbor seals in Cook Inlet, Alaska. 17th biennial conference on the Biology of Marine Mammals (SMM), 29 November - 3 December 2007. Capetown, South Africa. **marine mammal, Pacific NE**

80. Bowles AE, Smultea M, Wursig B, DeMaster DP, Palka D (1994) Relative abundance and behavior of marine mammals exposed to transmissions from the Heard Island feasibility test. J Acoust Soc Am 96: 2469–2484. **marine mammal, Indian Antarctic**

81. Branch TA, Butterworth DS (2001) Estimates of abundance south of 60degreeS for cetacean species sighted frequently on the 1978/79 to 1997/98 IWC/IDCR-SOWER sighting surveys. J Cetacean Res Manag 3: 251-270. **marine mammal, Atlantic Arctic, Pacific Antarctic, Indian Antarctic**

82. Branch TA, Stafford KM, Palacios DM, Allison C, Bannister JL, et al. (2007) Past and present distribution, densities and movements of blue whales *Balaenoptera musculus* in the southern hemisphere and northern Indian Ocean. Mammal Rev 37: 116-175. **marine mammal, Indian W, Indian E, Pacific CE, Atlantic CW, Pacific SW, Pacific Antarctic, Pacific SE, Atlantic CE, Atlantic SW, Atlantic SE, Atlantic Antarctic, Indian Antarctic, Pacific CW**

83. Braulik GT (2006) Status assessment of the Indus River dolphin, *Platanista gangetica minor*, March-April 2001. Biol Conserv 129: 579-590. **marine mammal, Indian W**

84. Brereton T, Wall D, Cermeno P, Vasquez A, Curtis D, et al. (2001) Cetacean monitoring in north-west European waters. Atlantic Research Coalition (ARC) report number 1 - 2001. Dorset, UK: Atlantic Research Coalition (ARC). **marine mammal, Atlantic NE**

85. Brereton TM, Williams AD, Williams R (1999) Distribution and relative abundance of the common dolphin (*Delphinus delphis*) in the Bay of Biscay. Eur Res Cetacean 13: 295–299. **marine mammal, Atlantic NE**

86. Brito C, Vleira N, Sa E, Carvalho I (2009) Cetaceans' occurrence off the west central Portugal coast: A compilation of data from whaling, observations of opportunity and boat-based surveys. J Mar Anim Ecol 2: 10-13. **marine mammal, Atlantic NE**

87. Brueggeman JJ, Green GA, Grotefendt RA, Tressler RW, Chapman DG. Marine mammal habitat use in the north Aleutian Basin, St. George Basin, and Gulf of Alaska. In: Jarvela LE, Thorsteinson LK, editors; 1989; Anchorage, Alaska. U.S. Department of the Interior, Mineral Management Services. pp. 97-108. **marine mammal, Pacific NE**

88. Buckland ST, Cattanach KL, Miyashita T (1992) Minke whale abundance in the northwest Pacific and the Okhotsk Sea, estimated from 1989 and 1990 sightings surveys. Rep Int Whaling Comm 42: 387-391. **marine mammal, Pacific NW**

89. Burton CLK, Salgado-Kent CP, Glencross RG, Sturrock VJ, Jenner CP, et al. (2007) Distribution and relative abundance of blue whales in Geographe Bay, Western Australia. 17th biennial conference on the Biology of Marine Mammals (SMM), 29 November - 3 December 2007. Capetown, South Africa. **marine mammal, Indian E**

90. Cabrera E, Carlson C, Galletti Vernazzani B (2005) Presence of blue whale (*Balaenoptera musculus*) in the northwestern coast of Chiloe Island, southern Chile. Latin Amer J Aquat Mamm 4: 73–74. **marine mammal, Pacific SE**

91. Caldas M, Cedeira J, Pierce G, Santos M, Lopez A, et al. (2007) Trends in cetacean sightings along the Galician coast, NW Spain, 2003-2007. 17th biennial conference on the Biology of Marine Mammals (SMM), 29 November - 3 December 2007. Capetown, South Africa. **marine mammal, Atlantic NE**

92. Cañadas A, Lopez A, De Stephanis R, Martin V, Villalba N, et al. (2004) The first cetacean sighting network in Spain: Results of a co-operation within the Spanish Cetacean Society. Eur Res Cetacean 15: 378-380. **marine mammal, Atlantic NE, Atlantic CE**

93. Cañadas A, Sagarminaga R, Garcia-Tiscar S (2002) Cetacean distribution related with depth and slope in the Mediterranean waters off southern Spain. Deep-Sea Res Part I: Oceanogr Res Pap 49: 2053-2073. **marine mammal, Mediterranean**

94. Canado M, Servidio A, Lorenzo C, Vidal M (2007) Distribution, frequency and group composition of the oceanic form of the Atlantic spotted dolphin (*Stenella frontalis*) in the Canary Islands. 17th biennial conference on the Biology of Marine Mammals (SMM), 29 November - 3 December 2007. Capetown, South Africa. **marine mammal, Atlantic CE**

95. Carlstrom J, Denkinger J, Feddersen P, Øien N (1997) Record of a new northern range of Sowerby’s beaked whale (*Mesoplodon bidens*). Polar Biol 17: 459-461. **marine mammal, Atlantic NE**

96. Carretta JV, Barlow J, Forney KA, Muto MM, Baker J (2001) U.S. Pacific marine mammal stock assessments: 2001. La Jolla, CA: NOAA Technical Memorandum NOAA-TM-NMFS-SWFSC-317. **marine mammal, Pacific CE, Pacific NE**

97. Carretta JV, Forney KA (2004) Preliminary estimates of harbor porpoise abundance in California from 1999 and 2002 aerial surveys. La Jolla, CA: NOAA-NMFS-SWFSC Administrative report LJ-04-01. **marine mammal, Pacific NE**

98. Carretta JV, Forney KA, Laake JL (1998) Abundance of southern California coastal bottlenose dolphins estimated from tandem aerial surveys. Mar Mamm Sci 14: 655-675. **marine mammal, Pacific NE**

99. Carvalho M, Rossi-Santos M, Baracho C, Cipolotti S, Lima F, et al. (2007) Opportunistic sightings of dolphins in the north coast of Bahia State, northeastern Brazil, between 2001 and 2006. 17th biennial conference on the Biology of Marine Mammals (SMM), 29 November - 3 December 2007. Capetown, South Africa. **marine mammal, Atlantic SW**

100. Certain G, Bretagnolle V (2008) Monitoring seabirds population in marine ecosystem: The use of strip-transect aerial surveys. Remote Sens Environ 112: 3314-3322. **seabird, Atlantic NE**

101. Certain G, Ridoux V, Van Canneyt O, Bretagnolle V (2008) Delphinid spatial distribution and abundance estimates over the shelf of the Bay of Biscay. ICES J Mar Sci 65: 11. **marine mammal, Atlantic NE**

102. Chaloupka MY, Osmond MG (1999) Spatial and seasonal distribution of humpback whales in the Great Barrier Reef region. In: Musick JA, editor. Life in the slow lane: Ecology and conservation of long-lived marine animals. Washington, DC: American Fisheries Society. **marine mammal, Pacific CW**

103. Charuchinda M, Monanunsap S (1998) Monitoring survey on sea turtle nesting in the inner Gulf of Thailand, 1994-1994. Thai Mar Fish Res Bull 6: 17-25. **sea turtle, Pacific CW**

104. Chen C-T, Liu K-M, Huang E (1998) Abundance estimate of small cetacean in southwestern Taiwan waters. Paper SC/49/SM47 presented to the International Whaling Commission Scientific Committee. **marine mammal, Pacific NW**

105. Clapham P, Barlow J, Bessinger M, Cole T, Mattila D, et al. (2003) Abundance and demographic parameters of humpback whales from the Gulf of Maine, and stock definition relative to the Scotian shelf. J Cetacean Res Manag 5: 13-22. **marine mammal, Atlantic NW**

106. Clapham P, Zerbini A (2007) Satellite tagging humpbacks in the South Pacific: Windows on the whales’ world. Seattle, WA: NOAA-NMFS-AFSC Quarterly Report October-November-December 2007. **marine mammal, Pacific SW**

107. Clapham PJ, Young SB, Brownell Jr RL (1999) Baleen whales: Conservation issues and the status of the most endangered populations. Mammal Rev 29: 37–62. **marine mammal, Arctic**

108. Clarke ED, Spear LB, McCracken ML, Marques FFC, Borchers DL, et al. (2003) Validating the use of generalized additive models and at-sea surveys to estimate size and temporal trends of seabird populations. J Appl Ecol 40: 278-292. **seabird, Pacific CE**

109. Clarke JT, Moore SE, Ljungblad DK (1987) Observations of bowhead whale (*Balaena mysticetus*) calves in the Alaskan Beaufort Sea during the autumn migration, 1982-85. Rep Int Whaling Comm: 287-293. **marine mammal, Arctic**

110. Clarke JT, Norman SA (2005) Results and evaluation of US Navy shock trial environmental mitigation of marine mammals and sea turtles. J Cetacean Res Manag 7: 43-50. **marine mammal, sea turtle, Atlantic CW**

111. Collins T, Pomilla C, Ngouessono S, Cerchio S, Findlay K, et al. (2007) Population characteristics of humpback whales (*Megaptera novaeangliae*) wintering along the coast of Gabon. 17th biennial conference on the Biology of Marine Mammals (SMM), 29 November - 3 December 2007. Capetown, South Africa. **marine mammal, Atlantic SE**

112. Collum LA, Fritts TH (1985) Sperm whales (*Physeter catodon*) in the Gulf of Mexico. Southwest Nat 30: 101-104. **marine mammal, Atlantic CW**

113. Consiglieri LD, Braham HW, Dahlheim ME, Fiscus C, McGuire PD, et al. (1982) Seasonal distribution and relative abundance of marine mammals in the Gulf of Alaska. Final report. Juneau, AK: Outer Continental Shelf Environmental Assessment Program 61. **marine mammal, Pacific NE**

114. Constantine R, Visser I, Buurman D, Buurman R, McFadden B (1998) Killer whale (*Orcinus orca*) predation on dusky dolphins (*Lagenorhynchus obscurus*) in Kaikoura, New Zealand. Mar Mamm Sci 14: 324-330. **marine mammal, Atlantic SW**

115. Corbett HD (1994) The occurrence of cetaceans off Mauritius and in adjacent waters. Rep Int Whaling Comm 44: 393-397. **marine mammal, Indian W**

116. Craig P, Parker D, Brainard R, Rice M, Balazs G (2004) Migrations of green turtles in the central South Pacific. Biol Conserv 116: 433-438. **sea turtle, Pacific SW**

117. Cremer MJ, Simoes-Lopes PC, Pires JSR (2009) Occupation pattern of a harbor inlet by the estuarine dolphin, *Sotalia guianensis* (P. J. van Beneden, 1864) (Cetacea, Delphinidae). Braz Arch Biol Technol 52: 765-774. **marine mammal, Atlantic SW**

118. Cunningham L, Baxter JM, Boyd IL, Duck CD, Lonergan M, et al. (2009) Harbour seal movements and haul-out patterns: Implications for monitoring and management. Aquat Conserv: Mar Freshwat Ecosyst 19: 398-407. **marine mammal, Atlantic NE**

119. Danilewicz D, Secchi ER, Ott PH, Moreno IB, Bassoi M, et al. (2009) Habitat use patterns of franciscana dolphins (*Pontoporia blainvillei*) off southern Brazil in relation to water depth. J Mar Biol Assoc U K 89: 943-949. **marine mammal, Atlantic SW**

120. Davis RW, Evans WE, B. W (2000) Cetaceans, sea turtles and seabirds in the northern Gulf of Mexico: Distribution, abundance and habitat associations, Vol. II, Technical report. OCS Study MMS 2000-003. New Orleans, LA: US Department of the Interior, US Geological Survey, Biological Resources Division, and Minerals Management Service, Gulf of Mexico OCS Region USGS/BRD/CR-1999-0006. **marine mammal, seabird, sea turtle, Atlantic CW**

121. Davis RW, Fargion GS, editors (1996) Distribution and abundance of cetaceans in the north-central and western Gulf of Mexico. Final report, Volume II: Technical report. Prepared by the Texas Institute of Oceanography and the National Marine Fisheries Service. New Orleans, LA: Minerals Management Service, Gulf of Mexico, OCS Region OCS Study MMS 96-0027. **marine mammal, Atlantic CW**

122. Davis RW, Fargion GS, editors (1996) Distribution and abundance of cetaceans in the north-central and western Gulf of Mexico, Final report. New Orleans, LA: Minerals Management Service, Gulf of Mexico OCS Region OCS Study MMS 96-0028. **marine mammal, Atlantic CE**

123. de Silva PHDH (1987) Cetaceans (whales, dolphins and porpoises) recorded off Sri Lanka, India, from the Arabian Sea and Gulf, Gulf of Aden and from the Red Sea. J Bombay Nat Hist 84: 504-525. **marine mammal, Indian E**

124. deBoer MN, Baldwin R, Burton CLK, Eyre EL, Jenner KCS, et al. (2002) Cetaceans in the Indian Ocean Sanctuary: A review. Wiltshire, UK: Whale and Dolphin Conservation Society. **marine mammal, Indian W, Indian E, Indian Antarctic**

125. Department of the Navy (1996) Request for letter of authorization for the incidental take of marine mammals associated with shock testing the SEAWOLF submarine. **marine mammal, sea turtle, Atlantic CW, Atlantic NW**

126. Department of the Navy (1996) Draft environmental impact statement for shock testing the SEAWOLF submarine. Prepared for the Southern Division, Naval Facilities Engineering Command by Continental Shelf Associates, Inc. **marine mammal, sea turtle, Atlantic CW, Atlantic NW**

127. Department of the Navy (1998) Shock testing the SEAWOLF submarine. Final environmental impact statement. North Charleston, SC: Department of the Navy, Naval Facilities Engineering Command. **marine mammal, Atlantic CW**

128. Department of the Navy (2001) Final environmental impact statement, shock trial of the USS Winston S. Churchill (DDG-81). Washington, DC: Naval Sea Systems Command. **marine mammal, Atlantic CW**

129. Department of the Navy (2001) Marine resources assessment for the Virginia Capes (VACAPES) operating area. Final report. Contract number N62470-95-D-1160, CTO 0030. Prepared by Geo-Marine, Inc., Plano, Texas. Norfolk, VA: Department of the Navy, Atlantic Division, Naval Facilities Engineering Command. **marine mammal, Atlantic CW**

130. Department of the Navy (2001) Final environmental impact statement: Shock trial of the Winston S. Churchill (DDG 81). Prepared for the Southern Division, Naval Facilities Engineering Command, North Charleston, SC. Washington, DC: Continental Shelf Associates, Inc. **marine mammal, Atlantic CW**

131. Department of the Navy (2002) Marine resources assessment for the Charleston/Jacksonville operating area. Final report. Contract number N62470-95-D-1160, CTO 0030. Prepared by Geo-Marine, Inc., Plano, Texas. Norfolk, VA: Department of the Navy, Atlantic Division, Naval Facilities Engineering Command. **marine mammal, Atlantic CW**

132. Department of the Navy (2002) Marine resources assessment for the Cherry Point operating area. Final report. Contract number N62470-95-D-1160, CTO 0030. Prepared by Geo-Marine, Inc., Plano, Texas. Norfolk, VA: Department of the Navy, Atlantic Division, Naval Facilities Engineering Command. **marine mammal, Atlantic CW**

133. Department of the Navy (2007) Marine resources assessment update for the Charleston/Jacksonville operating area. Draft report. Contract number N62470-02-D-9997, CTO 0056. Prepared by Geo-Marine, Inc., Plano, Texas. Norfolk, VA: Department of the Navy, Atlantic Division, Naval Facilities Engineering Command. **marine mammal, Atlantic CW**

134. Department of the Navy (2007) Marine resources assessment update for the Cherry Point operating area. Draft report. Contract number N62470-02-D-9997, CTO 0056. Prepared by Geo-Marine, Inc., Plano, Texas. Norfolk, VA: Department of the Navy, Atlantic Division, Naval Facilities Engineering Command. **marine mammal, Atlantic CW**

135. Department of the Navy (2007) Marine resources assessment update for the Virginia Capes (VACAPES) operating area. Draft report. Contract number N62470-02-D-9997, CTO 0056. Prepared by Geo-Marine, Inc., Plano, Texas. Norfolk, VA: Department of the Navy, Atlantic Division, Naval Facilities Engineering Command. **marine mammal, Atlantic CW**

136. Dias LA, Herzing D, Flach L (2009) Aggregations of Guiana dolphins (*Sotalia guianensis*) in Sepetiba Bay, Rio de Janeiro, south-eastern Brazil: Distribution patterns and ecological characteristics. J Mar Biol Assoc U K 89: 967-973. **marine mammal, Atlantic SW**

137. Dietz R, Heide-Jorgensen MP, Richard P, Orr J, Laidre K, et al. (2008) Movements of narwhals (*Monodon monoceros*) from Admiralty Inlet monitored by satellite telemetry. Polar Biol 31: 1295-1306. **marine mammal, Atlantic NW**

138. DiMarzio N, Moretti D, Ward J, Morrissey R, Jarvis S, et al. (2008) Passive acoustic measurement of dive vocal behavior and group size of Blainville's beaked whale (*Mesoplodon densirostris*) in the tongue of the ocean (TOTO). Can Acoust 36: 166-173. **marine mammal, Atlantic CW**

139. Dolar ML (2005) Cetaceans of American Samoa. Report to Department of Marine and Wildlife Resources. American Samoa: Department of Marine and Wildlife Resources, American Samoa. **marine mammal, Pacific CW, Pacific CE**

140. Dolman S, Claridge D, Durban J (2007) Monitoring abundance trends of Bahamian odontocetes. 17th biennial conference on the Biology of Marine Mammals (SMM), 29 November - 3 December 2007. Capetown, South Africa. **marine mammal, Atlantic CW**

141. Doroff A, Gorbics C (1998) Sea otter surveys of Yakutat Bay and adjacent Gulf of Alaska coastal areas—Cape Hinchinbrook to Cape Spencer 1995–1996. Final report to Minerals Management Service. Anchorage, AK: Minerals Management Service OCS Study MMS 97-0026. **marine mammal, Pacific NE**

142. Doroff AM, Estes JA, Tinker MT, Burn DM, Evans TJ (2003) Sea otter population declines in the Aleutian archipelago. J Mammal 84: 55-64. **marine mammal, Pacific NE**

143. Duck C (2003) Results of the thermal image survey of seals around the coast of northern Ireland. A report commissioned by the Environment and Heritage Service. Belfast: Environment and Heritage Service. **marine mammal, Atlantic NE**

144. Dueck L, Heide J, Mads P, Postma L, Jensen M (2007) Movements and seasonal distribution of bowhead whales in Canada's eastern Arctic: Implications for stock identity and management. 17th biennial conference on the Biology of Marine Mammals (SMM), 29 November - 3 December 2007. Capetown, South Africa. **marine mammal, Atlantic NW**

145. Dunphy-Daly MM, Heithaus MR, Claridge DE (2008) Temporal variation in dwarf sperm whale (*Kogia sima*) habitat use and group size off Great Abaco Island, Bahamas. Mar Mamm Sci 24: 171-182. **marine mammal, Atlantic CW**

146. Eckert SA, Moore JE, Dunn DC, van Buiten RS, Eckert KL, et al. (2008) Modeling loggerhead turtle movement in the Mediterranean: Importance of body size and oceanography. Ecol Appl 18: 290-308. **sea turtle, Mediterranean**

147. Elwen S, Best PB (2004) Environmental factors influencing the distribution of southern right whales (*Eubalaena australis*) on the south coast of South Africa II: Within bay distribution. Mar Mamm Sci 20: 583-601. **marine mammal, Atlantic SE**

148. Elwen S, Meÿer MA, Best PB, Kotze PGH, Thornton M, et al. (2006) Range and movements of female Heaviside’s dolphins (*Cephalorhynchus heavisidii*), as determined by satellite-linked telemetry. J Mammal 87: 866-877. **marine mammal, Atlantic SE**

149. Elwen SH, Best PB (2004) Environmental factors influencing the distribution of southern right whales (*Eubalaena australis*) on the south coast of South Africa I: Broad scale patterns. Mar Mamm Sci 20: 567-582. **marine mammal, Atlantic SE**

150. Etnoyer P, Canny D, Mate BR, Morgan LE, Ortega-Ortiz JG, et al. (2006) Sea-surface temperature gradients across blue whale and sea turtle foraging trajectories off the Baja California peninsula, Mexico. Deep Sea Res Part II 53: 340-358. **sea turtle, marine mammal, Pacific CE**

151. Evans PGH, Anderwald P (2005) Cetaceans in Liverpool Bay and northern Irish Sea: An update for the period of 2001-05. Oxford: Sea Watch Foundation. **marine mammal, Atlantic NE**

152. Eyre EJ (1995) Observations of cetaceans in the Indian Ocean Whale Sanctuary, May-July, 1993. Rep Int Whaling Comm 45: 419-426. **marine mammal, Indian W, Indian E, Indian Antarctic**

153. Eyre EJ (1997) Preliminary composite cetacean survey in the Indian Ocean Sanctuary, April 1995. Paper SC/49//O28 presented to the International Whaling Commission Scientific Committee. **marine mammal, Indian W, Indian E, Indian Antarctic**

154. Falcone EA, Schorr GS, Douglas AB, Calambokidis J, Henderson E, et al. (2009) Sighting characteristics and photo-identification of Cuvier's beaked whales (*Ziphius cavirostris*) near San Clemente Island, California: A key area for beaked whales and the military. Mar Biol. **marine mammal, Pacific CE**

155. Ferrero RC, Walker WA (1993) Growth and reproduction of the northern right whale dolphin, *Lissodelphis borealis*, in the offshore waters of the north Pacific Ocean. Can J Zool 71: 2335-2344. **marine mammal, Pacific NW, Pacific NE, Pacific CE**

156. Ferrero RC, Walker WA (1995) Growth and reproduction of the common dolphin, *Delphinus delphis Linnaeus*, in the offshore waters of the north Pacific Ocean. Fish Bull 93: 483-494. **marine mammal, Pacific NW, Pacific NE, Pacific CE**

157. Fertl D, Jefferson TA, Moreno IB, Zerbini AN, Mullin KD (2003) Distribution of the clymene dolphin *Stenella clymene*. Mammal Rev 33: 253-271. **marine mammal, Atlantic SW, Atlantic CW**

158. Fiedler PC, Reilly SB, Hewitt RP, Demer D, Philbrick VA, et al. (1998) Blue whale habitat and prey in the California Channel Islands. Deep Sea Res Part II 45: 1781-1801. **marine mammal, Pacific CE**

159. Forcada J, Aguilar A, Hammon P, Pastor X, Aguilar R (1996) Distribution and abundance of fin whales (*Balaenoptera physalus*) in the western Mediterranean Sea during the summer. J Zool 238: 23-34. **marine mammal, Mediterranean**

160. Forcada J, Aguilar A, Hammond PS, Pastor X, Aguilar R (1994) Distribution and numbers of striped dolphins in the western Mediterranean Sea after the 1990 epizootic outbreak. Mar Mamm Sci 10: 137-150. **marine mammal, Mediterranean**

161. Forcada J, Notarbartolo di Sciara G, Fabbri F (1995) Abundance of fin whales and striped dolphins summering in the Corso-Ligurian basin. Mammalia 59: 127-140. **marine mammal, Mediterranean**

162. Forney KA (1995) A decline in the abundance of harbor porpoise, *Phocoena phocoena*, in nearshore waters off California, 1986-93. Fish Bull 93: 741-748. **marine mammal, Pacific NE**

163. Forney KA (1999) Trends in harbor porpoise abundance off central California 1986-95: evidence for interannual changes in distribution? J Cetacean Res Manag 1: 73-80. **marine mammal, Pacific NE**

164. Forney KA, Barlow J (1998) Seasonal patterns in the abundance and distribution of California cetaceans, 1991-1992. Mar Mamm Sci 14: 460-489. **marine mammal, Pacific NE**

165. Forney KA, Barlow J, Carretta JV (1995) The abundance of cetaceans in California waters. Part 2. Aerial surveys in winter and spring of 1991 and 1992. Fish Bull 93: 15-26. **marine mammal, Pacific NE**

166. Forney KA, Brownell RL (1996) Preliminary report of the 1994 Aleutian Island marine mammal survey. Paper SC/48/O11 presented to the International Whaling Commission Scientific Committee. **marine mammal, Pacific NE**

167. Forney KA, Hanan DA, Barlow J (1991) Detecting trends in harbor porpoise abundance from aerial surveys using analysis of covariance. Fish Bull 89: 367-377. **marine mammal, Pacific NE**

168. Frankel AS, Clark CW (2000) Behavioral responses of humpback whales (*Megaptera novaeangliae*) to full-scale ATOC signals. J Acoust Soc Am 108: 1930-1937. **marine mammal, Pacific CE**

169. Frankel AS, Clark CW (2002) ATOC and other factors affecting the distribution and abundance of humpback whales (*Megaptera novaeangliae*) off the north shore of Kauai. Mar Mamm Sci 18: 644-662. **marine mammal, Pacific CE**

170. Frankel AS, Clark CW, Herman LM, Gabriele CM (1995) Spatial-distribution, habitat utilization, and social interactions of humpback whales, *Megaptera-novaeangliae*, off Hawaii, determined using acoustic and visual techniques. Mar Fish Rev 73: 1134-1146. **marine mammal, Pacific CE**

171. Frantzis A, Alexiadou P, Paximadis G, Politi E, Gannier A, et al. (2003) Current knowledge of the cetacean fauna of the Greek Seas. J Cetacean Res Manag 5: 219-232. **marine mammal, Mediterranean**

172. Frantzis A, Nikolaou O, Bompar JM, Cammedda A (2004) Humpback whale (*Megaptera novaeangliae*) occurrence in the Mediterranean Sea. J Cetacean Res Manag 6: 25-28. **marine mammal, Mediterranean**

173. Frantzis A, Swift R, Gillespie D, Menhennett C, Gordon J, et al. (1999) Sperm whale presence off south-west Crete, Greece, eastern Mediterranean. Eur Res Cetacean 13: 214-217. **marine mammal, Mediterranean**

174. Freitas C, Kovacs K, Ims R, Fedak M, Lydersen C (2008) Ringed seal post-moulting movement tactics and habitat selection. Oecologia 155: 193-204. **marine mammal, Atlantic NE**

175. Fritts TH, Hoffman W, McGehee MA (1983) The distribution and abundance of marine turtles in the Gulf of Mexico and nearby Atlantic waters. J Herpetol 17: 327-344. **sea turtle, Atlantic CW**

176. Fritts TH, Irvine AB, Jennings RD, Collum LA, Hoffman W, et al. (1983) Turtles, birds, and mammals in the northern Gulf of Mexico and nearby Atlantic waters. Washington, D.C.: U.S. Fish and Wildlife Service FWS/OBS-82/65. **marine mammal, sea turtle, seabird, Atlantic CW**

177. Fulling G, Cotton J, Rivers J, Thorson P (2007) Sei (*Balaenoptera borealis*) and Bryde's (*B. edeni/brydei*) whale co-occurrence in the Mariana Islands during the boreal winter. 17th biennial conference on the Biology of Marine Mammals (SMM), 29 November - 3 December 2007. Capetown, South Africa. **marine mammal, Pacific CW**

178. Fulling GL, Mullin KD, Hubard CW (2003) Abundance and distribution of cetaceans in outer continental shelf waters of the U.S. Gulf of Mexico. Fish Bull 101: 923-932. **marine mammal, Atlantic CW**

179. Fury CA, Harrison PL (2008) Abundance, site fidelity and range patterns of Indo-Pacific bottlenose dolphins (*Tursiops aduncus*) in two Australian subtropical estuaries. Mar Freshw Res 59: 13. **marine mammal, Pacific SW**

180. Gachal GS, Slater FM (2004) The capture and translocation of Indus river dolphins (*Platanista minor*) from the Sukkur barrage canals to the Indus River Dolphin Reserve, Sindh, Pakistan. Sind University Research Journal - Science Series 36: 1-10. **marine mammal, Indian W**

181. Gailey G, Wursig B, McDonald T (2007) Abundance, behavior, and movement patterns of western gray whales in relation to a 3-D seismic survey, northeast Sakhalin Island, Russia. Environ Monit Assess 134: 12. **Pacific NW, marine mammal**

182. Gallagher MD (1991) Collections of skulls of Cetacea: Odontoceti from Bahrain and the United Arab Emirates and Oman, 1969-1990. In: Leatherwood S, Donovan GP, editors. Cetaceans and cetacean research in the Indian Ocean Sanctuary, marine mammal technical report number 3. Nairobi, Kenya: United Nations Environment Program (UNEP). pp. 89-97. **marine mammal, Indian W, Indian E**

183. Galletti Vernazzani B, Carlson C, Cabrera E, Brownell Jr. RL (2006) Blue, sei and humpback whale sightings during 2006 field season in northwestern Isla de Chiloe, Chile. Paper SC/58/SH17 presented to the International Whaling Commission Scientific Committee. **marine mammal, Pacific SE**

184. Galletti Vernazzani B, Carlson CA, Cabrera E (2005) Blue whale sightings during the 2005 field season in northwestern Chiloe Island, Southern Chile. Paper SC/57/SH14 presented to the International Whaling Commission Scientific Committee. **marine mammal, Pacific SE**

185. Galletti Vernazzani B, Carlson CA, Cabrera E, Brownell Jr RL (2007) Status of blue whales off Isla de Chiloe, Chile from 2004-2007. 17th biennial conference on the Biology of Marine Mammals (SMM), 29 November - 3 December 2007. Capetown, South Africa. **marine mammal, Pacific SE**

186. Gallo-Reynoso J-P, Égido-Villarreal J, Coria-Galindo E-M (2009) Sperm whale distribution and diving behaviour in relation to presence of jumbo squid in Guaymas Basin, Mexico. JMBA2 Biodiversity Records 2. **marine mammal, Pacific CE**

187. Gannier A (1997) Estimation of summer abundance of the fin whale *Balaenoptera physalus* (Linne, 1758) in the Liguro-Provencal basin (west Mediterranean). Rev Ecol-Terre Vie 52: 69-86. **marine mammal, Mediterranean**

188. Gannier A (1998) Estimation of summer abundance of the striped dolphin *Stenella coeruleoalba* (Meyen, 1833) in the future northwestern Mediterranean international marine sanctuary. Rev Ecol-Terre Vie 53: 255-272. **marine mammal, Mediterranean**

189. Gannier A (1999) Comparison of the distribution of odontocetes obtained from visual and acoustic data in northwestern Mediterranean. Eur Res Cetacean 12: 246-250. **marine mammal, Mediterranean**

190. Gannier A (1999) Seasonal variation of the bathymetric distribution of cetaceans in the Liguro-Provencal basin (western Mediterranean). Vie Milieu 48: 25-34. **marine mammal, Mediterranean**

191. Gannier A (2000) Distribution of cetaceans off the Society Islands (French Polynesia) as obtained from dedicated surveys. Aquat Mamm 26: 111-126. **marine mammal, Pacific CE**

192. Gannier A (2002) Cetaceans of the Marquesas Islands (French Polynesia): Distribution and relative abundance as obtained from a small boat dedicated survey. Aquat Mamm 28: 198-210. **marine mammal, Pacific CE**

193. Gannier A (2005) Summer distribution and relative abundance of delphinids in the Mediterranean Sea. Rev Ecol-Terre Vie 60: 223-238. **marine mammal, Mediterranean**

194. Gannier A (2006) Summer cetacean population in the Pelagos Marine Sanctuary (northwest Mediterranean): Distribution and abundance. Mammalia 70: 17-27. **marine mammal, Mediterranean**

195. Gannier A, Drouot V, Goold JC (2002) Distribution and relative abundance of sperm whales in the Mediterranean Sea. Mar Ecol Prog Ser 243: 281-293. **marine mammal, Mediterranean**

196. Gannier A, Epinat J (2008) Cuvier's beaked whale distribution in the Mediterranean Sea: Results from small boat surveys 1996-2007. J Mar Biol Assoc U K 88: 1245-1251. **marine mammal, Mediterranean**

197. Gannier A, West KL (2005) Distribution of the rough-toothed dolphin (*Steno bredanensis*) around the Windward Islands (French Polynesia). Pac Sci 59: 17-24. **marine mammal, Pacific CE**

198. Gannier O, Gannier A (1999) First results on the distribution of cetaceans in the Society Islands (French Polynesia). Eur Res Cetacean 12: 54-58. **marine mammal, Pacific CE**

199. Garrison L, Yeung C (2001) Abundance estimates for Atlantic bottlenose dolphin stocks during summer and winter, 1995. Unpublished document prepared for the Take Reduction Team on coastal bottlenose dolphins in the western Atlantic. Miami, FL: NOAA-NMFS-SEFSC. **marine mammal, Atlantic NW, Atlantic CW**

200. Garrison LP, Rosel PE, Hohn A, Baird R, Hoggard W (2003) Abundance of the coastal morphotype of bottlenose dolphin, *Tursiops truncatus*, in U.S. continental shelf waters between New Jersey and Florida during winter and summer 2002. Unpublished document prepared for the Take Reduction Team on coastal bottlenose dolphins in the western Atlantic. Miami, FL: NOAA-NMFS-SEFSC. **marine mammal, Atlantic NW, Atlantic CW**

201. Garrison LP, Swartz SL, Martinez A, Burks C, Stamates J (2003) A marine mammal assessment survey of the southeast US continental shelf: February - April 2002. Miami, FL: NOAA Technical Memorandum NMFS-SEFSC-492. **marine mammal, Atlantic CW**

202. Gendron D, Lanham S, Carwardine M (1999) North Pacific right whale (*Eubalaena glacialis*) sighting south of Baja California. Aquat Mamm 25: 31-34. **marine mammal, Pacific CE**

203. Genov T, Kotnjek P (2009) New record of the humpback whale (*Megaptera novaeangliae*) in the Adriatic Sea. Ann Ser Hist Nat 19: 25-30. **marine mammal, Mediterranean**

204. Genov T, Kotnjek P, Lesjak J, Hace A, Fortuna CM (2008) Bottlenose dolphins (*Tursiops truncatus*) in Slovenian and adjacent waters (northern Adriatic Sea). Ann Ser Hist Nat 18: 227-244. **marine mammal, Mediterranean**

205. George JC, Clark C, Carroll GM, Ellison WT (1989) Observations on the ice-breaking and ice navigation behavior of migrating bowhead whales (*Balaena mysticetus*) near Point Barrow, Alaska, spring 1985. Arctic 42: 24-30. **marine mammal, Arctic**

206. George JC, Zeh J, Suydam R, Clark C (2004) Abundance and population trend (1978-2001) of western Arctic bowhead whales surveyed near Barrow, Alaska. Mar Mamm Sci 20: 755-773. **marine mammal, Arctic**

207. Gilg O, Born EW (2005) Recent sightings of the bowhead whale (*Balaena mysticetus*) in northeast Greenland and the Greenland Sea. Polar Biol 28: 796-801. **marine mammal, Atlantic NE**

208. Gill PC, Morrice MG, Jenner KJ, Gales N (2007) Occurrence, broad-scale feeding habitat and movements of blue whales in a south-east Australian coastal upwelling system. 17th biennial conference on the Biology of Marine Mammals (SMM), 29 November - 3 December 2007. Capetown, South Africa. **marine mammal, Pacific SW**

209. Gilpatrick JW, Jr., Perrin WF, Leatherwood S, Shiroma L (1987) Summary of distribution records of the spinner dolphin, *Stenella longirostris*, and the pantropical spotted dolphin, *S. attenuata*, from the western Pacific Ocean, Indian Ocean and Red Sea. La Jolla, CA: NOAA-TM-NMFS-SWFSC-89. **marine mammal, Indian W, Indian E, Pacific CW, Pacific NW**

210. Gjertz I (1991) Distribution of hooded seals in Svalbard Arctic ocean waters. Fauna Norv Ser B 12: 19-24. **marine mammal, Atlantic NE**

211. Gjertz I, Griffiths D, Krafft BA, Lydersen C, Wiig O (2001) Diving and haul-out patterns of walruses *Odobenus rosmarus* on Svalbard. Polar Biol 24: 314-319. **marine mammal, Atlantic NE**

212. Gjertz I, Henriksen G, Ritsland T, Wiig Y (1993) Observations of walruses along the Norwegian coast 1967-1992. Polar Res 12: 27-31. **marine mammal, Atlantic NE**

213. Gjertz I, Kovacs KM, Lydersen C, Wiig Ø (2000) Movements and diving of bearded seal (*Erignathus barbatus*) mothers and pups during lactation and post-weaning. Polar Biol 23: 559-566. **marine mammal, Atlantic NE**

214. Gjertz I, Kovacs KM, Lydersen C, Wiig Ø (2000) Movements and diving of adult ringed seals (*Phoca hispida*) in Svalbard. Polar Biol 23: 651-656. **marine mammal, Atlantic NE**

215. Gjertz I, Lydersen C, Wiig O (2001) Distribution and diving of harbour seals (*Phoca vitulina*) in Svalbard. Polar Biol 24: 209-214. **marine mammal, Atlantic NE**

216. Gjertz I, Wiig O (1994) Past and present distribution of walruses in Svalbard. Arctic 47: 34-42. **marine mammal, Atlantic NE**

217. Gjertz I, Wiig O (1994) Distribution and catch of white whales (*Delphinapterus leucas*) at Svalbard. Meddelelser om Gronland Bioscience 39: 93-97. **marine mammal, Atlantic NE**

218. Gjertz I, Wiig O (1995) The number of walruses (*Odobenus-rosmarus*) in Svalbard in summer. Polar Biol 15: 527-530. **marine mammal, Atlantic NE**

219. Gnone G, Nuti S, Bellingeri M, Pannoncini R, Bedocchi D (2006) Spatial behaviour of *Tursiops truncatus* along the Ligurian Sea coast: Preliminary results. Biol Mar Medit 13: 272-273. **marine mammal, Mediterranean**

220. Goddard PD, Rugh DJ (1998) A group of right whales seen in the Bering Sea in July 1996. Mar Mamm Sci 14: 344-349. **marine mammal, Pacific NW**

221. Goetz KT, Rugh DJ, Read AJ, Hobbs RC (2007) Habitat use in a marine ecosystem: Beluga whales *Delphinapterus leucas* in Cook Inlet, Alaska. Mar Ecol Prog Ser 330: 247-256. **marine mammal, Pacific NE**

222. Goff GP, Lien J (1988) Atlantic leatherback turtles in cold water off Newfoundland and Labrador. Can Field-Nat 102: 1-5. **sea turtle, Atlantic NW**

223. Goff GP, Lien J, Stenson GB, Fretey J (1994) The migration of a tagged leatherback turtle, *Dermochelys coriacea*, from French Guiana, South America to Newfoundland, Canada in 128 days. Can Field-Nat 108: 72-73. **sea turtle, Atlantic CW, Atlantic NW**

224. Goodman MA, Braun-McNeill J, Davenport E, Hohn AA (2007) Protected species aerial survey data collection and analysis in waters underlying the R-5306A airspace: Final report submitted to U.S. Marine Corps, MCAs Cherry Point. Beaufort, NC: NOAA Technical Memorandum NMFS-SEFSC-551. **sea turtle, Atlantic CW**

225. Gordon J, Scott-Hayward L, Donovan C, Richter C, Wursig B, et al. (2007) Distribution and habitat preferences of sperm whales in the northern Gulf of Mexico: Overlap with current and projected oil and gas activities and management implications (sperm whale seismic survey). 17th biennial conference on the Biology of Marine Mammals (SMM), 29 November - 3 December 2007. Capetown, South Africa. **marine mammal, Pacific CE**

226. Gosselin J-F, Hammill M, Lesage V (2007) Abundance of belugas in the St. Lawrence estuary, Canada: Photographic versus visual line transect survey methods. 17th biennial conference on the Biology of Marine Mammals (SMM), 29 November - 3 December 2007. Capetown, South Africa. **marine mammal, Atlantic NW**

227. Griffin RB (1999) Sperm whale distributions and community ecology associated with a warm-core ring off Georges Bank. Mar Mamm Sci 15: 33-51. **marine mammal, Atlantic NW**

228. Grigione MM (1991) Status and distribution of manatees in Cameroon. Sirenews: 7-9. **marine mammal, Atlantic CE**

229. Grigione MM (1996) Observations on the status and distribution of the west African manatee in Cameroon. Afr J Ecol 34: 189-195. **marine mammal, Atlantic CE**

230. Grønvik S (2005) Norway. Progress report on cetacean research, January 2004 to December 204, with statistical data for the calendar year 2004. Tromsø, Norway: Norwegian Institute for Nature Research, The Polar Environmental Center SC/57/ProgRepNorway. **marine mammal, Atlantic NE**

231. Güçlüsoy H (2008) Interaction between monk seals, *Monachus monachus* (Hermann, 1779), and artisanal fisheries in the Foca Pilot Monk Seal Conservation Area, Turkey. Zool Middle East 43: 13-20. **marine mammal, Mediterranean**

232. Güçlüsoy H, Savaş Y (2003) Status of the Mediterranean monk seal, *Monachus monachus*, in the Foca Pilot Monk Seal Conservation Area, Turkey. Zool Middle East 28: 5-16. **marine mammal, Mediterranean**

233. Gücü AC, Ok M, Sakinan S (2009) A survey of the critically endangered Mediterranean monk seal, *Monachus monachus* (Hermann, 1779) along the coast of northern Cyprus. Isr J Ecol Evol 55: 77-82. **marine mammal, Mediterranean**

234. Gücü AC, Sakinan S, Ok M (2009) Occurrence of the critically endangered Mediterranean monk seal, *Monachus monachus*, at Olympos-Beydaglari National Park, Turkey (Mammalia: Phocidae). Zool Middle East 46: 3-8. **marine mammal, Mediterranean**

235. Guilbault Y, Blouin J-F, Brown M, de la Cheneliere V, Giard J (2007) The Gulf of St. Lawrence: Another nursery for western North Atlantic right whales? 17th biennial conference on the Biology of Marine Mammals (SMM), 29 November - 3 December 2007. Capetown, South Africa. **marine mammal, Atlantic NW**

236. Gunnlaugsson T, Sigurónsson J (1990) NASS-87: Estimation of whale abundance on observations made onboard Icelandic and Faeroese survey vessels. Rep Int Whaling Comm 40: 571-580. **marine mammal, Atlantic NE**

237. Hain JHW, Hyman MAM, Kenney RD, Winn HE (1985) The role of cetaceans in the shelf-edge region of the northeastern United States. Mar Fish Rev 47: 13-17. **marine mammal, Atlantic NW**

238. Hain JHW, Ratnaswamy MJ, Kenney RD, Winn HE (1992) The fin whale, *Balaenoptera physalus*, in waters of the northeastern United States continental shelf. Rep Int Whaling Comm 42: 653-669. **marine mammal, Atlantic NW**

239. Hamazaki T (2002) Spatiotemporal prediction models of cetacean habitats in the mid-western North Atlantic Ocean (from Cape Hatteras, North Carolina, USA to Nova Scotia, Canada). Mar Mamm Sci 18: 920-939. **marine mammal, Atlantic CW, Atlantic NW**

240. Hammond P, Berggren P, Borchers D, Burt L, Canadas A, et al. (2007) Abundance of harbour porpoise and other small cetaceans in the European Atlantic and North Sea. 17th biennial conference on the Biology of Marine Mammals (SMM), 29 November - 3 December 2007. Capetown, South Africa. **marine mammal, Atlantic NE**

241. Hansen DJ, Hubbard JD (1998) Distribution and abundance of Cook Inlet beluga whales (*Delphinapterus leucas*) in winter. Draft final report. Anchorage, AK: U.S. Department of the Interior Bureau of Land Management, Minerals Management Service Environmental Studies Section, Alaska OCS Reg. **marine mammal, Pacific NE**

242. Hansen LJ, Mullin KD, Roden CL (1994) Preliminary estimates of cetacean abundance in the northern Gulf of Mexico, and of selected cetacean species in the U.S. Atlantic exclusive economic zone from vessel surveys. Miami, FL: NOAA-NMFS-SEFSC Contribution No. MIA-93/94-58. **marine mammal, Atlantic CW**

243. Hanson MB, Noren DP, Norris TF, Emmons CK, Guy TJ, et al. (2008) Pacific Ocean killer whale and other cetaceans distribution survey, March 2006 (PODs 2006) conducted aboard the NOAA ship McArthur II. Unpublished final cruise report. Seattle, WA: NOAA-NMFS-NWFSC. **marine mammal, Pacific NE**

244. Härkönen T, Heide-Jorgensen M-P (1990) Density and distribution of the ringed seal in the Bothnian Bay. Holarct Ecol 13: 122-129. **marine mammal, Atlantic NE**

245. Härkönen T, Jüssi M, Baimukanov M, Bignert A, Dmitrieva L, et al. (2008) Pup production and breeding distribution of the Caspian seal (*Phoca caspica*) in relation to human impacts. Ambio 37: 356-361. **marine mammal, Mediterranean**

246. Härkönen T, Jüssi M, Baimukanov M, Dmitrieva L, Kasimbekov Y, et al. (2005) Population size and density distribution of the Caspian seal (*Phoca caspica*) on the winter ice field in Kazakh waters, 2005. Final report prepared for the Caspian Environment Programme. Caspian International Seal Survey (CISS). **marine mammal, Mediterranean**

247. Härkönen T, Lunneryd SG (1992) Estimating abundance of ringed seals in the Bothnian Bay. Ambio 21. **marine mammal, Atlantic NE**

248. Harwood LA, Innes S, Norton P, Kingsley MCS (1996) Distribution and abundance of beluga whales in the Mackenzie estuary, southeast Beaufort Sea, and west Amundsen Gulf during late July 1992. Can J Fish Aquat Sci 53: 2262-2273. **marine mammal, Arctic**

249. Hattori K, Yamamura O (2007) Distribution of Steller sea lions in the Nemuro Strait: Comparing with off the Japan coast of Hokkaido Island. 17th biennial conference on the Biology of Marine Mammals (SMM), 29 November - 3 December 2007. Capetown, South Africa. **marine mammal, Pacific NW**

250. Hayes FE, Baker WS (1989) Seabird distribution at sea in the Galapagos Islands - environmental correlations and associations with upwelled water. Colonial Waterbirds 12: 60-66. **seabird, Pacific SE**

251. Heide-Jørgensen MP, Borchers DL, Witting L, Laidre KL, Simon MJ, et al. (2008) Estimates of large whale abundance in west Greenland waters from an aerial survey in 2005. J Cetacean Res Manag 10: 119-129. **marine mammal, Atlantic NW**

252. Heide-Jørgensen MP, Borchers DL, Witting L, Simon MJ, Laidre KL, et al. (2007) Final estimates of large whale abundance in west Greenland waters from an aerial survey in 2005. Paper SC/86/AWMP7 presented to the International Whaling Commission Scientific Committee. **marine mammal, Atlantic NW**

253. Heide-Jørgensen MP, Dietz R, Laidre KL, Richard P, Orr J, et al. (2003) The migratory behaviour of narwhals (*Monodon monoceros*). Can J Zool 81: 1298-1305. **marine mammal, Atlantic NW**

254. Henriksen G, Kondakov A (1997) A review of the distribution and abundance of harbor seals, *Phoca vitulina*, on Svalbard, Norway, and in the Barents Sea. Mar Mamm Sci 13: 157-163. **marine mammal, Atlantic NE**

255. Hermans A, Pistorius PA (2008) Marine mammal diversity in the remote waters of Aldabra Atoll, southern Seychelles. Atoll Res Bull 564. **marine mammal, Indian W**

256. Higdon J, Ferguson S (2007) Sea ice declines and increasing killer whale sightings in Hudson Bay, Canada. 17th biennial conference on the Biology of Marine Mammals (SMM), 29 November - 3 December 2007. Capetown, South Africa. **marine mammal, Atlantic NW**

257. Hobbs RC, Laidre KL, Vos DJ, Mahoney BA, Eagleton M (2005) Movements and area use of belugas, *Delphinapterus leucas*, in a subarctic Alaskan estuary. Arctic 58: 331-340. **marine mammal, Pacific NE**

258. Hobbs RC, Rugh DJ, DeMaster DP (2000) Abundance of beluga whales, *Delphinapterus leucas*, in Cook Inlet, Alaska, 1994-2000. Mar Fish Rev 62: 37-45. **marine mammal, Pacific NE**

259. Hobbs RC, Shelden KEW (2008) Supplemental status review and extinction assessment of Cook Inlet belugas (*Delphinapterus leucas*). NOAA AFSC Processed Report 2008-08. Seattle, WA: NOAA-NMFS-AFSC. **marine mammal, Pacific NE**

260. Hobbs RC, Shelden KEW, Rugh DJ, Norman SA (2008) 2008 status review and extinction risk assessment of Cook Inlet belugas (*Delphinapterus leucas*). Seattle, WA: NOAA-NMFS-AFSC Processed Reports 2008-02. **marine mammal, Pacific NE**

261. Hobbs RC, Waite JM, Rugh DJ (2000) Beluga, *Delphinapterus leucas*, group sizes in Cook Inlet, Alaska, based on observer counts and aerial video. Mar Fish Rev 62: 46-59. **marine mammal, Pacific NE**

262. Horrocks JA, Vermeer LA, Krueger B, Coyne M, Schroeder BA, et al. (2001) Migration routes and destination characteristics of post-nesting hawksbill turtles satellite-tracked from Barbados, West Indies. Chelonian Conserv Biol 4: 107-114. **sea turtle, Atlantic CW**

263. Hubbard JD, Hansen DJ, Mahoney BA (1999) Winter sighting of beluga whales (*Delphinapterus leucas*) in Yakutat-Disenchantment Bay, Alaska. Arctic 52: 411-412. **marine mammal, Pacific NE**

264. Hucke-Gaete R, Osman LP, Moreno CA, Findlay KP, Ljungblad DK (2003) Discovery of a blue whale feeding and nursing ground in southern Chile. Proc R Soc B: Biol Sci 271: S170-S173. **marine mammal, Pacific SE**

265. Huettmann F (1998) Seabird surveys and selected environmental data sets in the Bay of Fundy: Findings and conclusions from monthly ferry transects Saint John-Digby-Saint John. In: Burt MDB, Wells PG, editors. Coastal monitoring and the Bay of Fundy Proceedings of the Maritime Atlantic Ecozone Science Workshop held in St Andrews, New Brunswick: Huntsman Marine Science Centre. pp. 85-92. **seabirds, Atlantic NW**

266. Hunt GL (1997) Physics, zooplankton, and the distribution of least auklets in the Bering Sea - a review. ICES J Mar Sci 54: 600-607. **seabird, Pacific NE**

267. Hunt J, G.L., Kaiwi J, Schneider D (1981) Pelagic distribution of marine birds and analysis of encounter probability for the southwestern Bering Sea: Final report. Boulder, CO: Outer Continental Shelf Environmental Assessment Program and NOAA, Environmental Research Laboratories 16. **seabird, Arctic**

268. Hyrenbach KD (2001) Albatross response to survey vessels: Implications for studies of the distribution, abundance, and prey consumption of seabird populations. Mar Ecol Prog Ser 212: 283-295. **seabird, Pacific CE**

269. Hyvärinen H, Hämäläinen E, Kunnasranta M (1995) Diving behavior of the Saimaa ringed seal (*Phoca hispida saimensis*) Nordq. Mar Mamm Sci 11: 324-334. **marine mammal, Atlantic NE**

270. Iwasaki T, Hwang H, Nishiwaki S (1995) Report of whale sighting surveys in waters off Korean Peninsula and adjacent waters in 1994. Paper SC/47/NP18 presented to the International Whaling Commission Scientific Committee. **marine mammal, Pacific NW**

271. Iwasaki T, Shimada H, Kim ST (2000) Cruise report of Japanese pilot sighting survey conducted under the joint research program between Japan and Republic of Korea in 1999 summer. Paper SC/52/RMP 11 presented to the International Whaling Commission Scientific Committee. **marine mammal, Pacific NW**

272. Jaquet N, Whitehead H (1996) Scale-dependent correlation of sperm whale distribution with environmental features and productivity in the South Pacific. Mar Ecol Prog Ser 135: 1-9. **marine mammal, Pacific SW, Pacific CE, Pacific SE**

273. Jaquet N, Whitehead H, Lewis M (1996) Coherence between 19th century sperm whale distributions and satellite derived pigments in the tropical Pacific. Mar Ecol Prog Ser 145: 1-10. **marine mammal, Pacific CE, Pacific SW, Pacific SW, Pacific NW, Pacific CW**

274. Jefferson TA (1991) Observations on the distribution and behaviour of Dall's porpoise (*Phocoenoides dalli*) in Monterey Bay, California. Aquat Mamm 17: 12-19. **marine mammal, Pacific CE**

275. Jefferson TA (2000) Population biology of the Indo-Pacific hump-backed dolphin in Hong Kong waters. Wildl Monogr 144: 1-65. **marine mammal, Pacific NW**

276. Jefferson TA, Leatherwood S (1997) Distribution and abundance of Indo-Pacific hump-backed dolphins (*Sousa chinensis* Osbeck, 1765) in Hong Kong waters. Asian Mar Biol 14: 93-110. **marine mammal, Pacific NW**

277. Jefferson TA, Lynn SK (1994) Marine mammal sightings in the Caribbean Sea and Gulf of Mexico, summer 1991. Caribb J Sci 30: 83-89. **marine mammal, Atlantic CW**

278. Jefferson TA, Schiro AJ (1997) Distribution of cetaceans in the offshore Gulf of Mexico. Mammal Rev 27: 27-50. **marine mammal, Atlantic CW**

279. Joyce GG, Desportes G, Bloch D (1990) The Faroese NASS-89 sightings cruise. Paper SC/42/011 presented to the International Whaling Commission Scientific Committee. **marine mammal, Atlantic NE**

280. Jung JL, Stephan E, Louis M, Alfonsi E, Liret C, et al. (2009) Harbour porpoises (*Phocoena phocoena*) in north-western France: Aerial survey, opportunistic sightings and strandings monitoring. J Mar Biol Assoc U K 89: 1045-1050. **marine mammal, Atlantic NE**

281. Kahn B (2001) Komodo National Park cetacean surveys: A rapid ecological assessment of cetacean diversity, abundance and distribution. Interim report. The Nature Conservancy, Indonesia Coastal and Marine Program. **marine mammal, Pacific CW**

282. Kahn B, James Y, Pet J (2000) Komodo National Park cetacean surveys: A rapid ecological assessment of cetacean diversity, distribution and abundance. J Pesisir Lautan August 3: 41-59. **marine mammal, Pacific CW**

283. Kaiya Z, Leatherwood S, Jefferson TA (1995) Records of small cetaceans in Chinese waters: A review. Asian Mar Biol 12: 119-139. **marine mammal, Pacific CW, Pacific NW**

284. Kasuya T, Wada S (1991) Distribution of large cetaceans in the Indian Ocean: Data from Japanese sightings records, November-March. In: Leatherwood S, Donovan GP, editors. Cetaceans and cetacean research in the Indian Ocean Sanctuary, marine mammal technical report number 3. Nairobi, Kenya: United Nations Environment Program (UNEP). pp. 139-170. **marine mammal, Indian W**

285. Katona SK, Beard JA, Girton PE, Wenzel F (1988) Killer whales (*Orcinus orca*) from the Bay of Fundy to the Equator, including the Gulf of Mexico. Rit Fiskid XI: 205-224. **marine mammal, Atlantic NW, Atlantic CW**

286. Keller CA, Ward-Geiger LI, Brooks WB, Slay CK, Taylor CR, et al. (2006) North Atlantic right whale distribution in relation to sea-surface temperature in the southeastern United States calving grounds. Mar Mamm Sci 22: 426-445. **marine mammal, Atlantic CW**

287. Kelly BP, Wartzok D (1996) Ringed seal diving behavior in the breeding season. Can J Zool 74: 1547-1555. **marine mammal, Atlantic NW**

288. Kemper C, Middleton J, Van Ruth P (2007) Oceanographic influences on the distribution of the pygmy right whale *Caperea marginata* off Australia and New Zealand. 17th biennial conference on the Biology of Marine Mammals (SMM), 29 November - 3 December 2007. Capetown, South Africa. **marine mammal, Pacific SW, Indian E**

289. Kemper CM, Mole J, Warneke RM, Ling JK, Needham DJ, et al. (1997) Southern right whales in southeastern Australia: Aerial surveys during 1991-93 and incidental information from 1904; Hindell M, Kemper C, editors: Surrey Beatty and Sons Pty Ltd. **marine mammal, Indian E**

290. Kenney RD (2001) The North Atlantic Right Whale Consortium databases. Maritimes 43: 3-5. **marine mammal, Atlantic NW, Atlantic CW**

291. Kenney RD, Hyman MAM, Winn HE (1985) Calculation of standing stocks and energetic requirements of the cetaceans of the northeast United States outer continental shelf. Woods Hole, MA: NOAA Technical Memorandum NMFS-F/NEC-41. **marine mammal, Atlantic NW**

292. Kenney RD, Winn HE (1986) Cetacean high-use habitats of the northeast United States continental shelf. Fish Bull 84: 345-357. **marine mammal, Atlantic NW, Atlantic CW**

293. Kenney RD, Winn HE (1987) Cetacean biomass densities near submarine canyons compared to adjacent shelf slope areas. Cont Shelf Res 7: 107-114. **marine mammal, Atlantic NW**

294. Kenney RD, Winn HE, Macaulay MC (1995) Cetaceans in the Great South Channel, 1979-1989: Right whale (*Eubalaena glacialis*). Cont Shelf Res 15: 385-414. **marine mammal, Atlantic NW**

295. Kim ZG, Miyashita T, Baik CI, Sohn H, Choi KH (2000) Report of Korean whale sighting survey conducted under Korea and Japan joint pilot research plan in summer 1999. Paper SC/52/RMP21 submitted to the International Whaling Commission Scientific Committee. **marine mammal, Pacific NW**

296. Kim ZG, Miyashita T, Baik CI, Sohn H, Choi KH (2000) Report of Korean whale sighting survey conducted under Korea and Japan joint pilot research plan in summer 1999. Paper SC/52/RMP21 presented to the International Whaling Commission Scientific Committee. **marine mammal, Pacific NW**

297. Kim ZG, Sohn H (2001) Cruise report of whale sighting survey in the East Sea of Korea, September - October 2000. Paper SC/53/RMP23 presented to the International Whaling Commission Scientific Committee. **marine mammal, Pacific NW**

298. King GL (2006) Review of marine turtle records in northern Ireland. Belfast: Environment and Heritage Service. **sea turtle, Atlantic NE**

299. Kingsley MCS (1996) Population index estimate for the belugas of the St. Lawrence in 1995. Can Tech Rep Fish Aquat Sci 2117: 1-38. **marine mammal, Atlantic NW**

300. Kingsley MCS (2000) Numbers and distribution of beluga whales, *Delphinapterus leucas*, in James Bay, eastern Hudson Bay, and Ungava Bay in Canada during the summer of 1993. Fish Bull 98: 736-747. **marine mammal, Atlantic NW**

301. Kingsley MCS, Reeves RR (1998) Aerial surveys of cetaceans in the Gulf of St. Lawrence in 1995 and 1996. Can J Zool 76: 1529-1550. **marine mammal, Atlantic NW**

302. Kiszka J, Gross A, Richard P, Ridoux V (2007) Habitat and resource partitioning among a community of top predators in the Mozambique Channel: A case study on tropical dolphins. 17th biennial conference on the Biology of Marine Mammals (SMM), 29 November - 3 December 2007. Capetown, South Africa. **marine mammal, Indian W**

303. Kiszka J, Macleod K, Van Canneyt O, Walker D, Ridoux V (2007) Distribution, encounter rates, and habitat characteristics of toothed cetaceans in the Bay of Biscay and adjacent waters from platform-of-opportunity data. ICES J Mar Sci: fsm067. **marine mammal, Atlantic NE**

304. Kobayashi DR, Polovina JJ, Parker DM, Kamezaki N, Cheng I, et al. (2008) Pelagic habitat characterization of loggerhead sea turtles, *Caretta caretta*, in the north Pacific Ocean (1997–2006): Insights from satellite tag tracking and remotely sensed data. J Exp Mar Biol Ecol 356: 19. **sea turtle, Pacific CE, Pacific CW**

305. Kooyman GL, Gentry RL (1978) Seal and emperor penguin survey, Victoria-land coast. Antarct J U S 13: 156-157. **seabird, marine mammal, Pacific Antarctic**

306. Kooyman GL, Hunke EC, Ackley SE, van Dam RP, Robertson G (2000) Moult of the emperor penguin: Travel, location, and habitat selection. Mar Ecol Prog Ser 204: 269-277. **seabird, Atlantic Antarctic**

307. Kooyman GL, Kooyman TG (1995) Diving behavior of emperor penguins nurturing chicks at Coulman Island, Antarctica. Condor 97: 536-549. **seabird, marine mammal, Atlantic Antarctic**

308. Kooyman GL, Kooyman TG, Horning M, Kooyman CA (1996) Penguin dispersal after fledging. Nature 383: 397-397. **seabird, Atlantic Antarctic**

309. Kooyman GL, Ponganis PJ (2007) The initial journey of juvenile emperor penguins. Aquat Conserv: Mar Freshwat Ecosyst 17: S37-S43. **seabird, Atlantic Antarctic**

310. Koski WR, Miller GW (2009) Habitat use by different size classes of bowhead whales in the central Beaufort Sea during late summer and autumn. Arctic 62: 137-150. **marine mammal, Arctic**

311. Krafft BA, Lydersen C, Gjertz I, Kovacs KM (2002) Diving behaviour of sub-adult harbour seals (*Phoca vitulina*) at Prins Karls Forland, Svalbard. Polar Biol 25: 230-234. **marine mammal, Atlantic NE**

312. Krafft BA, Lydersen C, Kovacs KM, Gjertz I, Haug T (2000) Diving behaviour of lactating bearded seals (*Erignathus barbatus*) in the Svalbard area. Can J Zool 78: 1408-1418. **marine mammal, Atlantic NE**

313. Kraus SD, Kenney RD, Knowlton AR, Ciano JN (1993) Endangered right whales of the southwestern North Atlantic. Herndon, VA: Minerals Management Service OCS Study MMS 93-0024. **marine mammal, Atlantic CW**

314. Krutzikowsky GK, Mate BR (2000) Dive and surfacing characteristics of bowhead whales (*Balaena mysticetus*) in the Beaufort and Chukchi Seas. Can J Zool 78: 1182-1198. **marine mammal, Arctic**

315. Kunnasranta M (2001) Behavioural biology of two ringed seal (*Phoca hispida*) subspecies in the large European lakes Saimaa and Ladoga. Joensuu, Finland: University of Joensuu. **marine mammal, Atlantic NE**

316. Kunnasranta M, Hyvärinen H, Sipilä T, Medvedev N (2001) Breeding habitat and lair structure of the ringed seal (*Phoca hispida ladogensis*) in northern Lake Ladoga in Russia Polar Biol 24: 171-174. **marine mammal, Atlantic NE**

317. Kunnasranta M, Hyvärinen H, Sorjonen J (1996) Underwater vocalizations of Ladoga ringed seals (*Phoca hispida ladogensis* Nordq.) in summertime. Mar Mamm Sci 12: 611-618. **marine mammal, Atlantic NE**

318. Kushlan JA, Steinkamp MJ (2007) Seabird nesting and conservation in the northern Bahamas. Waterbirds 30: 613-623. **seabird, Atlantic CW**

319. Lacey C, Lewis T, Moscrop A (2005) Sightings made during visual surveys of the Mediterranean Sea in 2003 and 2004, including an unusual encounter with rough-toothed dolphins (*Steno bredanensis*) in the Ionian Sea. Eur Res Cetacean 19: 113. **marine mammal, sea turtle, Mediterranean**

320. Lagerquist BA, Mate BR, Ortega-Ortiz JG, Winsor M, Urban-Ramirez J (2008) Migratory movements and surfacing rates of humpback whales (*Megaptera novaeangliae*) satellite tagged at Socorro Island, Mexico. Mar Mamm Sci 24: 815-830. **marine mammal, Pacific CE**

321. Lagerquist BA, Stafford KM, Mate BR (2000) Dive characteristics of satellite-monitored blue whales (*Balaenoptera musculus*) off the central California coast. Mar Mamm Sci 16: 375-391. **marine mammal, Pacific CE**

322. Laidre KL (2003) Space-use patterns of narwhals (*Monodon monoceros*) in the high Arctic. Seattle, WA: University of Washington. **marine mammal, Atlantic NW, Arctic**

323. Laidre KL, Heide-Jorgensen MP, Dietz R (2002) Diving behaviour of narwhals (*Monodon monoceros*) at two coastal localities in the Canadian high Arctic. Can J Zool 80: 624-635. **marine mammal, Atlantic NW**

324. Laidre KL, Jameson RJ, Gurarie E, Jeffries SJ, Allen H (2009) Spatial habitat use patterns of sea otters in coastal Washington. J Mammal 90: 906-917. **marine mammal, Pacific NE**

325. Laidre KL, Mizroch SA (1997) Database modifications and data analysis for the platforms of opportunity program at the National Marine Mammal Laboratory, 1958–1995. Unpublished report. Seattle, WA: NOAA-NMFS-AFSC-NMML. **marine mammal, Pacific NE**

326. Laidre KL, Shelden KE, Rugh DJ, Mahoney BA (2000) Beluga, *Delphinapterus leucas*, distribution and survey effort in the Gulf of Alaska. Mar Fish Rev 62: 27-36. **marine mammal, Pacific NE**

327. Laidre KL, Shelden KEW, Rugh DJ, Mahoney BA (2000) Distribution of beluga whales and survey effort in the Gulf of Alaska. Mar Fish Rev 62: 27-36. **marine mammal, Pacific NE**

328. Lander ME, Loughlin TR, Logsdon MG, VanBlaricom GR, Fadely BS, et al. (2009) Regional differences in the spatial and temporal heterogeneity of oceanographic habitat used by Steller sea lions. Ecol Appl 19: 1645-1659. **marine mammal, Pacific NE**

329. Landino SW, Treacy SD, Zerwick SA, Dunlap JB (1994) A large aggregation of bowhead whales (*Balaena mystivetus*) feeding near Point-Barrow, Alaska, in late October 1992. Arctic 47: 232-235. **marine mammal, Arctic**

330. Laran S, Drouot-Dulau V (2007) Seasonal variation of striped dolphins, fin- and sperm whales' abundance in the Ligurian Sea (Mediterranean Sea). J Mar Biol Assoc U K 87: 345-352. **marine mammal, Mediterranean**

331. Laran S, Gannier A (2004) Distribution of cetaceans in the Marquesas Islands (French Polynesia). Eur Res Cetacean 15: 426-430. **marine mammal, Pacific CE**

332. Lawson J, Gosselin J-F (2007) The first large-scale aerial survey of the Canadian east coast; a component of the TNASS. 17th biennial conference on the Biology of Marine Mammals (SMM), 29 November - 3 December 2007. Capetown, South Africa. **marine mammal, sea turtle, Atlantic NW**

333. Leatherwood S, Bowles AE, Reeves RR (1983) Aerial surveys of marine mammals in the southeastern Bering Sea. Final report to the Outer Continental Shelf Environmental Assessment Program Research Unit. NOAA and OCSEAP Final Report 42: 147-490. **marine mammal, Pacific NE**

334. Leatherwood S, Jefferson TA, J.C. Norris, Stevens WE, Hansen LJ, et al. (1993) Occurrence and sounds of Fraser's dolphins (*Lagenodelphis hosei*) in the Gulf of Mexico. Tex J Sci 45: 349-354. **marine mammal, Atlantic CW**

335. Leatherwood S, McDonald D, Prematunga WP, Girton P, Ilangakoon A, et al. (1991) Records of “Blackfish” (killer, false killer, pilot, pygmy killer and melon-headed whales) in the Indian Ocean, 1772-1986. In: Leatherwood S, Donovan GP, editors. Cetaceans and cetacean research in the Indian Ocean Sanctuary, marine mammal technical report number 3. Nairobi, Kenya: United Nations Environment Program (UNEP). pp. 33-65. **marine mammal, Indian W, Indian E**

336. Lee DS (1986) Marine mammals off the North Carolina coast with particular reference to possible impact of Proposed Empress II. Final report to the Department of the Navy, Naval Sea Systems Command. Contract N00024-85-M-B547. Washington, DC. **marine mammal, Atlantic CW**

337. Lee K, Bain H, Hurley GV (2005) Acoustic monitoring and marine mammal surveys in the Gully and Outer Scotian shelf before and during active seismic programs. Fisheries and Oceans Canada Center for Offshore Oil and Gas Environmental Research (COOGER). **marine mammal, Atlantic NW**

338. Lehnert L, Scheidat M, Williams R, Kock K-H (2007) Observations of killer whales from helicopter surveys in Antarctic Peninsula waters. 17th biennial conference on the Biology of Marine Mammals (SMM), 29 November - 3 December 2007. Capetown, South Africa. **marine mammal, Atlantic Antarctic**

339. Lewison RL, Crowder LB, Freeman S (2004) Quantifying the effects of fisheries on threatened species: The impact of pelagic longlines on loggerhead and leatherback sea turtles. Ecol Lett 7: 221-231. **sea turtle, Atlantic CW, Atlantic NW**

340. Liew HC (2002) Status of marine turtle conservation and research in Malaysia. Honolulu, HI: Western Pacific Regional Fishery Management Council. **sea turtle, Pacific CW**

341. Limpus CJ (2007) A biological review of Australian marine turtles. 5. Flatback turtle *Natator depressus* (Garman). Queensland Government Environmental Protection Agency, Queensland Parks and Wildlife Service. **sea turtle, Pacific CW**

342. Limpus CJ, Miller JD, Paramenter CJ, Reimer D, McLachlan N, et al. (1992) Migration of green (*Chelonia mydas*) and loggerhead (*Caretta caretta*) turtles to and from eastern Australian rookeries. Wildl Res 19: 347-358. **sea turtle, Pacific CW**

343. Limpus CJ, Mortimer J, Pilcher NJ (Unpublished) Marine turtles of the Indian Ocean and southeast Asian region: Breeding distribution, migration, and population trends. Manila, Philippines: Department of Environment and Natural Resources. **sea turtle, Indian W, Indian E**

344. Littaye A, Gannier A, Laran S, Wilson JPF (2004) The relationship between summer aggregation of fin whales and satellite-derived environmental conditions in the northwestern Mediterranean Sea. Remote Sens Environ 90: 44-52. **marine mammal, Mediterranean**

345. Ljungblad D, Moore SE, Van Schoik DR (1983) Aerial surveys of endangered whales in the Beaufort eastern Chukchi, and northern Bering Seas, 1982: Final report to Minerals Management Service, Alaska OCS Region, U.S. Department of Interior. San Diego, CA: Naval Ocean Systems Center. **marine mammal, Arctic**

346. Ljungblad DK, Moore SE, Van Schoik DR (1986) Seasonal patterns of distribution, abundance, migration and behavior of the western Arctic stock of bowhead whales, *Balaena mysticetus*, in Alaskan Seas. Rep Int Whaling Comm Special Issue 8: 177–205. **marine mammal, Arctic**

347. Lonergan M, Duck CD, Thompson D, Mackey BL, Cunningham L, et al. (2007) Using sparse survey data to investigate the declining abundance of British harbour seals. J Zool 271: 261-269. **marine mammal, Atlantic NE**

348. Lopez-Mendilaharsu M, Bauza A, Laporta M, Caraccio MN, Lezama C, et al. (2003) Review and conservation of sea turtles in Uruguay. Final Report: British Petroleum Conservation Programme and National Fish and Wildlife Foundation: 109. **sea turtle, Atlantic SW**

349. Lowry LF, Frost KJ, Davis R, DeMaster DP, Suydam RS (1998) Movements and behavior of satellite-tagged spotted seals (*Phoca largha*) in the Bering and Chukchi Seas. Polar Biol 19: 221-230. **marine mammal, Arctic**

350. Lowry LF, Frost KJ, Zerbini A, DeMaster D, Reeves RR (2008) Trend in aerial counts of beluga or white whales (*Delphinapterus leucas*) in Bristol Bay, Alaska, 1993-2005. J Cetacean Res Manag 10: 201-207. **marine mammal, Pacific NE**

351. Lowry MS (1999) Counts of California sea lion (*Zalophus californianus*) pups from aerial color photographs and from the ground: A comparison of two methods. Mar Mamm Sci 15: 143-158. **marine mammal, Pacific NE**

352. Lowry MS (2002) Counts of northern elephant seals at rookeries in the Southern California Bight: 1981-2001. La Jolla, CA: NOAA Technical Memorandum NOAA-TM-NMFS-SWFSC-345. **marine mammal, Pacific NE**

353. Lowry MS, Forney KA (2005) Abundance and distribution of California sea lions (*Zalophus califomianus*) in central and northern California during 1998 and summer 1999. Fish Bull 103: 331-343. **marine mammal, Pacific NE**

354. Lowry MS, Perryman WL (1992) Aerial photographic census for California sea lion (*Zalophus californianus*) pups at San Miguel Island, California for 1987-1990 and San Nicolas Island, California for 1990. La Jolla, CA: NOAA-NMFS-SWFSC Administrative Report LJ-92-19. **marine mammal, Pacific NE**

355. Lowry MS, Perryman WL, Lynn MS, Westlake RL, Julian F (1996) Counts of northern elephant seals, *Mirounga angustirostris*, from large-format aerial photographs taken at rookeries in southern California during the breeding season. Fish Bull 94: 176-185. **marine mammal, Pacific CE**

356. Lunn NJ, Stirling I, Nowicki SN (1997) Distribution and abundance of ringed (*Phoca hispida*) and bearded seals (*Erignathus barbatus*) in western Hudson Bay. Can J Fish Aquat Sci 54: 914-921. **marine mammal, Atlantic NW**

357. Luque PL, Davis CG, Reid DG, Wang J, Pierce. GJ (2006) Opportunistic sightings of killer whales from Scottish pelagic trawlers fishing for mackerel and herring off north Scotland (UK) between 2000 and 2006. Aquat Living Resour 19: 403-410. **marine mammal, Atlantic NE**

358. Luschi P, Papi F, Liew HC, Chan EH, Bonadonna F (1996) Long-distance migration and homing after displacement in the green turtle (*Chelonia mydas*): A satellite tracking study. J Comp Physiol A Sens Neural Behav Physiol 178: 447-452. **Sea turtle, Atlantic NW**

359. Lydersen C, Aars J, Kovacs KM (2007) Estimating the number of walruses in Svalbard, Norway, based on aerial surveys and satellite telemetry. 17th biennial conference on the Biology of Marine Mammals (SMM), 29 November - 3 December 2007. Capetown, South Africa. **marine mammal, Atlantic NE**

360. Lydersen C, Martin AR, Gjertz I, Kovacs KM (2007) Satellite tracking and diving behaviour of sub-adult narwhals (*Monodon monoceros*) in Svalbard, Norway. Polar Biol 30: 437-442. **marine mammal, Atlantic NE**

361. Lydersen C, Martin AR, Kovacs KM, Gjertz I (2001) Summer and autumn movements of white whales *Delphinapterus leucas* in Svalbard, Norway. Mar Ecol Prog Ser 219: 265-274. **marine mammal, Atlantic NE**

362. MacLeod C, Perrin WF, Pitman R, Barlow J, Ballance L, et al. (2006) Known and inferred distributions of beaked whale species (Cetacea: Ziphiidae). J Cetacean Res Manag 7: 271-286. **marine mammal, Pacific CE, Pacific NE, Pacific Antarctic, Atlantic Antarctic, Atlantic NW, Atlantic CW, Indian Antarctic, Pacific SE, Arctic, Atlantic NE**

363. MacLeod CD, Bennett E (2007) Pan-tropical spotted dolphins (*Stenella attenuata*) and other cetaceans around St. Helena in the tropical south-eastern Atlantic. J Mar Biol Assoc U K 87: 339-344. **marine mammal, Atlantic SE**

364. MacLeod CD, Brereton T, Martin C (2009) Changes in the occurrence of common dolphins, striped dolphins and harbour porpoises in the English Channel and Bay of Biscay. J Mar Biol Assoc U K 89: 1059-1065. **marine mammal, Atlantic NE**

365. MacLeod CD, Hauser N, Peckham H (2004) Diversity, relative density and structure of the cetacean community in summer months east of Great Abaco, Bahamas. J Mar Biol Assoc U K 84: 469-474. **marine mammal, Atlantic CW**

366. MacLeod CD, Weir CR, Pierpoint C, Harland EJ (2007) The habitat preferences of marine mammals west of Scotland (UK). J Mar Biol Assoc U K 87: 157-164. **marine mammal, Atlantic NE**

367. MacLeod CD, Weir CR, Santos MB, Dunn TE (2008) Temperature-based summer habitat partitioning between white-beaked and common dolphins around the United Kingdom and Republic of Ireland. J Mar Biol Assoc U K 88: 1193-1198. **marine mammal, Atlantic NE**

368. MacLeod CD, Zuur AF (2005) Habitat utilization by Blainville's beaked whales off Great Abaco, northern Bahamas, in relation to seabed topography. Mar Biol 147: 1-11. **marine mammal, Atlantic CW**

369. Macleod K, Fairbairns R, Gill A, Fairbairns B, Gordon J, et al. (2004) Seasonal distribution of minke whales *Balaenoptera acutorostrata* in relation to physiography and prey off the Isle of Mull, Scotland. Mar Ecol Prog Ser 277: 263-274. **marine mammal, Atlantic NE**

370. Macleod K, Simmonds MP, Murray E (2003) Summer distribution and relative abundance of cetacean populations off north-west Scotland. J Mar Biol Assoc U K 83: 1187-1192. **marine mammal, Atlantic NE**

371. Macleod K, Simmonds MP, Murray E (2006) Abundance of fin (*Balaenoptera physalus*) and sei whales (*B. borealis*) amid oil exploration and development off northwest Scotland. J Cetacean Res Manag 8: 247-254. **marine mammal, Atlantic NE**

372. Magalhães FA, Garri RG, Tosi CH, Siciliano S, Castro ACL, et al. (2007) First confirmed record of *Feresa attenuata* (Delphinidae) for the northern Brazilian coast. Biota Neotrop 7: 1-3. **marine mammal, Atlantic SW**

373. Magalhães FA, Sever MM, Tosi CH, Garri RG, Zerbini A, et al. (2007) Record of a dwarf minke whale (*Balaenoptera acutorostrata*) in northern Brazil. JMBA2 Biodiversity Records 1: 1/5600-5602 **marine mammal, Atlantic SW**

374. Magalhães FA, Tosi CH, Garri RG, Chellappa S, Silva FL (2008) Cetacean diversity on the Parnaiba Delta, Maranhao state, northeastern Brazil. Braz J Biol 68: 545-551. **marine mammal, Atlantic SW**

375. Manly BFJ, Moulton VD, Elliott RE, Miller GW, Richardson WJ (2007) Analysis of covariance of fall migrations of bowhead whales in relation to human activities and environmental factors, Alaskan Beaufort Sea: Phase I, 1996-1998. OCS Study Report MMS 2005-033. **marine mammal, Arctic**

376. Marcoux M, Whitehead H, Rendell L (2007) Sperm whale feeding variation by location, year, social group and clan: Evidence from stable isotopes. Mar Ecol Prog Ser 333: 309-314. **marine mammal, Pacific CE, Pacific SE**

377. Marques TA, Thomas L, Ward J, DiMarzio N, Tyack PL (2009) Estimating cetacean population density using fixed passive acoustic sensors: An example with Blainville’s beaked whales. J Acoust Soc Am 125: 1982–1994. **marine mammal, Atlantic CW**

378. Mate BR, Gisiner R, Mobley J (1998) Local and migratory movements of Hawaiian humpback whales tracked by satellite telemetry. Can J Zool 76: 863-868. **marine mammal, Pacific CE**

379. Mate BR, Krutzikowsky GK, Winsor MH (2000) Satellite-monitored movements of radio-tagged bowhead whales in the Beaufort and Chukchi seas during the late-summer feeding season and fall migration. Can J Zool 78: 1168-1181. **marine mammal, Arctic**

380. Mate BR, Lagerquist BA, Calambokidis J (1999) Movements of north Pacific blue whales during the feeding season off southern California and their southern fall migration. Mar Mamm Sci 15: 1246-1257. **marine mammal, Pacific NE**

381. Mate BR, Lagerquist BA, Winsor M, Geraci J, Prescott JH (2005) Movements and dive habits of a satellite-monitored longfinned pilot whale (*Globicephala melas*) in the northwest Atlantic. Mar Mamm Sci 21: 136-144. **marine mammal, Pacific NE**

382. Mate BR, Nieukirk SL, Kraus SD (1997) Satellite-monitored movements of the northern right whale. J Wildl Manag 61: 1393-1405. **marine mammal, Atlantic NW**

383. Mate BR, Stafford KM, Ljungblad DK (1994) A change in sperm whale (*Physeter macrocephalus*) distribution correlated to seismic surveys in the Gulf of Mexico. J Acoust Soc Am 96: 3268-3269. **marine mammal, Atlantic CW**

384. Mate BR, Urban-Ramirez J (2003) A note on the route and speed of a gray whale on its northern migration from Mexico to central California, tracked by satellite-monitored radio tag. J Cetacean Res Manag 5: 155-157. **marine mammal, Pacific CE**

385. McClellan DB (1996) Aerial surveys for sea turtles, marine mammals, and vessel activity along the southeast Florida coast: 1992-1996. Miami, FL: NOAA Technical Memorandum NMFS-SEFSC-390. **marine mammal, sea turtle, Atlantic CW**

386. Medina-Vogel G, Bartheld JL, Pacheco RA, Rodriguez CD (2006) Population assessment and habitat use by marine otter *Lontra felina* in southern Chile. Wildl Biol 12: 191-199. **marine mammal, Pacific SE**

387. Medina-Vogel G, Boher F, Flores G, Santibanez A, Soto-Azat C (2007) Spacing behavior of marine otters (*Lontra felina*) in relation to land refuges and fishery waste in central Chile. J Mammal 88: 487-494. **marine mammal, Pacific SE**

388. Medina-Vogel G, Delgado C, Alvarez R, Bartheld JL (2004) Feeding ecology of the marine otter (*Lontra felina*) in a rocky seashore of the south of Chile. Mar Mamm Sci 20: 134–144. **marine mammal, Pacific SE**

389. Medina-Vogel G, Merino LO, Alarcón RM, Vianna JdA (2008) Coastal-marine discontinuities, critical patch size and isolation: implications for marine otter conservation. Anim Conserv 11: 57-64. **marine mammal, Pacific CE**

390. Meier SK, Yazvenko SB, Blokhin SA, Wainwright P, Maminov MK, et al. (2007) Distribution and abundance of western gray whales off northeastern Sakhalin Island, Russia, 2001–2003. Environ Monit Assess 134: 107-136. **marine mammal, Pacific NW**

391. Meirelles ACO, Monteiro-Neto C, Martins AMA, Costa AF, Barros HMDR, et al. (2009) Cetacean strandings on the coast of Ceara, north-eastern Brazil (1992-2005). J Mar Biol Assoc U K 89: 1083-1090. **marine mammal, Atlantic SW**

392. Mignucci-Giannoni AA, Swartz SL, Martinez A, Burks CM, Watkins WA (2003) First records of the pantropical spotted dolphin (*Stenella attenuata*) for the Puerto Rican Bank, with a review of the species in the Caribbean. Caribb J Sci 39: 381-392. **marine mammal, Atlantic CW**

393. Miller JD, Dobbs KA, Limpus CJ, Mattocks N, Landry J, A.M. (1998) Long-distance migrations by the hawksbill turtle, *Eretmochelys imbricata*, from north-eastern Australia. Wildl Res 25: 89-95. **sea turtle, Pacific CW**

394. Miyashita T (1993) Abundance of dolphin stocks in the western north Pacific taken by the Japanese drive fishery. Rep Int Whaling Comm 43: 417-437. **marine mammal, Pacific NW**

395. Miyashita T (2001) Cruise report of the Japanese sighting survey under the joint research program, between Japan and Korea in 2000 and 2001. Paper SC/53/RMP6 presented to the International Whaling Commission Scientific Committee. **marine mammal, Pacific NW**

396. Miyashita T (2003) Report of the Japanese sighting survey under the Japan/Korea joint project in 2003. Paper SC/56/RMP2 presented to the International Whaling Commission. Shizuoka, Japan: National Research Institute of Far Seas Fisheries. **marine mammal, Pacific NW**

397. Miyashita T, Fujise Y (1997) Abundance estimate of the western north Pacific minke whale in Sub-Area 9 with notes on the results of dedicated sighting surveys. Rep Int Whaling Comm 47: 543-551. **marine mammal, Pacific NW**

398. Miyashita T, Yoshida H (2003) Report of the Japanese sighting survey the Japan/Korea joint project in 2002. Paper SC/55/RMP5 presented to the International Whaling Commission Scientific Committee. **marine mammal, Pacific NW**

399. Miyazaki N, Kato H (1988) Sightings records of small cetaceans in the southern hemisphere. Bull Natl Sci Mus, Tokyo Ser A 14: 47-65. **marine mammal, Pacific Antarctic**

400. Mizroch SA, Rice DW, Zwiefelhofer D, Waite J, Perryman WL (2009) Distribution and movements of fin whales in the north Pacific Ocean. Mammal Rev 39: 193-227. **marine mammal, Pacific NE, Pacific NW**

401. Mobley J, J.R., Spitz SS, Forney KA, Grotefendt R, Forestell PH (2000) Distribution and abundance of Odontocete species in Hawaiian waters: Preliminary results of 1993-98 aerial surveys. NOAA-NMFS-SWFSC Administrative Report LJ-00-14C. **marine mammal, Pacific CE**

402. Mobley JR, Jr. (2001) Results of 2001 aerial surveys of humpback whales, north of Kauai: Annual report submitted to the North Pacific Acoustic Laboratory (NPAL) Program, Scripps Institution of Oceanography. Marine Mammal Research Consultants, Ltd. **marine mammal, Pacific CE**

403. Mobley JR, Jr. (2002) Results of 2002 aerial surveys of humpback whales, north of Kauai: Annual report submitted to the North Pacific Acoustic Laboratory (NPAL) Program, Scripps Institution of Oceanography. Marine Mammal Research Consultants, Ltd. **marine mammal, Pacific CE**

404. Mobley JR, Jr. (2003) Results of 2003 aerial surveys of humpback whales north of Kauai: Annual report submitted to the North Pacific Acoustic Laboratory (NPAL) Program, Scripps Institution of Oceanography. Marine Mammal Research Consultants, Ltd. **marine mammal, Pacific CE**

405. Mobley JR, Jr. (2004) Results of marine mammal surveys on U.S. Navy underwater ranges in Hawaii and Bahamas. Final report submitted to Office of Naval Research (ONR) Marine Mammal Program. Marine Mammal Research Consultants, Ltd. **marine mammal, Atlantic CW, Pacific CE**

406. Mobley JR, Jr. (2004) Results of 2004 aerial surveys of humpback whales north of Kauai: Annual report submitted to the North Pacific Acoustic Laboratory (NPAL) Program, Scripps Institution of Oceanography. Marine Mammal Research Consultants, Ltd. **marine mammal, Pacific CE**

407. Mobley JR (2005) Results of 2005 aerial surveys of humpback whales north of Kauai: Quicklook report submitted to the North Pacific Acoustic Laboratory (NPAL) Program, Scripps Institution of Oceanography. Marine Mammal Research Consultants, Ltd. **marine mammal, Pacific CE**

408. Mobley JR, Jr. (2006) Results of 2006 RIMPAC aerial surveys of marine mammals in Kaulakahi and Alenuihaha Channels. Final report submitted to: Environmental Division Commander, U.S. Pacific Fleet. Marine Mammal Research Consultants, Ltd. **marine mammal, Pacific CE**

409. Mobley JR, Jr., Forestell PH, Grotefendt R (1997) Preliminary results of 1993 and 1995 aerial surveys in Hawaiian waters. In: Payne PM, Phillips B, Nitta E, editors. Report of the workshop to assess research and other needs and opportunities related to humpback whale management in the Hawaiian Islands, April 26-28. Kaanapali, Maui, Hawaii. **marine mammal, Pacific CE**

410. Mobley JR, Jr., Forestell PH, Grotefendt RA (1994) Results of 1993 aerial surveys in Hawaiian waters. In: Bowles A, editor. 1993 ATOC Marine Mammal Research Program annual report, submitted to Advanced Research Projects Agency (ARPA). **marine mammal, Pacific CE**

411. Mobley JR, Jr., Grotefendt RA, Forestell PH, Frankel AS (1999) Results of aerial surveys of marine mammals in the major Hawaiian Islands (1993-98): Final report to the Acoustic Thermometry of Ocean Climate Program (ATOC MMRP). Pearl City, Hawaii: University of Hawaii – West Oahu. **marine mammal, Pacific CE**

412. Mobley JR, Jr., Mazzuca L, Craig AS, Newcomer MW, Spitz SS (2001) Killer whales (*Orcinus orca*) sighted west of Ni’ihau, Hawai’i. Pac Sci 55: 301-303. **marine mammal, Pacific CE**

413. Mobley JR, Jr., Smultea M, Norris T, Weller D (1996) Fin whale sighting north of Kaua'i, Hawai'i. Pac Sci 50: 230-233. **marine mammal, Pacific CE**

414. Mobley JR, Jr., Spitz S, Grotefendt R, Forestell P, Frankel A, et al. (2001) Abundance of humpback whales in Hawaiian waters: Results of 1993-2000 aerial surveys. Report prepared for the Hawaiian Islands Humpback Whale National Marine Sanctuary, Office of National Marine Sanctuaries, National Oceanic and Atmospheric Administration, U.S. Department of Commerce and the Department of Land and Natural Resources State of Hawaii. Marine Mammal Research Consultants. **marine mammal, Pacific CE**

415. Moissinen E (1997) Contingent valuation: The case of the Saimaa seal. Joensuu, Finland. Joensuu, Finland: University of Joensuu. **marine mammal, Atlantic NE**

416. Moore SE (1992) Summer records of bowhead whales in the northeastern Chukchi Sea. Arctic 45: 398-400. **marine mammal, Pacific NE**

417. Moore SE, Clarke JT (1992) Patterns of bowhead whale distribution and abundance near Barrow, Alaska, in fall 1982-1989. Mar Mamm Sci 8: 27-36. **marine mammal, Arctic**

418. Moore SE, Clarke JT, Johnson MM (1993) Beluga distribution and movements offshore northern Alaska in spring and summer, 1980-84. Rep Int Whaling Comm 0: 375-386. **marine mammal, Arctic, Pacific NE**

419. Moore SE, DeMaster DP, Dayton PK (2000) Cetacean habitat selection in the Alaskan Arctic during summer and autumn. Arctic 53: 432-447. **marine mammal, Arctic**

420. Moore SE, Laidre KL (2006) Trends in sea ice cover within habitats used by bowhead whales in the western Arctic. Ecol Appl 16: 932-944. **marine mammal, Arctic**

421. Nishiwaki M, Yamaguchi M, Shokita S, Uchida S, Kataoka T (1982) Recent survey on the distribution of the African manatee *Trichechus semegalensis*. Sci Rep Whales Res Inst, Tokyo: 137-148. **marine mammal, Atlantic CE**

422. Norman SA, Bowlby CE, Brancato MS, Calambokidis J, Duffield D, et al. (2004) Cetacean strandings in Oregon and Washington between 1930 and 2002. J Cetacean Res Manag 6: 87-99. **marine mammal, Pacific NE**

423. Notarbartolo di Sciara G, Agardy T, Hyrenbach D, Scovazzi T, Van Klaveren P (2008) The Pelagos Sanctuary for Mediterranean marine mammals. Aquat Conserv: Mar Freshwat Ecosyst 18: 367-391. **marine mammal, Mediterranean**

424. Notarbartolo di Sciara G, Zanardelli M, Jahoda M, Panigada S, Airoldi S (2003) The fin whale *Balaenoptera physalus* (L. 1758) in the Mediterranean Sea. Mammal Rev 33: 105-150. **marine mammal, Mediterranean**

425. Nottestad L, Doksaeter L, Langard L, Langoy H, Tennington E, et al. (2007) Distribution and feeding ecology of fin- and humpback whales during summer in the Norwegian Sea. 17th biennial conference on the Biology of Marine Mammals (SMM), 29 November - 3 December 2007. Capetown, South Africa. **marine mammal, Atlantic NE**

426. Nuti S, Giorli G, Bedocchi D (2006) Range analysis of *Tursiops truncatus* along the north-Tuscany coasts by means of GIS system. Biol Mar Medit 13: 280-281. **marine mammal, Mediterranean**

427. Ohsumi S, Kasamatsu F (1986) Recent off-shore distribution of the southern right whale in summer. Appendix. Natural marking of right whale. Rep Int Whaling Comm Special Issue 10: 185. **marine mammal, Pacific SE, Atlantic SW, Atlantic SE, Indian W, Indian E, Pacific CW, Pacific SW, Pacific CE, Pacific Antarctic, Atlantic Antarctic, Indian Antarctic**

428. Øien N (1996) Cruise and data report from the Norwegian sighting survey 1995 (NILS-95). Abundance Estimation Working Group: International Whaling Commission Scientific Paper SC/48/NA3. **marine mammal, Atlantic NE**

429. Oien N, Fagerheim K, Hartvedt S (2007) Distribution patterns of humpback whales (*Megaptera novaeangliae*) in the Barents and Norwegian Seas. 17th biennial conference on the Biology of Marine Mammals (SMM), 29 November - 3 December 2007. Capetown, South Africa. **marine mammal, Atlantic NE**

430. Olson PA (2008) Status of blue whale photo-identification from IWC IDCR/SOWER cruises 1987-1988 to 2007-2008. Paper SC/60/SH29 presented to the International Whaling Commission Scientific Committee. **marine mammal, Pacific Antarctic**

431. Ortega-Ortiz J, Mate B (2007) Foraging areas and migration timing of humpback whales from the western Antarctic Peninsula. 17th biennial conference on the Biology of Marine Mammals (SMM), 29 November - 3 December 2007. Capetown, South Africa. **marine mammal, Atlantic Antarctic**

432. Palazzo JT, Groch K (2007) A record year for southern right whale records off Brazil. 17th biennial conference on the Biology of Marine Mammals (SMM), 29 November - 3 December 2007. Capetown, South Africa. **marine mammal, Atlantic SW**

433. Panigada S, Di Sciara GN, Panigada MZ, Airoldi S, Borsani JF, et al. (2005) Fin whales (*Balaenoptera physalus*) summering in the Ligurian Sea: Distribution, encounter rate, mean group size and relation to physiographic variables. J Cetacean Res Manag 7: 137-125. **marine mammal, Mediterranean**

434. Papastavrou V, Salm RV (1991) A note on recent sightings and strandings of cetaceans in Oman: Ra’s Sawadi to Rakhyut. In: Leatherwood S, Donovan GP, editors. Cetaceans and cetacean research in the Indian Ocean Sanctuary, marine mammal technical report number 3. Nairobi, Kenya: United Nations Environment Program (UNEP). pp. 211-218. **marine mammal, Indian W, Indian E**

435. Parra G, J. (2006) Resource partitioning in sympatric delphinids: Space use and habitat preferences of Australian snubfin and Indo-Pacific humpback dolphins. J Anim Ecol 75: 862-874. **marine mammal, Pacific CW**

436. Parra GJ, Azuma C, Preen AR, Corkeron PJ, Marsh H (2002) Distribution of Irrawaddy dolphins, *Orcaella brevirostris*, in Australian waters. Raffles Bull Zool: 141-154. **marine mammal, Pacific SW, Pacific CW, Indian E**

437. Parra GJ, Corkeron PJ, Marsh H (2004) The Indo-Pacific humpback dolphin, *Sousa chinensis* (Osbeck, 1765), in Australian waters: A summary of current knowledge. Aquat Mamm 30: 197-206. **marine mammal, Indian E**

438. Parra GJ, Corkeron PJ, Marsh H (2006) Population sizes, site fidelity and residence patterns of Australian snubfin and Indo-Pacific humpback dolphins: Implications for conservation. Biol Conserv 129: 167-180. **marine mammal, Pacific CW**

439. Parra GJ, Schick RS, Corkeron PJ (2006) Spatial distribution and environmental correlates of Australian snubfin and Indo-Pacific humpback dolphins. Ecography 29: 396-406. **marine mammal, Pacific CW**

440. Parrish FA, Marshall GY, Buhleier B, Antonelis GA (2008) Foraging interaction between monk seals and large predatory fish in the Northwestern Hawaiian Islands. Endang Species Res 4: 299-308. **marine mammal, Pacific CE**

441. Parsons ECM (1998) Observations of Indo-Pacific humpbacked dolphins, *Sousa Chinensis*, from Goa, western India. Mar Mamm Sci 14: 166-170. **marine mammal, Indian W**

442. Parsons ECM, Calventi IBD, Whaley A, Rose NA, Sherwin S (2007) A note on illegal captures of wild bottlenose dolphins (*Tursiops truncatus*) from the coastal waters of the Dominican Republic. Paper SC/58/SM11 presented to the International Whaling Commission Scientific Committee. **marine mammal, Atlantic CW**

443. Paton D, Gibbs N, Childerhouse S, Clapham P (2007) Assessment of the current abundance of humpback whales in the Lomaivit Island Group of Fiji, and a comparison with historical data. 17th biennial conference on the Biology of Marine Mammals (SMM), 29 November - 3 December 2007. Capetown, South Africa. **marine mammal, Pacific SW**

444. Penrose RS (2002) UK and Eire marine turtle strandings and sightings annual report 2001. Cardigan, Wales: Marine Environmental Monitoring. **sea turtle, Atlantic NE**

445. Penrose RS (2003) UK and Eire marine turtle strandings and sightings annual report 2002. Cardigan, Wales: Marine Environmental Monitoring. **sea turtle, Atlantic NE**

446. Penrose RS (2004) UK and Eire marine turtle strandings and sightings annual report 2003. Cardigan, Wales: Marine Environmental Monitoring. **sea turtle, Atlantic NE**

447. Penrose RS (2005) UK and Eire marine turtle strandings and sightings annual report 2004. Cardigan, Wales: Marine Environmental Monitoring. **sea turtle, Atlantic NE**

448. Penrose RS, Gander LR (2006) UK and Eire marine turtle strandings and sightings annual report 2005. Cardigan, Wales: Marine Environmental Monitoring. **sea turtle, Atlantic NE**

449. Perlov AS, Vladimirov V, Reviakina ZV (1996) Review of literature/information regarding marine mammals in the vicinity of Sakhalin Island, Okhotsk Sea, Russia. In: Ismail-Zade J, Yazvenko S, Johnson SR, editors. Houston, TX: Marathon Upstream Sakhalin Services, Ltd. **marine mammal, Pacific NW**

450. Piatt JF, Drew G (2003) North Pacific pelagic seabird database (NPPSD): Compiling datasets and creating an archive, accessible database, and pelagic seabird atlas. Semi-annual progress report (July 1 - December 31, 2002) of Project NPMRI 18 (T2110) to the North Pacific Marine Research Institute. Anchorage, AK: U.S. Geological Survey, Alaska Biological Science Center. **seabird, Pacific NE, Pacific NW**

451. Picanco C, Carvalho I, Brito C (2009) Occurrence and distribution of cetaceans in São Tomé and Príncipe tropical archipelago and their relation to environmental variables. J Mar Biol Assoc U K 89: 1071-1076. **marine mammal, Atlantic CE, Atlantic SE**

452. Pierpoint C, Allan L, Arnold H, Evans P, Perry S, et al. (2009) Monitoring important coastal sites for bottlenose dolphin in Cardigan Bay, UK. J Mar Biol Assoc U K 89: 1033-1043. **marine mammal, Atlantic NE**

453. Pinedo MC, Polacheck T, Barreto AS (2002) Preliminary results of the cetacean sightings surveys off the southern coast of Brazil: Spring 1996 and winter 1997. Paper presented to the International Whaling Commission Scientific Committee. **marine mammal, Atlantic SW**

454. Pinedo MC, Polacheck T, Barreto AS, Lammardo MP (2002) A note on vessel of opportunity sighting surveys for cetaceans in the shelf edge region off the southern coast of Brazil. J Cetacean Res Manag 4: 323-329. **marine mammal, Atlantic SW**

455. Pitcher KW, Olesiuk PF, Brown RF, Lowry MS, Jeffries SJ, et al. (2007) Abundance and distribution of the eastern north Pacific Steller sea lion (*Eumetopias jubatus*) population. Fish Bull 107: 102-115. **marine mammal, Pacific NE, Pacific CE**

456. Pitman RL (1990) Pelagic distribution and biology of sea turtles in the eastern tropical Pacific. In: Richardson TH, Richardson JI, Donnelly M, editors. 10th annual workshop on sea turtle biology and conservation. Miami, FL: NOAA Technical Memorandum NMFS-SEFSC-278. pp. 143-148. **sea turtle, Pacific SE**

457. Pollock CM, Mavor R, Weir CR, Reid A, White RW, et al. (2000) The distribution of seabirds and marine mammals in the Atlantic frontier, north and west of Scotland. Aberdeen, Scotland: Joint Nature Conservation Committee, Seabirds and Cetaceans. **marine mammal, seabird, Atlantic NE**

458. Potter JR, Thillet M, Douglas C, Chitre MA (2007) Visual and passive acoustic marine mammal observations and high-frequency seismic source characteristics recorded during a seismic survey. IEEE J Oceanic Eng 32: 469-483. **marine mammal, Atlantic NW**

459. Powell JA (1987) Nigeria - status of west African manatees. Sirenews: 7-9. **marine mammal, Atlantic CE**

460. Praca E, Gannier A, Das K, Laran S (2009) Modelling the habitat suitability of cetaceans: Example of the sperm whale in the northwestern Mediterranean Sea Deep-Sea Res Part I: Oceanogr Res Pap 56: 10. **marine mammal, Mediterranean**

461. Rademeyer R, Best PB, Butterworth DS (2001) Blue whales on the Madagascar Plateau, December 1996. Paper SC/53/IA24 presented to the International Whaling Commission Scientific Committee. **marine mammal, Indian W**

462. Raum-Suryan K, Pitcher K, Lamy R (2005) Sea otter (*Enhydra lutris*) sightings off Haida Gwaii/Queen Charlotte Islands, Canada during the past three decades. Can Field-Nat 118: 270-272. **marine mammal, Pacific NE**

463. Raum-Suryan KL, Harvey JT (1998) Distribution and abundance of and habitat use by harbor porpoise, *Phocoena phocoena*, off the northern San Juan Islands, Washington. Fish Bull 96: 808-822. **marine mammal, Pacific NE**

464. Raum-Suryan KL, Pitcher KW, Calkins DG, Sease JL, Loughlin TR (2002) Dispersal, rookery fidelity, and metapopulation structure of Steller sea lions (*Eumetopias jubatus*) in an increasing and a decreasing population in Alaska. Mar Mamm Sci 18: 746-764. **marine mammal, Pacific CE**

465. Raum-Suryan KL, Rehberg MJ, Pendleton GW, Pitcher KW, Gelatt TS (2004) Development of dispersal, movement patterns, and haul-out use by pup and juvenile Steller sea lions (*Eumetopias jubatus*) in Alaska. Mar Mamm Sci 20: 823-850. **marine mammal, Pacific NE**

466. Reeves R, Smith TD, Josephson EA, Clapham PJ, Woolmer G (2004) Historical observations of humpback and blue whales in the North Atlantic Ocean: Clues to migratory routes and possibly additional feeding grounds. Mar Mamm Sci 20: 774-786. **marine mammal, Atlantic NW, Atlantic NE, Atlantic CW, Atlantic CE**

467. Reeves RR, Leatherwood S, Baird RW (2009) Evidence of a possible decline since 1989 in false killer whales (*Pseudorca crassidens*) around the main Hawaiian Islands. Pac Sci 63: 253-261. **marine mammal, Pacific CE**

468. Rendell L, Whitehead H, Escribano R (2004) Sperm whale habitat use and foraging success off northern Chile: Evidence of ecological links between coastal and pelagic systems. Mar Ecol Prog Ser 275: 289-295. **marine mammal, Pacific SE**

469. Reyes LM (2007) Marine conservation programme for central Patagonia, Argentina: Setting basis for the creation of marine protected areas; Final report for Rufford Small Grants Foundation. Puerto Madryn - Chubut, Argentina. **marine mammal, Atlantic SW**

470. Reyes LM, Crespo EA, Szapkievich V (1999) Distribution and population size of the southern sea lion (*Otaria flavescens*) in central and southern Chubut, Patagonia, Argentina. Mar Mamm Sci 15: 478-493. **marine mammal, Atlantic SW**

471. Rice DW (1977) Sperm whales in the equatorial eastern Pacific: Population size and social organisation. Rep Int Whaling Comm 27: 333-336. **marine mammal, Pacific SE, Pacific CE**

472. Rice DW, Wolman AA (1982) Whale census in the Gulf of Alaska June to August 1980. Rep Int Whaling Comm 32: 491-497. **marine mammal, Pacific NE**

473. Rice MR, Balazs GH (2008) Diving behavior of the Hawaiian green turtle (*Chelonia mydas*) during oceanic migrations. J Exp Mar Biol Ecol 356: 121-127. **sea turtles, Pacific CE**

474. Richard PR, Heide-Jorgensen MP, St Aubin D (1998) Fall movements of belugas (*Delphinapterus leucas*) with satellite-linked transmitters in Lancaster Sound, Jones Sound, and northern Baffin Bay. Arctic 51: 5-16. **marine mammal, Atlantic NW**

475. Richard PR, Martin AR, Orr JR (2001) Summer and autumn movements of belugas of the eastern Beaufort Sea stock. Arctic 54: 223-236. **marine mammal, Arctic**

476. Richard PR, Orr JR, Dietz R, Dueck L (1998) Sightings of belugas and other marine mammals in the North Water, late March 1993. Arctic 51: 1-4. **marine mammal, Atlantic NW**

477. Robineau D (1991) Balaenopterid sightings in the western tropical Indian Ocean (Seychelles area), 1982-1986. In: Leatherwood S, Donovan GP, editors. Cetaceans and cetacean research in the Indian Ocean Sanctuary, marine mammal technical report number 3. Nairobi, Kenya: United Nations Environment Program (UNEP). pp. 171-178. **marine mammal, Indian W**

478. Robinson K, Tetley M, Mitchelson-Jacob E (2009) The distribution and habitat preference of coastally occurring minke whales (*Balaenoptera acutorostrata*) in the outer southern Moray Firth, northeast Scotland. J Coast Conserv 13: 39-48. **marine mammal, Atlantic NE**

479. Rojas-Bracho L, Gerrodette T, Ballance LT, Jaramillo-Legorreta A, Urban-Ramirez J, et al. (2007) Distribution of cetaceans in Mexican Pacific EEZ waters. 17th biennial conference on the Biology of Marine Mammals (SMM), 29 November - 3 December 2007. Capetown, South Africa. **marine mammal, Pacific CE**

480. Romero A, Ignacio Agudo A, Green SM, Notarbartolo di Sciara G (2001) Cetaceans of Venezuela: Their distribution and conservation status. Seattle, Washington: NOAA Technical Report NMFS-151. **marine mammal, Atlantic CW**

481. Rossi-Santos M, Baracho C, Cipolotti S, Lima F, Marcovaldi E, et al. (2007) Occurrence and distribution of humpback whales in the north coast of Bahia State, Brazil, between 2000 and 2006. 17th biennial conference on the Biology of Marine Mammals (SMM), 29 November - 3 December 2007. Capetown, South Africa. **marine mammal, Atlantic SW**

482. Ross-Santos M, Baracho C, Neto ES, Marcovaldi E (2006) First sightings of the pygmy killer whale, *Feresa attenuata*, for the Brazilian coast. JMBA2 Biodiversity Records. **marine mammal, Atlantic SW**

483. Rugh D (2008) Bowhead whale abundance through photographic analysis: Data analysis supported by Minerals Management Service. Seattle, WA: NOAA-NMFS-AFSC-NMML OCS Study Report MMS 2008-002. **marine mammal, Atlantic CW, Pacific CE, Pacific NE**

484. Rugh DJ, Goetz KT, Mahoney BA (2005) Aerial survey of belugas in Cook Inlet, Alaska, August 2005. Unpublished NMFS report. Seattle, WA: NOAA-NMFS-AFSC. **marine mammal, Pacific NE**

485. Rugh DJ, Goetz KT, Mocklin JA, Mahoney BA, Smith BK (2007) Aerial surveys of belugas in Cook Inlet, Alaska, June 2007. Unpublished NMFS report. Seattle, WA: NOAA-NMFS-AFSC. **marine mammal, Pacific NE**

486. Rugh DJ, Goetz KT, Sims CL (2006) Aerial surveys of belugas in Cook Inlet, Alaska, May 2006. Unpublished NMFS report. Seattle, WA: NOAA-NMFS-AFSC. **marine mammal, Pacific NE**

487. Rugh DJ, Goetz KT, Sims CL, Smith BK (2006) Aerial surveys of belugas in Cook Inlet, Alaska, August 2006. Unpublished NMFS report. Seattle, WA: NOAA-NMFS-AFSC. **marine mammal, Pacific NE**

488. Rugh DJ, Mahoney BA, Smith BK (2004) Aerial surveys of beluga whales in Cook Inlet, Alaska, between June 2001 and June 2002. Seattle, WA: NOAA Technical Memorandum NMFS-AFSC-145. **marine mammal, Pacific NE**

489. Rugh DJ, Shelden KEW, Mahoney BA (2000) Distribution of belugas, *Delphinapterus leucas*, in Cook Inlet, Alaska, during June/July 1993-2000. Mar Fish Rev 62: 6-21. **marine mammal, Pacific NE**

490. Rugh DJ, Shelden KEW, Mahoney BA, Litzky LK (2000) Aerial surveys of beluga in Cook Inlet, Alaska, June 2000. Unpublished NMFS report. Seattle, WA: NOAA-NMFS-AFSC. **marine mammal, Pacific NE**

491. Rugh DJ, Shelden KEW, Mahoney BA, Litzky LK, Hobbs RC, et al. (1999) Aerial surveys of beluga whales in Cook Inlet, Alaska, June 1999. Unpublished NMFS report. Seattle, WA: NOAA-NMFS-AFSC. **marine mammal, Pacific NE**

492. Rugh DJ, Shelden KEW, Sims CL, Mahoney BA, Smith BK, et al. (2004) Aerial surveys of belugas in Cook Inlet, Alaska, June 2004. Unpublished NMFS report. Seattle, WA: NOAA-NMFS-AFSC. **marine mammal, Pacific NE**

493. Rugh DJ, Shelden KEW, Sims CL, Mahoney BA, Smith BK, et al. (2005) Aerial surveys of belugas in Cook Inlet, Alaska, June 2001, 2002, 2003, and 2004. Seattle, WA: NOAA Technical Memorandum NMFS-AFSC-149. **marine mammal, Pacific NE**

494. Sabin RC (2001) The Natural History Museum UK Cetacean Strandings recording scheme. Porcupine Marine Natural History Society Newsletter. Newtonards, United Kingdom: Porcupine Marine Natural History Society. **marine mammal, Atlantic NE**

495. Sabin RC, Jepson PD, Reid A, Chimonides PJ, Deaville R, et al. (2002) Trends in cetacean strandings around the UK coastline and marine mammal postmortem investigations for the year 2001 (CRO 238). Report No. ECM 516F00/02 for the Department for Environment, Food and Rural Affairs. London, United Kingdom: Natural History Museum Consultancy. **marine mammal, Atlantic NE**

496. Sabin RC, Jepson PD, Reid A, Deaville R, Muir AI, et al. (2001) Trends in cetacean strandings around the UK coastline and marine mammal postmortem investigations for the year 2000 (CRO 238). Report No. ECM 516/00 for the Department for Environment, Food and Rural Affairs. London, United Kingdom: Natural History Museum Consultancy. **marine mammal, Atlantic NE**

497. Sakhalin Energy Investment Company LTD. (2006) Marine mammal observation program close out report. Rev. 01. Yuzhno-Sakhalinsk, Russia: Sakhalin Energy Investment Company Ltd. **marine mammal, Pacific NW**

498. Sakhalin Energy Investment Company LTD. (2006) Distribution and abundance of gray whales of the Okhotsk-Korean population off northeastern Sakhalin, June-November 2005 (based on data from onshore, aerial and vessel-based surveys). Yuzhno-Sakhalinsk, Russia: Sakhalin Energy Investment Company Ltd. **marine mammal, Pacific NW**

499. Salm RV, Jensen RAC, Papastavrou VA (1993) Marine fauna of Oman: Cetaceans, turtles, seabirds, and shallow water corals: A marine conservation and development report. Gland, Switzerland: World Conservation Union. **marine mammal, sea turtle, seabird, Indian W**

500. Santora J, Loeb V, Reiss C (2007) Distribution of humpback whales in the western Antarctic Peninsula region: Relationships with krill demography and oceanography. 17th biennial conference on the Biology of Marine Mammals (SMM), 29 November - 3 December 2007. Capetown, South Africa. **marine mammal, Atlantic Antarctic**

501. Scarff JE (1986) Historic and present distribution of the right whale (*Eubalaena glacialis*) in the eastern north Pacific south of 50 N and east of 180 W. Rep Int Whaling Comm Special Issue 20: 43-63. **marine mammal, Pacific NE**

502. Schick RS, Urban DL (2000) Spatial components of bowhead whale (*Balaena mysticetus*) distribution in the Alaskan Beaufort Sea. Can J Fish Aquat Sci 57: 2193-2200. **marine mammal, Arctic**

503. Schofield G, Bishop CM, Katselidis KA, Dimopoulos P, Pantis JD, et al. (2009) Microhabitat selection by sea turtles in a dynamic thermal marine environment. J Anim Ecol 78: 14-21. **sea turtle, Mediterranean**

504. Sease JL, Lewis JP, McAllister DC, Merrick RL, Mello SM (1993) Aerial and ship-based surveys of Steller sea lions (*Eumetopias jubatus*) in southeast Alaska, the Gulf of Alaska, and Aleutian Islands during June and July 1992. Seattle, WA: NOAA Technical Memorandum NMFS-AFSC-17. **marine mammal, Pacific NE**

505. Sease JL, York A (2003) Seasonal distribution of Steller's sea lions at rookeries and haul-out sites in Alaska. Mar Mamm Sci 19: 745-763. **marine mammal, Pacific NE, Pacific NW**

506. Sekiguchi K, Howell E (2007) The transition zone chlorophyll front: A factor of cetacean species distribution in the central north Pacific? 17th biennial conference on the Biology of Marine Mammals (SMM), 29 November - 3 December 2007. Capetown, South Africa. **marine mammal, Pacific NE, Pacific CE**

507. Sekiguchi K, Olavarria C, Morse L, Olson P, Ensor P, et al. (2006) The spectacled porpoise (*Phocoena diptrica*) in Antarctic waters. J Cetacean Res Manag 8: 265-271. **marine mammal, Pacific Antarctic, Indian Antarctic, Atlantic Antarctic**

508. Servidio A, Alves F, Dinis A, Freitas L, Martin V (2007) First record of movement of short-finned pilot whales between two Atlantic oceanic archipelagos. 17th biennial conference on the Biology of Marine Mammals (SMM), 29 November - 3 December 2007. Capetown, South Africa. **marine mammal, Atlantic CE**

509. Shaffer SA, Tremblay Y, Awkerman JA, Henry RW, Teo SLH, et al. (2005) Comparison of light- and SST-based geolocation with satellite telemetry in free-ranging albatrosses. Mar Biol 147: 833-843. **seabird, Pacific CE**

510. Shanker K (2004) Marine turtle status and conservation in the Indian Ocean. In: FAO, editor. Papers presented at the expert consultation on interactions between sea turtles and fisheries within an ecosystem context. Rome, Italy: FAO. pp. 85-134. **sea turtle, Indian W, Indian E**

511. Shelden KEW, Goetz KT, Brattstrom LV, Mahoney BA, Migura-Krajzynski M, et al. (2008) Aerial surveys of belugas in Cook Inlet, Alaska, August 2008. Seattle, WA: NOAA-NMFS-AFSC. **marine mammal, Pacific NE**

512. Shelden KEW, Goetz KT, Mocklin JA (2007) Aerial surveys of belugas in Cook Inlet, Alaska, August 2007. Seattle, WA: NOAA-NMFS-AFSC. **marine mammal, Pacific NE**

513. Shelden KEW, Moore SE, Waite JM, Wade PR, Rugh DJ (2005) Historic and current habitat use by north Pacific right whales *Eubalaena japonica* in the Bering Sea and Gulf of Alaska. Mammal Rev 35: 129-155. **marine mammal, Pacific NE**

514. Shelden KEW, Rugh DJ, Goetz KT, Brattstrom LV, Mahoney BA (2008) Aerial surveys of belugas in Cook Inlet, Alaska, June 2008. Unpublished NMFS report. Seattle, WA: NOAA-NMFS-AFSC. **marine mammal, Pacific NE**

515. Sheppard JK, Preen AR, Marsh H, Lawler IR, Whiting SD, et al. (2006) Movement heterogeneity of dugongs, *Dugon dugong* (Muller), over large spatial scales. J Exp Mar Biol Ecol 334: 20. **marine mammal, Pacific CW**

516. Shillinger GL, Palacios DM, Bailey H, Bograd SJ, Swithenbank AM, et al. (2008) Persistent leatherback turtle migrations present opportunities for conservation. PLoS Biol 6: 1408-1416. **sea turtle, Pacific CE, Pacific SE**

517. Shimada H, Pastene LA (1995) Report of a sighting survey off the Solomon Islands with comments on Bryde’s whale distribution. Rep Int Whaling Comm 45: 413-428. **marine mammal, Pacific CW**

518. Shucksmith R, Jones NH, Stoyle GW, Davies A, Dicks EF (2009) Abundance and distribution of the harbour porpoise (*Phocoena phocoena*) on the north coast of Anglesey, Wales, UK. J Mar Biol Assoc U K 89: 1051-1058. **marine mammal, Atlantic NE**

519. Simmonds MP, Murray L (1998) The Atlantic frontier: Britain’s last ocean wilderness. London, United Kingdom: The Whale and Dolphin Conservation Society and Greenpeace, UK. **marine mammal, Atlantic NE**

520. Sipilä T (1990) Lair structure and breeding habitat of the Saimaa ringed seal (*Phoca hispida saimensis* Nordq.) in Finland. Finn Game Res 47: 11-20. **marine mammal, Atlantic NE**

521. Sipilä T, Helle E, Hyvärinen H (1990) Distribution, population size and reproductivity of the Saimaa ringed seal (*Phoca hispida saimensis* Nordq.) in Finland, 1980-84. Finn Game Res 47: 3-10. **marine mammal, Atlantic NE**

522. Sipilä T, Hyvärinen H (1998) Status and biology of Saimaa (*Phoca hispida saimensis*) and Ladoga (*Phoca hispida ladogensis*) ringed seals. NAMMCO Scientific Publications 1: 83-99. **marine mammal, Atlantic NE**

523. Skov H, Thomsen F (2008) Resolving fine-scale spatio-temporal dynamics in the harbour porpoise *Phocoena phocoena*. Mar Ecol Prog Ser 373: 14. **marine mammal, Atlantic NE**

524. Small JA, Small GJ (1991) Cetacean observations from the Somali Democratic Republic, September 1985 through May 1987. In: Leatherwood S, Donovan GP, editors. Cetaceans and cetacean research in the Indian Ocean Sanctuary, marine mammal technical report number 3: United Nations Environment Program (UNEP). pp. 179-210. **marine mammal, Indian W**

525. Smith BD, Beasley I, Buccat M, Calderon V, Evina R, et al. (2004) Status, ecology and conservation of Irrawaddy dolphins (*Orcaella brevirostris*) in Malampaya Sound, Palawan, Philippines. J Cetacean Res Manag 6: 41-52. **marine mammal, Pacific CW**

526. Sohn H, Kim ZG, Miyashita T, Park KJ (2001) Cruise report of the Korean whale sighting survey in the Yellow Sea, April-May 2001. Paper SC/53/RMP presented to the International Whaling Commission Scientific Committee. **Pacific NW, marine mammal**

527. Soldevilla MS, Wiggins SM, Calambokidis J, Douglas A, Oleson EM, et al. (2006) Marine mammal monitoring and habitat investigations during CalCOFI surveys. Marine Mammal Monitoring CalCOFI Report 47: 79-91. **marine mammal, Pacific CE**

528. Song of the Whale Research Team (2007) Some results of cetacean surveys between 2003 and 2007 in the Mediterranean’s western and eastern basins. ACCOBAMS - third meeting of the Contracting Parties, 22-25 October 2007. Dubrovnik, Croatia: ACCOBAMS. pp. 1-22. **marine mammal, Mediterranean**

529. Soto-Azat C, Boher F, Fabry M, Pascual P, Medina-Vogel G (2008) Surgical implantation of intra-abdominal radiotransmitters in marine otters (*Lontra felina*) in central Chile. J Wildl Dis 44: 979-982. **marine mammal, Pacific SE**

530. Southwest Fisheries Science Center (2007) Cruise report: NOAA Ship David Starr Jordan, DS-06-03, May 5 - June 18, 2006, rockfish recruitment assessment. Santa Cruz, CA: NOAA-NMFS-SWFSC-FED. **marine mammal, seabird, Pacific CE**

531. Spiegelberger T, Ganslosser U (2005) Habitat analysis and exclusive bank feeding of the Antillean manatee (*Trichechus manatus manatus* L. 1758) in the Coswine swamps of French Guiana, South America. Trop Zool 18: 1-12. **marine mammal, Atlantic CW**

532. Stafford KM, Mellinger DK, Moore SE, Fox CG (2007) Seasonal variability and detection range modeling of baleen whale calls in the Gulf of Alaska, 1999-2002. J Acoust Soc Am 122: 3378-3390. **marine mammal, Pacific NE**

533. Steiner L, Silva MA, Zereba J, Leal MJ (2007) Bryde's whales, *Balaenoptera edeni*, observed in the Azores: A new species record for the region. JMBA2 Biodiversity Records 5728: 1-6. **marine mammal, Atlantic NE**

534. Stewart BS, Karl SA, Yochem PK, Leatherwood S, Laake JL (1987) Aerial surveys for cetaceans in the former Akutan, Alaska, whaling grounds. Arctic 40: 33–42. **marine mammal, Pacific NE**

535. Stockin KA, Visser IN (2005) Anomalously pigmented common dolphins (*Delphinus* sp.) off northern New Zealand. Aquat Mamm 31: 43-51. **marine mammal, Atlantic SW**

536. Stockin KA, Weir CR, Pierce GJ (2006) Examining the importance of Aberdeenshire (UK) coastal waters for North Sea bottlenose dolphins (*Tursiops truncatus*). J Mar Biol Assoc U K 86: 201-207. **marine mammal, Atlantic NE**

537. Stoneburner DL (1982) Activities of juvenile green turtles, *Chelonia mydas*, at a jettied pass in south Texas. Copeia 1982: 400-408. **sea turtle, Atlantic CW**

538. Strick JM, Fritz LW, Lewis JP (1997) Aerial and ship-based surveys of Steller sea lions (*Eumetopias jubatus*) in southeast Alaska, the Gulf of Alaska, and Aleutian Islands during June and July 1994. Seattle, WA: NOAA Technical Memorandum NMFS-AFSC-71. **marine mammal, Pacific NE**

539. Suydam RS, George JC (1992) Recent sightings of harbor porpoises, *Phocoena phocoena*, near Point Barrow, Alaska. Can Field-Nat 106: 489-492. **marine mammal, Pacific NE**

540. Suydam RS, Lowry LF, Frost KJ (2005) Distribution and movements of beluga whales from the eastern Chukchi Sea stock during summer and early autumn. OCS Study Report MMS 2005-035. **marine mammal, Pacific NE, Arctic**

541. Suydam RS, Lowry LF, Frost KJ, O'Corry-Crowe GM, Pikok D (2001) Satellite tracking of eastern Chukchi Sea beluga whales into the Arctic Ocean. Arctic 54: 237-243. **marine mammal, Arctic**

542. Swartz SL, Cole T, McDonald D, Hildebrand JA, Oleson EM, et al. (2003) Acoustic and visual survey of humpback whale (*Megaptera novaeangliae*) distribution in the eastern and southeastern Caribbean Sea. Caribb J Sci 39: 195-208. **marine mammal, Atlantic CW**

543. Swartz SL, Martinez A, Cole T, Clapham PJ, McDonald MA, et al. (2001) Visual and acoustic survey of humpback whales (*Megaptera novaeangliae*) in the eastern and southern Caribbean Sea: Preliminary findings. Miami, FL: NOAA Technical Memorandum NMFS-SEFSC-456. **marine mammal, Atlantic CW**

544. Tasker MI (1984) Marine mammal records 1979-1983. North Sea Bird Club Report 4: 52-54. **marine mammal, Atlantic NE**

545. Taylor B (2008) Vaquita expedition 2008: Buoy launching and visual surveys. La Jolla, CA: NOAA-NMFS-SWFSC and SEMARNET Instituto Nacional de Ecologia. **marine mammal, Pacific NE**

546. Teilmann J, Born EW, Acquarone M (1999) Behaviour of ringed seals tagged with satellite transmitters in the North Water Polynya during fast-ice formation. Can J Zool 77: 1934-1946. **marine mammal, Atlantic NE**

547. Thomas L, Marques T, Borchers D, Harris C, Moretti D, et al. (2007) DECAF – Density estimation for cetaceans from passive acoustic fixed sensors: Annual report 2007. Washington, DC: National Oceanographic Partnership Program. **marine mammal, Atlantic CW**

548. Thomas L, Marques T, Borchers D, Harris C, Moretti D, et al. (2008) DECAF – Density estimation for cetaceans from passive acoustic fixed sensors: Annual report 2008. Washington, DC: National Oceanographic Partnership Program. **marine mammal, Atlantic CW**

549. Thompson D, Duck CD, editors (1995) Southern sea lions (*Otaria flavescens*) in the Falkland Islands: Population size, foraging behaviour and diet. Cambridge, UK: Sea Mammal Research Unit. **marine mammal, Atlantic SW**

550. Thompson D, Duck CD, McConnell BJ, Garrett J (1998) Foraging behaviour and diet of lactating female southern sea lions (*Otaria flavescens*) in the Falkland Islands. J Zool 246: 135-146. **marine mammal, Atlantic SW**

551. Thomsen F, Laczny M, Piper W (2007) Spatial and temporal occurrence of 'transient' cetaceans in the German Bight in 2001-2005. 17th biennial conference on the Biology of Marine Mammals (SMM), 29 November - 3 December 2007. Capetown, South Africa. **marine mammal, Atlantic NE**

552. Tiemann CO, Martin SW, Mobley JR (2006) Aerial and acoustic marine mammal detection and localization on Navy ranges. IEEE J Oceanic Eng 31: 107-119. **marine mammal, Pacific CE**

553. Tossy AH, Khan U, Mahmood R, Bhagat HB (2009) First tagging with a radio-transmitter of a rescued Indus River dolphin near Sukkur Barrage, Pakistan. Wildlife Middle East 3: 6. **marine mammal, Indian W**

554. Townsend CH (1931) Where the nineteenth century whaler made his catch. Zoologica 34: 173-179. **marine mammal, Arctic, Pacific NE, Pacific NW, Pacific CW, Pacific CE, Pacific SW, Pacific SE, Atlantic NW, Atlantic CW, Atlantic SW, Atlantic NE, Atlantic CE, Atlantic SE, Indian W, Indian E**

555. Townsend CH (1935) The distribution of certain whales as shown by logbook records of American whale ships. Zoologica 19: 1-50. **marine mammal, Arctic, Pacific NE, Pacific NW, Pacific CW, Pacific CE, Pacific SW, Pacific SE, Atlantic NW, Atlantic CW, Atlantic SW, Atlantic NE, Atlantic CE, Atlantic SE, Indian W, Indian E**

556. Treacy SD, Gleason JS, Cowles CJ (2006) Offshore distances of bowhead whales (*Balaena mysticetus*) observed during fall in the Beaufort Sea, 1982–2000: An alternative interpretation. Arctic 59: 83-90. **marine mammal, Arctic**

557. Trujillo F, Portocarrero M, Gomez C, Pardo D, Marques F, et al. (2007) Abundance estimates of the river dolphins *Inia geoffrensis*, *Inia boliviensis* and *Sotalia fluviatilis* in the Amazon and Orinoco basins (Venezuela, Colombia, Peru, Ecuador and Bolivia): A regional initiative. 17th biennial conference on the Biology of Marine Mammals (SMM), 29 November - 3 December 2007. Capetown, South Africa. **marine mammal, Atlantic CW, Pacific SE**

558. Turvey ST, Pitman RL, Taylor BL, Barlow J, Akamatsu T, et al. (2007) First human-caused extinction of a cetacean species? Biol Lett 3: 537-540. **marine mammal, Pacific NW**

559. Tynan CT, DeMaster DP, Peterson WT (2001) Endangered right whales on the southeastern Bering Sea shelf. Science 294: 1894-1894. **marine mammal, Pacific NE**

560. Udevitz MS, Bodkin JL, Costa DP (1995) Detection sea otters in boat-based surveys of Prince William Sound, Alaska. Mar Mamm Sci 11: 59-71. **marine mammal, Pacific NE**

561. Urban J, Guerrero-Ruiz M, Gendron D, Rodriguez E (2007) Current knowledge of killer whales in the gulf of California. 17th biennial conference on the Biology of Marine Mammals (SMM), 29 November - 3 December 2007. Capetown, South Africa. **marine mammal, Pacific CE**

562. US Fleet Forces (2008) Jacksonville range complex draft environmental impact statement/overseas environmental impact statement (EIS/OEIS). **marine mammal, Atlantic CW**

563. Van Dam RP, Kooyman GL (2004) Latitudinal distribution of penguins, seals and whales observed during a late autumn transect through the Ross Sea. Antarct Sci 16: 313-318. **seabird, marine mammal, Atlantic Antarctic**

564. van der Meij SET, Camphuysen CJ (2006) The distribution and diversity of whales and dolphins (Cetacea) in the southern North Sea: 1970-2005. Lutra 49: 3-28. **marine mammal, Atlantic NE**

565. Van Franeker JA, Gavrilo M, Mehlum F, Veit RR, Woehler EJ (1999) Distribution and abundance of the Antarctic petrel. Waterbirds 22: 14-28. **seabird, Pacific Antarctic, Atlantic Antarctic, Indian Antarctic**

566. VanderWerf EA, Elliott L, Fretz JS (2005) Observations on the abundance and behavior of seabirds south of O'ahu during the F/V Ehime Maru relocation and fuel spill. Elepaio 65: 25, 27-28, 29. **seabird, Pacific CE**

567. Veit RR, Pyle PP, McGowan JA (1996) Ocean warming and long-term change in pelagic bird abundance within the California current system. Mar Ecol Prog Ser 139: 11-18. **seabird, Pacific CE**

568. Vertyankin VV, Nladimirov VA, Tyurneva OY, Yakovlev YM, Burkanov VN (2007) New western gray whale (*Eschrichtius robustus*) feeding area offshore eastern Kamchatka, Russia. 17th biennial conference on the Biology of Marine Mammals (SMM), 29 November - 3 December 2007. Cape Town, South Africa. **marine mammal, Pacific NW**

569. Vieira N, Brito C (2009) Past and recent sperm whales sightings in the Azores based on catches and whale watching information. J Mar Biol Assoc U K 89: 1067-1070. **marine mammal, Atlantic NE**

570. Vigness Raposa K (2007) Spatial distribution of humpback whale sightings and survey effort in the North Atlantic Ocean. 17th biennial conference on the Biology of Marine Mammals (SMM), 29 November - 3 December 2007. Capetown, South Africa. **marine mammal, Atlantic NW**

571. Villegas MJ, Aron A, Ebensperger LA (2007) The influence of wave exposure on the foraging activity of marine otter, *Lontra felina* (Molina, 1782) (Carnivora: Mustelidae) in northern Chile. J Ethol 25: 281-286. **marine mammal, Pacific SE**

572. Violaine D, Virginie B, Bernard R (2007) Cetacean diversity off La Reunion Island (France), implication for the new marine protected area. 17th biennial conference on the Biology of Marine Mammals (SMM), 29 November - 3 December 2007. Capetown, South Africa. **marine mammal, Indian W**

573. Visser IN (1999) Antarctic orca in New Zealand waters? N Z J Mar Freshw Res 33: 515-520. **marine mammal, SW Atlantic**

574. Visser IN (2005) First observations of feeding on thresher (*Alopias vulpinus*) and hammerhead (*Sphyrna zygaena*) sharks by killer whales (*Orcinus orca*) specialising on elasmobranch prey. Aquat Mamm 31: 83-88. **marine mammal, Atlantic SW**

575. Visser IN, Bonoccorso FJ (2003) New observations and a review of killer whale (*Orcinus orca*) sightings in Papua New Guinea waters. Aquat Mamm 29: 150-172. **marine mammal, Pacific CW**

576. Visser IN, Fertl D, Pusser LT (2004) Melanistic southern right-whale dolphins (*Lissodelphis peronii*) off Kaikoura, New Zealand, with records of other anomalously all-black cetaceans. N Z J Mar Freshw Res 38: 833-836. **marine mammal, Pacific SW**

577. Vladimirov V, Vladimirov A, Blokhin S, Doroshenko N, Maminov M, et al. (2007) Spatiotemporal distribution and status of western gray whale congregation off northeast Sakhalin, Russia, 2004-2006. 17th biennial conference on the Biology of Marine Mammals (SMM), 29 November - 3 December 2007. Capetown, South Africa. **marine mammal, Pacific NW**

578. Vladimirov VL (1994) Recent distribution and abundance level of whales in Russian far-eastern seas. Russ J Mar Biol 20: 1-9. **marine mammal, Pacific NW**

579. Volkov AF, Moroz IF (1977) Oceanological conditions of the distribution of cetacea in the eastern tropical part of the Pacific Ocean. Rep Int Whaling Comm 27: 186-194. **marine mammal, Pacific SE**

580. Wade P, Heide-Jorgensen MP, Shelden K, Barlow J, Carretta J, et al. (2006) Acoustic detection and satellite-tracking leads to discovery of rare concentration of endangered North Pacific right whales. Biol Lett 2: 417-419. **marine mammal, Pacific NE**

581. Wade PR, Gerrodette T (1992) Estimates of dolphin abundance in the eastern tropical Pacific: Preliminary analysis of five years of data. Rep Int Whaling Comm 42: 533-539. **marine mammal, Pacific SE**

582. Wade PR, Gerrodette T (1993) Estimates of cetacean abundance and distribution in the eastern tropical Pacific. Rep Int Whaling Comm 43: 477-493. **marine mammal, Pacific SE**

583. Wang J, Yang S, Hung S, Jefferson T (2007) Distribution and abundance of the eastern Taiwan Strait population of Indo-Pacific humpback dolphins. 17th biennial conference on the Biology of Marine Mammals (SMM), 29 November - 3 December 2007. Capetown, South Africa. **marine mammal, Pacific NW**

584. Wang KX, Wang D, Zhang XF, Pfluger A, Barrett L (2006) Range-wide Yangtze freshwater dolphin expedition: The last chance to see Baiji? Environ Sci Pollut Res Int 13: 418-424. **marine mammal, Pacific NW**

585. Ward J, Morrissey R, Moretti D, DiMarzio N, Jarvis S, et al. (2008) Passive acoustic detection and localization of *Mesoplodon densirostris* (Blainville's beaked whale) vocalizations using distributed bottom-mounted hydrophones in conjunction with a digital tag (DTag) recording. Can Acoust 36: 60-66. **marine mammal, Atlantic CW**

586. Ward JA, Mitchell GH, Farak AM, Keane EP (2005) Beaked whale habitat characterization and prediction Newport, RI: NUWC-NPT Technical Report 11, 548. **marine mammal, Atlantic NW**

587. Waring GT, Hamazaki T, Sheehan D, Wood G, Baker S (2001) Characterization of beaked whale (Ziphiidae) and sperm whale (*Physeter macrocephalus*) summer habitat in shelf-edge and deeper waters off the northeast U.S. Mar Mamm Sci 17: 703-717. **marine mammal, Atlantic NW**

588. Waring GT, Nottestad L, Olsen E, Skov H, Vikingsson G (2008) Distribution and density estimates of cetaceans along the mid-Atlantic ridge during summer 2004. J Cetacean Res Manag 10: 137-146. **marine mammal, Atlantic NE**

589. Wei Z, Wang D, Zhang X-F, Wang K-X, Gao D-B (2004) Aggregation and spatio-temporal distribution of the Yangtze finless porpoise *Neophocaena phocaenoides asiaeorientalis* in Tian-E-Zhou National Baiji Reserve. Acta Hydrobiol Sin 28: 247-252. **marine mammal, Pacific NW**

590. Weir CR (1999) Cetacean surveys in the Atlantic frontier. Soundings 5: 5-7. **marine mammal, Atlantic NE**

591. Weir CR (2000) Sightings of beaked whale species (Cetacea: Ziphiidae) in the waters to the north and west of Scotland and the Faroe Islands. Eur Res Cetacean 14: 239-243. **marine mammal, Atlantic NE**

592. Weir CR (2001) Sightings of marine mammals and other animals recorded from offshore installations in the North Sea. In: Thorpe AW, editor. North Sea Bird Club 21st Anniversary Report. pp. 93-103. **marine mammal, Atlantic NE**

593. Weir CR (2001) A report on the whales, dolphins and seabirds of the Bay of Biscay and English Channel, ORCA Report. ORCA 1: 61-80. **marine mammal, Atlantic NE**

594. Weir CR (2006) First confirmed records of Clymene dolphin, *Stenella clymene* (Gray, 1850), from Angola and Congo, south-east Atlantic Ocean. Afr Zool 41: 297-300. **marine mammal, Atlantic SE**

595. Weir CR (2007) Occurrence and distribution of cetaceans off northern Angola, 2004/05. J Cetacean Res Manag 9: 225-239. **marine mammal, Atlantic SE**

596. Weir CR (2008) Overt responses of humpback whales (*Megaptera novaeangliae*), sperm whales (*Physeter macrocephalus*), and Atlantic spotted dolphins (*Stenella frontalis*) to seismic exploration off Angola. Aquat Mamm 34: 71-83. **marine mammal, Atlantic SE**

597. Weir CR, Debrah J, Ofori-Danson PK, Pierpoint C, Van Waerebeek K (2008) Records of Fraser's dolphin *Lagenodelphis hosei* Fraser 1956 from the Gulf of Guinea and Angola. Afr J Mar Sci 30: 241-246. **marine mammal, Atlantic SE, Atlantic CE**

598. Weir CR, Pollock C, Cronin C, Taylor S (2001) Cetaceans of the Atlantic frontier, north and west of Scotland. Cont Shelf Res 21: 1047-1071. **marine mammal, Atlantic NE**

599. Weir CR, Ron T, Morais M, Duarte ADC (2007) Nesting and at-sea distribution of marine turtles in Angola, west Africa, 2000-2006: Occurrence, threats and conservation implications. Oryx 41: 224-231. **sea turtle, Atlantic SE**

600. Weir CR, Stockin KA (2001) The occurrence and distribution of bottlenose dolphins (*Tursiops truncatus*) and other cetacean species in the coastal waters of Aberdeenshire, Scotland. Oxford: Sea Watch Foundation. **marine mammal, Atlantic NE**

601. Weir CR, Stokes J, Martin C, Cermeño P (2004) Three sightings of Mesoplodon species in the Bay of Biscay: First confirmed True's beaked whales (*M. mirus*) for the north-east Atlantic. J Mar Biol Assoc U K 84: 1095-1099. **marine mammal, Atlantic NE**

602. Weller DW, Burdin AM, Bradford AL, Ivashchenko YV, Tsidulko GA, et al. (2004) Western gray whales off Sakhalin Island, Russia: A joint Russia - U.S. scientific investigation July-September 2003: Final research report. La Jolla, CA; Petropavlovsk, Russia; Seaward, AK: NOAA-NMFS-SWFSC; Kamchatka Branch of Pacific Institute of Geography, Russian Academy of Sciences; Alaska Sealife Center. **marine mammal, Pacific NW**

603. Weller DW, Würsig B, Bradford AL, Burdin AM, Blokhin SA, et al. (1999) Gray whales (*Escherichtius robustus*) off Sakhalin Island, Russia: Seasonal and annual patterns of occurrence. Mar Mamm Sci 15: 1208–1227. **marine mammal, Pacific NW**

604. Weller DW, Würsig B, Burdin AM, Reeve SH, Bradford AL (2000) Gray whales off Sakhalin Island, Russia: June - October 1999: A joint U.S. - Russian scientific investigation. Final contract report for Sakhalin Marine Mammal Monitoring and Research Program, Sakhalin Energy Investment Company. College Station, Texas and Kamchatka, Russia: Texas A&M University and Kamchatka Institute of Ecology and Nature Management. **marine mammal, Pacific NW**

605. Western Gray Whale Advisory Panel (2008) Report of the Western Gray Whale Advisory Panel at its fifth meeting, 3-6 December 2008. Lausanne, Switzerland: WGWAP-5. **marine mammal, Pacific NW**

606. Whaley AR, Wright AJ, Bonnelly de Calventi I, Parsons ECM (2007) Humpback whale sightings in southern waters of the Dominican Republic lead to proactive conservation measures. JMBA2 Biodiversity Records 5751: 1-4. **marine mammal, Atlantic CW**

607. White RW, Reid JB, Black AD, Gillon KW (1999) Seabird and marine mammal dispersion in the waters around the Falkland Islands 1998–1999. Peterborough, UK: Joint Nature Conservation Committee. **marine mammal, seabird, Atlantic NE**

608. Whitehead H, Chu K, Harcourt P, Alling A (1982) The humpback whales off west Greenland: Summer 1981. Final Report to Marine Mammal Commission. Springfield, VA: NTIS Publication Number PB82-243924. **marine mammal, Atlantic NW**

609. Whitt A, Jefferson T, Ferti D, Rees D (2007) An annotated checklist of marine mammals of Cuba. 17th biennial conference on the Biology of Marine Mammals (SMM), 29 November - 3 December 2007. Capetown, South Africa. **marine mammal, Atlantic CW**

610. Wiig O, Gjertz I, Griffiths D (1996) Migration of walruses (*Odobenus rosmarus*) in the Svalbard and Franz Josef Land area. J Zool 238: 769-784. **marine mammal, Atlantic NE**

611. Wiig O, Gjertz I, Griffiths D, Lydersen C (1993) Diving patterns of an Atlantic walrus *Odobenus rosmarus rosmarus* near Svalbard. Polar Biol 13: 71-72. **marine mammal, Atlantic NE**

612. Wiley DN, Asmutis RA, Pitchford TD, Gannon DP (1995) Stranding and mortality of humpback whales, *Megaptera novaeangliae*, in the mid-Atlantic and southeast United States, 1985-1992. Fish Bull 93: 196-205. **marine mammal, Atlantic NW, Atlantic CW**

613. Wiley DN, Moller JC, Zilinskas KA (2003) The distribution and density of commercial fisheries and baleen whales within the Stellwagen Bank National Marine Sanctuary: July 2001-June 2002. Mar Technol Soc J 37: 35-53. **marine mammal, Atlantic NW**

614. Williams AD, Brereton T, Williams R (1999) Seasonal variation in the occurrence of beaked whales in southern Bay of Biscay. Eur Res Cetacean 13: 275-277. **marine mammal, Atlantic NE**

615. Williams R, Thomas L (2009) Cost-effective abundance estimation of rare animals: Testing performance of small-boat surveys for killer whales in British Columbia. Biol Conserv 142: 1542-1547. **marine mammal, Pacific NE**

616. Wilson B, Thompson PM, Hammond PS (1997) Habitat use by bottlenose dolphins: Seasonal distribution and stratified movement patterns in the Moray Firth, Scotland. J Appl Ecol 34: 1365-1374. **marine mammal, Atlantic NE**

617. Winter A, Foy RJ, Wynne K (2009) Seasonal differences in prey availability around a Steller sea lion haulout and rookery in the Gulf of Alaska. Aquat Mamm 35: 145-162. **marine mammal, Pacific NE**

618. Witzell WN, Azarovitz T (1996) Relative abundance and thermal and geographic distribution of sea turtles off the U.S. Atlantic coast based on aerial surveys (1963-1969). Miami, FL: NOAA Technical Memorandum NMFS-SEFSC-381. **sea turtle, Atlantic NW, Atlantic CW**

619. Womble JH, Sigler MF, Wilson MF (2009) Linking seasonal distribution patterns with prey availability in a central-place forager, the Steller sea lion. J Biogeogr 36: 439-451. **marine mammal, Pacific NE**

620. Womble JN, Gende SM, Blundell GM (2007) Dive behavior of a harbor seal (*Phoca vitulina richardii*) in the presence of transient killer whales (*Orcinus orca*) in Glacier Bay National Park, Alaska. Mar Mamm Sci 23: 203-208. **marine mammal, Pacific NE**

621. Womble JN, Willson MF, Sigler MF, Kelly BP, VanBlaricom GR (2005) Distribution of Steller sea lions *Eumetopias jubatus* in relation to spring-spawning fish in SE Alaska. Mar Ecol Prog Ser 294: 271-282. **marine mammal, Pacific NE**

622. Yakovlev Y, Tyurneva O (2006) Photographic identification of the Okhotsk-Korean gray whale (*Eschrichtius robustus*) along northeast Sakhalin Island, Russia, 2005. Final report. Yuzhno-Sakhalinsk, Russia: Exxon Neftegas Limited and Sakhalin Energy Investment Company Ltd. **marine mammal, Pacific NW**

623. Yang J, Xiao W, Kuang X-A, Wei Z, Liu R-J (2000) Studies on the distribution, population size and the active regularity of *Lipotes vexillifer* and *Neophocaena phocaenoides* in Dongting Lake and Boyang Lake. Resour Environ Yangtze Basin 9: 443-450. **marine mammal, Pacific NW**

624. Yang S-C, Liao H-C, Pan C-L, Wang J (1999) A survey of cetaceans in the waters of central-eastern Taiwan. Asian Mar Biol 16: 23-34. **marine mammal, Pacific NW**

625. Yazvenko SB, McDonald TL, Blokhin SA, Johnson SR, Meier SK, et al. (2007) Distribution and abundance of western gray whales during a seismic survey near Sakhalin Island, Russia. Environ Monit Assess 134: 45–73. **marine mammal, Pacific NW**

626. Yen PPW, Huettmann F, Cooke F (2004) A large-scale model for the at-sea distribution and abundance of marbled murrelets (*Brachyramphus marmoratus*) during the breeding season in coastal British Columbia, Canada. Ecol Model 171: 395-413. **seabird, Pacific NE**

627. Yen PPW, Sydeman WJ, Bograd SJ, Hyrenbach KD (2006) Spring-time distributions of migratory marine birds in the southern California Current: Oceanic eddy associations and coastal habitat hotspots over 17 years. Deep Sea Res Part II 53: 399-418. **seabird, Pacific CE**

628. Yen PPW, Sydeman WJ, Hyrenbach KD (2004) Marine bird and cetacean associations with bathymetric habitats and shallow-water topographies: Implications for trophic transfer and conservation. J Marine Syst 50: 79-99. **seabird, marine mammal, Pacific CE**

629. Yen PPW, Sydeman WJ, Morgan KH, Whitney FA (2005) Top predator distribution and abundance across the eastern Gulf of Alaska: Temporal variability and ocean habitat associations. Deep Sea Res Part II 52: 799-822. **seabird, Pacific NE**

630. Zerbini AN, Andriolo A, da Rocha JM, Simoes-Lopes PC, Siciliano S, et al. (2004) Winter distribution and abundance of humpback whales (*Megaptera novaeangliae*) off northeastern Brazil. J Cetacean Res Manag 6: 101-107. **marine mammal, Atlantic SW**

631. Zerbini AN, Andriolo A, Heide-Jorgensen MP, Pizzorno JL, Maia YG, et al. (2006) Satellite-monitored movements of humpback whales *Megaptera novaeangliae* in the southwest Atlantic Ocean. Mar Ecol Prog Ser 313: 295-304. **marine mammal, Atlantic SW**

632. Zerbini AN, da Rocha JM, Andriolo A, Siciliano S, Moreno IB, et al. (2000) An outline of the cetacean sighting surveys conducted off the northeastern Brazilian coast with the preliminary abundance estimation of minke whales. Paper SC/52/IA18 presented to the International Whaling Commission Scientific Committee. 52nd IWC Annual Meeting, June 2000. Adelaide, Australia. **marine mammal, Atlantic CW**

633. Zhang X, Wang D, Liu R, Wei Z, Hua Y, et al. (2003) The Yangtze River dolphin or baiji (*Lipotes vexillifer*): Population status and conservation issues in the Yangtze River, China. Aquat Conserv: Mar Freshwat Ecosyst 13: 51-64. **marine mammal, Pacific NW**

634. Zhao XJ, Barlow J, Taylor BL, Pitman RL, Wang KX, et al. (2008) Abundance and conservation status of the Yangtze finless porpoise in the Yangtze River, China. Biol Conserv 141: 3006-3018. **marine mammal, Pacific NW**

635. Zhou K, Sun J, Gao A, Wursig B (1998) Baiji (*Lipotes vexillifer*) in the lower Yangtze River: Movements, numbers threats and conservation needs. Aquat Mamm 24: 123-132. **marine mammal, Pacific NW**

636. ZhouKaiya, Qian W, Li Y (1977) Studies on the distribution of baiji, *Lipotes vexillifer* Miller. Acta Hydrobiol Sin 23: 72-79. **marine mammal, Pacific NW**
